# Cancer Metastases to the Hand: A Systematic Review and Meta-analysis

**DOI:** 10.1177/15589447231153175

**Published:** 2023-03-01

**Authors:** Todd Dow, Caitlin Davis, Rawan ElAbd, Donald Lalonde, Jason Williams

**Affiliations:** 1 Division of Plastic and Reconstructive Surgery, Dalhousie University, Halifax, NS, Canada; 2 Department of Obstetrics and Gynecology, Dalhousie Unviersity, Halifax, NS, Canada; 3 Division of Plastic and Reconstructive Surgery, McGill Univeristy, Montreal, Canada; 4 Division of Plastic and Reconstructive Surgery, Jaber Al Ahmed Al Jaber Al Sabah Hopsital, Surra, Kuwait; 5 Division of Plastic and Reconstructive Surgery, Dalhousie University, Saint John, NB, Canada

**Keywords:** hand, anatomy, digits, cancer, acrometastasis, survival

## Abstract

**Background:**

Metastatic lesions to the hand or wrist are rare and can mimic inflammatory and benign processes such as gout and infections. This often leads to misdiagnosis, underreporting, and delays in treatment. The purpose of this study was to examine all known cases of metastasis to the hand or wrist available in the literature and to analyze demographic trends, metastasis characteristics, and clinical course, and provide recommendations for management.

**Methods:**

An online systematic review of MEDLINE, Embase, PubMed, and the Cochrane Library from inception to January 7, 2022, was completed. Studies outlining the care of a patient with acrometastases of the hand were included. Data extracted included age, sex, site of primary tumor and metastasis, presence of other metastases, time from primary diagnosis to acrometastasis diagnosis, misdiagnosis, treatment, and survival.

**Results:**

Between 1889 and present, 871 lesions were described in 676 patients who met the inclusion criteria. There was no predilection for hand dominance or site of previous trauma. The mean age among patients was 59.5 (1.5-91) years, and male sex was more common (64.6%). The most common primary cancer source was the lung (39.2%), followed by the kidney (10.8%). The distal phalanx was the most frequently cited tumor location (33.7%). Mean survival after diagnosis of acrometastasis was 6.3 months (0.25-50) ± 11.5 months.

**Conclusion:**

Acrometastasis remains an uncommon presentation of metastatic disease with poor prognosis. Treatment currently focuses on pain management and optimizing functional outcomes. Our review led to the development of 7 treatment recommendations when managing these patients.

## Introduction

While distant cancer metastases are relatively common among patients with metastatic disease, acrometastases, defined as metastatic lesions that are located distal to the elbow or knee, are infrequently seen.^1^ Given the rarity of these lesions, and that they can mimic inflammatory and benign processes such as gout and chronic infections, misdiagnosis, underreporting, and delayed treatment are common. Their presence signifies disseminated metastatic disease and thus often carries a grim prognosis.

Handley is frequently cited as outlining the first known case of osseous acrometastasis in 1906; however, Hinterstoisser described a case of lung cancer metastasizing to the distal phalanx in a 59-year-old man in 1889.^2,3^ The first case series was published in 1958 by Kerin, who reviewed the 30 known cases at that time. Unfortunately, many reviews on this subject were confined to a short time frame, limited geographically or linguistically, were limited to bony cases, or simply did not accurately identify all available cases.^4-10^ Furthermore, owing to the rarity and relative heterogeneity of acrometastases, treatment recommendations and guidelines were not established.

The intention of this study was to perform a systematic review and analysis of all known cases in the literature of acrometastasis to the bony and soft tissues of the hand and wrist. The primary outcome was to assess and compare survival based on the location of the primary cancer or acrometastasis. The secondary objectives were to compare mean survival before 2000 versus after 2000; provide a comprehensive list of all known published cases of acrometastasis; and provide information on initial investigations, diagnosis, treatment, and median time from diagnosis of the primary tumor to the diagnosis of metastases in the hand. We also include 2 of our own clinical cases for illustration.

## Methods

### Protocol and Eligibility Criteria

This systematic review was conducted in accordance with the Preferred Reporting Items for Systematic Reviews and Meta-analysis guidelines.^11^ The population consisted of human patients diagnosed with a metastatic soft tissue or bony lesion(s) to hand, inclusive of carpal bones but not the radius or ulna. There were no limitations with respect to age, country of publication, or language.

### Search Methodology and Study Selection

A systematic literature search of PubMed, MEDLINE, Embase, and Cochrane Register electronic databases was conducted using words and Medical Subject Headings terms for “metastasis,” “hand,” AND/OR “bones of the hand and wrist” with the assistance of a medical librarian. Articles published from inception to January 7, 2022, were included, and all references of these were screened for inclusion. Two authors (T.D. and C.D.) independently completed the initial screening and full-text review. A third author (R.E.) resolved any conflicts. Detailed search strategies are presented in Supplemental Appendix A.

### Data Extraction

Data extraction was completed by 2 authors (T.D. and C.D.) using a Microsoft Excel spreadsheet. Data extracted included variables related to study characteristics (author; year, country, journal, and language of publication; study design) and population (age; sex; primary cancer; cell type; history of injury; hand dominance; initial investigations, diagnosis, and treatment; location of acrometastasis, history of previous malignancy, timeline from primary malignancy to diagnosis of acrometastasis, presence of other metastatic lesions, definitive treatment, and survival).

### Quality Assessment and Heterogeneity Analysis

Methodological qualities were assessed by the Cochrane Risk of Bias scale^12^ for randomized studies or by the methodological index for non-randomized studies (MINORS) scale for non-randomized studies or case series. The latter is a validated guideline, consisting of 8 items for noncomparative studies and 4 items for comparative studies. High-quality scores are defined as 10 or higher for noncomparative studies and 16 or higher for comparative studies.^13^

### Statistical Analysis

When available, categorical variables were summarized as counts and percentages, whereas continuous variables were presented as median and interquartile range for non-normally distributed variables or as mean and SDs for normally distributed variables. Univariate analysis was performed to compare outcomes between cancer groups and publications before and after the year 2000.

## Results

### Study Selection and Characteristics

**Figure 1.**
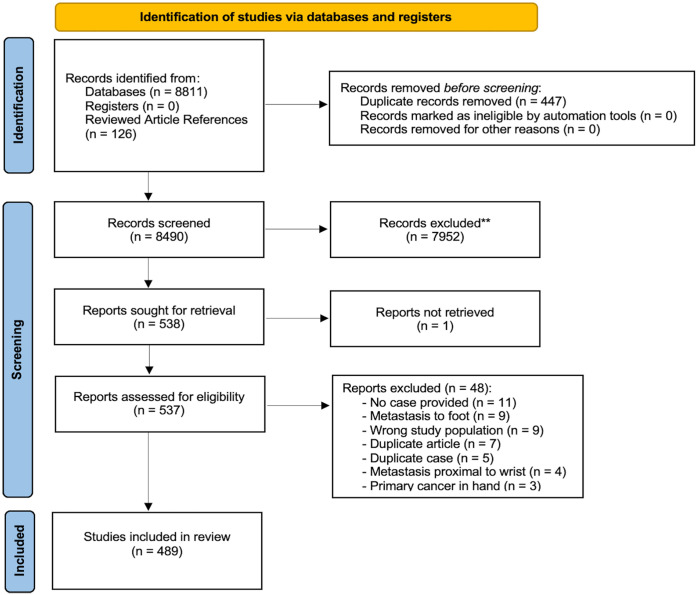
Flowchart of study inclusion using the Preferred Reporting Items for Systematic Reviews and Meta-analysis guidelines.^14^ **Studies excluded based on title and abstract screening.

A total of 8490 articles were obtained and 489 of these met the inclusion criteria (Figure 1). References for the included articles can be found in Supplemental Appendix B. The articles were published between 1889 and 2021 with the most recent decade yielding the greatest number of publications (31.9%). The vast majority were case reports (77.24%) (Supplemental Table 1). Cases were presented from 44 countries in 17 different languages, with the United States (28.8%) and English (78.3%) being the most common. The largest number of publications was within *The Journal of Hand Surgery*.

Cases presented within the retrospective review articles were not included in the population analysis as the case details could not be accurately assigned to the individual patients. Furthermore, these reviews often included hand and foot cases that could not be accurately separated. The details of these reviews are presented in Table 1. The majority, 57.1% (4/7), of the included studies were determined to not be of high methodological quality based on the MINORS instrument (Table 1).

**Table 1.** Summary of Retrospective Review Studies on Acrometastasis to the Hand and Wrist and Corresponding MINORS Scores.

Author	Year	Patients (n value)	Hand cases	Male (%)	Primary cancer (all cases)	Hand lesions	Survival (all cases)a	MINORS score
Healey et al^A483^	1986	27	18	NM	Lung (5), kidney (5), esophagus (4), colorectal (3), breast (2), ovary (2), prostate (2), bone (2), bladder (1), uterus (1), thyroid (1), unknown (1)	Trapezium (1), hamate (1), lunate (1), triquetrum, 1MC (1), 2MC (2), 3MC (1), 4MC (3), 5MC (2), (1)1DP (1), 1PP (2), 2PP (1), 3DP (1), 4DP (1), 4PP (1) 5DP (1)	8.4 ± 8.5	7
Leeson et al^A484^	1986	57	5	29 (50.9%)	Lung (17), kidney (8), neuroblastoma (5), prostate (4), thyroid (3), stomach (3), cervix (2), skin (2), breast (1), retinoblastoma (1), liver (1), rhabdomyosarcoma (1)	5 “hand”	6.7 (1-28)	7
Amandio and Lombardi^A485^	1987	18	18	14 (77.8%)	Lung (5), kidney (5), breast (1), head and neck (3), femur (1), humerus (1), colorectal (1)	1PP (1), 1DP (3), 2PP (1), 2DP (1), 3DP (4), 4DP(3), 5DP (1), 2MC (1), capitate (1), scaphoid (1), lunate (1), palm (ST)(2), dorsum of hand (ST)(2)	5.0	12
Libson et al^A486^	1987	21	21	13 (61.9%)	Lung (13), breast (4), kidney (1), prostate (1), colon (1), unknown (1)	“Carpal” (2), “Metacarpal” (5), “Phalange” (4), “Distal phalanx” (3), scaphoid (1), 3MC (1), 1PP (1), 1DP (3), 2PP (1), 2MP (1), 2DP (1), 3MP (1), 4MP (1), 4DP (1)	NM	6
Morris et al^A487^	2017	10	10	7 (70%)	Lung (4), skin (3), kidney (2), neuroendocrine (1)	Carpals (1), MCs (7), phalanges (5)	18 (5-138)	12
El Abiad et al^A488^	2019	28	12	16 (57.1%)	Lung (9), kidney (5), prostate (4), breast (2), soft tissue sarcoma (2), chondrosarcoma (1), squamous cell carcinoma (1), thyroid (1), metastatic chondroid syringoma (1), phosphaturic mesenchymal tumor (1)	Carpals (2), MCs (6), phalanges (6)	7.7 (1.3-144)	10
Tani et al^A489^	2020	11	NM	6 (54.5%)	Kidney (4), other (3), breast (2), colon (1), stomach (1)	Unclear	6.5	7

*Note.* ^A“reference number”^ = Citation for the reviewed article can be found in Supplemental Appendix B. MINORS = methodological index for non-randomized studies; M = male; F = female; NM = not mentioned; MC = metacarpal; DP = distal phalanx; PP = proximal phalanx; (ST) = indicates a soft tissue lesion; MP = middle phalanx; NS = no side mentioned.

a Mean, +/- SD or range in months if provided.

Within the remaining 491 articles, 591 unique cases were identified (Table 2). The mean age was 59.5 years (1.5-91 years) and male sex was more common (64.6%). The most frequent presenting concerns were a painful or swollen digit, hand, or wrist (86.6%). There was a history of trauma provided in 9.3% of cases. Of these, some were clearly delineated including a history of being struck with an object (1.7%), fall (1.0%), or dog bite (0.3%). However, insignificant trauma such as “pricked by a thorn” was the most common (3.5%) and many others were ill-defined (2.7%). Hand dominance was reported in only 3.6% of cases and corresponded to the side of the acrometastasis in 38.1% of those cases. The most common primary malignancy was lung (39.2%), kidney (10.8%), breast (7.9%), colorectal (7.9%), and esophageal (5.1%).

**Table 2.** Summary of Acrometastasis Patient Characteristics Organized by Primary Cancer.

Cancer groups	N value	Age, Mean, Y (range)	Male	Known history of malignancy	First diagnosis of primary cancer (prior mo)a	Other metastases before acrometastasis	Survival, mob
All patients	592	59.45 (1.5-91)	64.59% (374/579)	65.35% (364/557)	35.02 ± 50.78 (0.25-384), n = 315	37.80% (141/373)	6.27 ± 7.16 (0.25-50), n = 310
Lung	232	60.24 (10-89)	76.32% (174/228)	42.27% (93/220)	16.47 ± 37.09 (0.25-252), n = 74	18.60% (24/129)	5.61 ± 6.62 (0.25-50), n = 135
Oral	15	62 (40-72)	93.33% (14/15)	92.86% (13/14)	27.33 ± 21.70 (6-84), n = 12	54.55% (6/11)	4.41 ± 4.87 (0.25- 14), n = 8
Nasopharyngeal	7	63.29 (45-80)	85.71% (6/7)	85.71% (6/7)	59.17 ± 54.85 (12-160), n = 6	50.5% (3/6)	10.93 ± 9.41 (1.7-24), n = 4
Laryngeal	10	59.6 (52-70)	100% (10/10)	100.0% (10/10)	14.67 ± 8.47 (2-25), n = 9	11.11% (1/9%)	5.19 ± 4.39 (1-14), n = 8
Parotid	5	58.5 (41-68)	20.00% (1/5)	100.0% (4/4)	148 ± 184.48 (24-360), n = 3	100.0% (2/2)	12 ± 0 (12-12), n = 1
Thyroid	9	54.89 (32-84)	33.33% (3/9)	55.56% (5/9)	62.4 ± 24.59 (24-84), n = 5	28.57% (2/7)	2.5 ± 0.71 (2-3), n = 2
Breast	47	55.16 (30-91)	4.26% (2/47)	95.35% (41/43)	64.65 ± 74.74 (2-384), n = 39	59.38% (19/32)	6.6 ± 8.44 (0.25-39), n = 24
Liver	12	56.42 (30-70)	83.33% (10/12)	58.33% (7/12)	64.0 ± 60.97 (2-144), n = 6	62.5% (5/8)	5.19 ± 4.54 (0.5-15), n = 8
Esophageal	30	62.43 (38-85)	76.67% (23/30)	83.33% (25/30)	14.95 ± 21.44 (1-96), n = 21	42.11% (8/19)	5.14 ± 5.92 (0.5-24), n = 14
Gastric	16	61.81 (37-84)	62.5% (10/16)	68.75% (11/16)	20.45 ± 15.31 (2-48), n = 11	63.63% (7/11)	9.88 ± 11.2 (3-36), n = 8
Colorectal	47	65.09 (38-84)	55.56% (25/45)	88.89% (40/45)	34.06 ± 25.34 (4-120), n = 36	50.0% (15/30)	8.93 ± 9.22 (1-39), n = 22
Kidney	64	62.31 (45-89)	68.33% (41/60)	68.97% (40/58)	55.21 ± 68.87 (1-264), n = 34	45.0% (18/40)	7.22 ± 6.04 (1-24), n = 29
Bladder	10	57.70 (39-72)	80.0% (8/10)	66.67% (6/9)	41.00 ± 34 (7-96), n = 6	57.14% (4/7)	2.75 ± 1.25 (1-4), n = 4
Ovaries	3	51.00 (43-59)	0.0% (3/3)	66.67% (2/3)	8.5 ± 7.78 (3-14), n = 2	0.0% (0/3)	1.63 ± 1.94 (0.25-3), n = 2
Testes	3	58.00 (55-63)	100.0% (3/3)	83.33% (5/6)	3.33 ± 0.58 (3-4), n = 3	0.0% (0/3)	3.5 ± 0.71 (3-4), n = 2
Uterus	7	49.86 (31-74)	0.0% (0/7)	83.33% (5/6)	17.25 ± 13.94 (3-36), n = 4	50.0% (2/4)	5.75 ± 4.65 (1-12), n = 4
Prostate	10	62.00 (50-76)	100% (10/10)	70.0% (7/10)	27.5 ± 22.81 (7-60), n = 4	44.44% (4/9)	13.17 ± 17.83 (2-48), n = 6
Cervix/Vagina	8	60.88 (36-85)	0% (0/8)	87.5% (7/8)	5.71 ± 4.95 (0.5-12), n = 6	33.33% (2/6)	5.42 ± 6.84 (0.5-19), n = 6
Penile	2	67.00 (63-71)	100% (2/2)	100.0% (2/2)	15.0 ± 12.73 (6-24), n = 2	100% (1/1)	18 ± 0 (18-18), n = 1
Skeletal	12	41.09 (9-76)	72.73% (8/11)	100.0% (11/11)	38.6 ± 32.35 (12-120), n = 10	66.67% (6/9)	4.95 ± 7.36 (0.25-18), n = 5
Melanoma	6	40.17 (24-65)	16.67% (1/6)	83.33% (5/6)	34.2 ± 35.68 (5-96), n = 5	60.0% (3/5)	13 ± 0 (13-13), n = 1
Hematologic	10	60.30 (40-72)	30% (3/10)	77.78% (7/9)	41.0 ± 21.94 (6-72), n = 6	33.33% (2/6)	3.34 ± 2.61 (0.36-6), n = 4

a The values in this column are mean months between first diagnosis of primary to diagnosis of acrometastasis +/- SD (range), n value.

b The values in this column are mean survival in months from the time of acrometastasis diagnosis +/- SD (range), n value.

**Figure 2.**
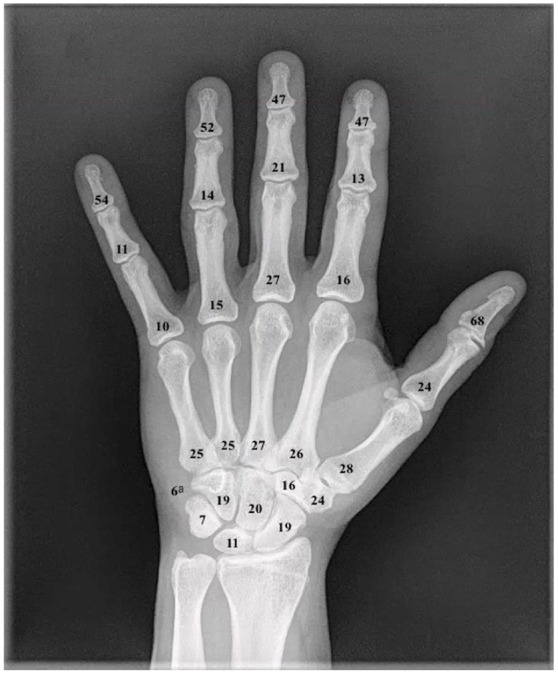
Graphic representation of cumulative bony metastatic lesions to the hand from case reports and case series. ^a^The pisiform.

The initial diagnosis of the hand or wrist lesion was described in 336 (56.8%) of cases, nearly half of which were misdiagnoses. The most common misdiagnosis was infection (abscess, felon, osteomyelitis, paronychia, “infection,” or tuberculosis) at 47.0%. Other incorrect diagnosis includes gout, as was the case with 1 of our patients on their initial presentation to a peripheral clinic (Figure 2). Traditional hand radiographs were used in 86.3% of cases, whereas other imaging modalities were less commonly ordered (bone scan [16.1%], magnetic resonance imaging [7.8%], positron emission tomography [PET] scan [4.4%], computed tomography [4.3%], and ultrasound [1.8%]). Some means of tissue biopsy was described in 58.0% of cases.

**Figure 3.**
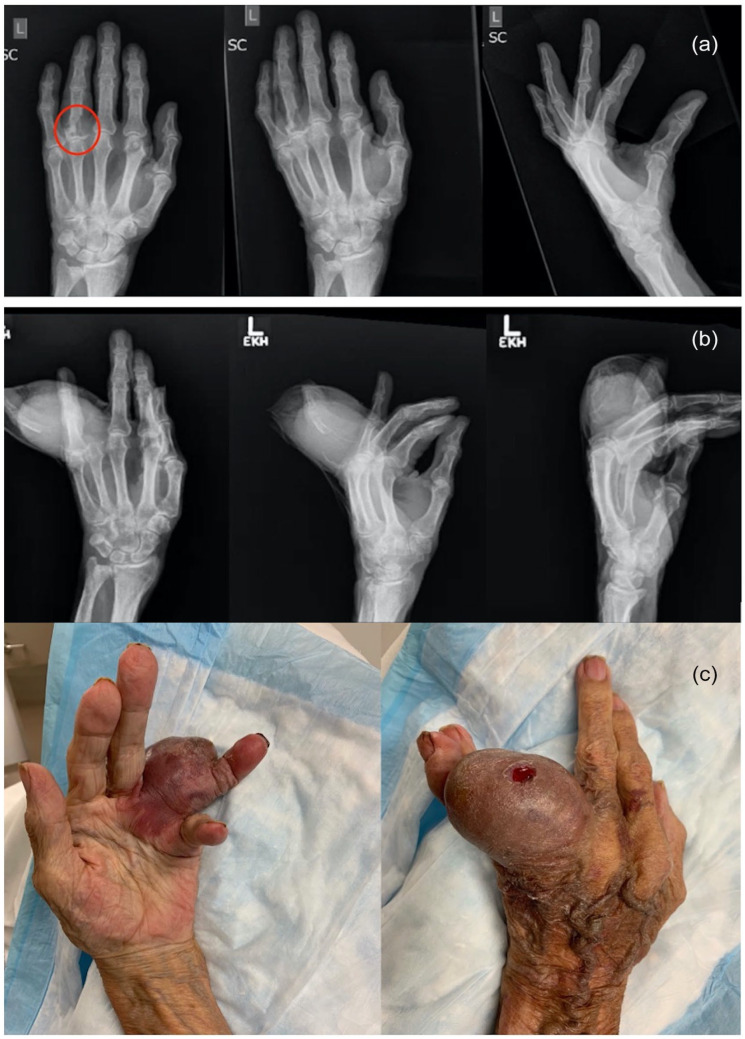
A 78-year-old man with metastatic renal cell carcinoma to the left fourth digit proximal phalanx. *Note*. (a) X-ray imaging at the time of presentation, incorrect diagnosis of gout. (b) X-ray imaging at the time of referral to hand surgery service 7 months later. (c) Clinical appearance of metastatic renal cell carcinoma.

There was no apparent lateralization, with 47.0% of cases isolated to the right hand or wrist and 42.4% to the left. Bilateral involvement was reported in 7.3%, whereas 3.4% did not specify the affected hand or wrist. When considering right-sided versus left-sided cancer of the breast, lung, or kidney, there was no correlation to ipsilateral metastatic hand lesion. Of the 871 lesions reviewed, 23 were not well described. Of those well described, 81.6% were bony and 18.4% were soft tissue. The phalanges were most commonly affected (51.8%) followed by metacarpals (15.5%) and carpals (14.4%; Figure 3). The distal phalanx was the most commonly affected bone (33.7%), with the distal phalanx of the thumb being the most common site overall (8.4%). The pisiform was the least common site, with 6 reported cases. The digital pulp was the most frequent site for soft tissue involvement making up 8.5% of all cases.

This predilection for the distal phalanx was observed across the most common primary malignancies and urologic, gynecologic, and thyroid malignancies. Interestingly, gastric cancers and melanomas metastasized more frequently to the carpals followed by metacarpals. Less common primaries, such as blood, skeletal, and parotid, had no clear pattern of distribution.

Most had a known primary cancer before presentation (65.4%; Table 2). However, 58.7% of patients with acrometastasis of lung origin had an occult lung malignancy. Another frequent occult malignancy was thyroid (44.4%) and liver (41.7%). For those providing a timeline of events, the mean time from diagnosis of primary cancer to acrometastasis diagnosis was 35 months (0.25-384 months). Three hundred seventy-three patients were found to have other distant metastasis, which were most commonly lung (35.5%) or liver (16.3%) metastases.

The mainstay of treatment was amputation or excision (58.0%), followed by radiotherapy (26.7%), and conservative/supportive and palliative care (13.3%). Use of amputation decreased as lesions became more proximal. Systemic treatment was reported in 71.5% of cases, with 38.1% of patients receiving palliative and comfort care, 36.6% receiving systemic chemotherapy or immunotherapy, and 30.7% receiving radiotherapy.

Survival outcome was reported in 73.3% of cases. Of these, 23.3% were alive at the time of publication; however, a dismal prognosis was often noted. Of the patients who died, the majority (93.1%) provided a survival timeline yielding a mean survival of 6.3 months (0.25-50 months) from the time of acrometastasis diagnosis. Of the most common primary malignancies, esophageal had the shortest mean survival of 5.1 months (0.5-24), followed by lung at 5.6 months (0.25-50). When comparing cancers of the reproductive organs (ovaries vs testes, uterus vs prostate, and cervix/vagina vs penis), female patients had a shorter mean survival (Table 2). There was a correlation between survival and greater time from diagnosis of primary cancer to diagnosis of acrometastasis.

Most cases provided histological analysis (82.6%), although most did not have sufficient numbers or variety of cell type to delineate any significant patterns (Supplemental Table 1). Lung adenocarcinoma presented much later than other lung cancers (29.6 vs 16.5 months). Large-cell carcinoma of the lung was also noted to have the worst prognosis of the lung cancer subtypes with average survival being 2.06 ± 1.53 months (0.25-4 months).

When comparing studies published before the year 2000 and those in the year 2000 and beyond, several trends were noted. The percentage of those opting for conservative versus invasive management remained stable over time. With respect to systemic treatment, there was a considerable increase in provision of chemotherapy or immunotherapy (24.6%-47.3%) and a decrease in radiotherapy (34.2%-27.7%) and palliation (45.7%-31.3%). Furthermore, survival significantly increased from 5.35 (±6.16) to 7.43 (±8.13) months (*P* = .014). Lung cancer and esophageal cancer also saw a significant increase in the mean age of patients presenting with acrometastasis (*P* = 0.001).

## Discussion

It is widely known that bone metastases most commonly affect the axial skeleton, and involvement of the hands or feet is rare. However, the number of case reports of acrometastases has been increasing, perhaps due to increased detection, increasing life expectancy, or incentive to publish unusual findings, and metastases have now been documented in every bone of the hand.^15,16^ The true incidence of acrometastasis is undoubtedly higher than that noted in our review due to subclinical lesions, misdiagnosis, lack of tissue confirmation, presentation in end-of-life patients, lack of postmortem investigations, and unreported cases.^6,17^ We have conducted the most comprehensive review to date encompassing both bony and soft tissue metastases under the umbrella term of acrometastasis.

Despite their rarity, acrometastasis remains noteworthy. Acrometastasis can be the first manifestation of an occult cancer, particularly lung and colorectal, as seen in 36.1% of the reviewed cases.^14^ Furthermore, we corroborated that these lesions typically present with pain and discomfort (86.7%), and that this can not only significantly affect quality of life and function but may also be the first sign of widespread disease.

Acrometastasis pathophysiology is not entirely understood but likely involves hematogenous transport of tumor emboli.^18,19^ Mulvey et al suggested that venous erosion by a pulmonary neoplasm would facilitate the dissemination of tumor emboli into the left atrium and ventricle and subsequently into systemic arterial circulation.^18^ Furthermore, other visceral tumor emboli cannot reach the arteries directly, making it unconventional for them to present as acrometastasis.^7,20^ This theory is supported by our data and explains why lung metastasis is by far the most common primary cancer metastasizing to the digits and why a significant portion (58.7%) of lung acrometastasis was the first sign of lung cancer or was identified at the time of lung cancer diagnosis. Similarly, patients who had an extrapulmonary primary malignancy with known metastatic lesions were most likely to have a pulmonary lesion, regardless of the site of the primary malignancy. The exceptions were gut and colorectal cancers that were noted to metastasize most commonly to the liver and then lungs, in accordance with the hematologic spread theory of these malignancies.

Interestingly, the frequency of metastatic pulmonary lesions was noted to increase over time in our patient population. Similarly, more patients were found to have known primary malignancy before presentation of acrometastasis (32.5%-43.4%). These findings are plausibly related to advancements in cancer diagnostics and surveillance and thus earlier detection of primary tumor. Regardless, 36.5% of patients did not have evidence of lung involvement even after acrometastasis diagnosis. Perhaps, this group did systemically disseminate metastatic emboli directly from an extrapulmonary neoplasm, or perhaps our investigations were unable to identify a culprit lung metastasis.

Similar to previous publications and the retrospective reviews we assessed, the distal phalanx was determined to be the most common site for metastasis (Figure 3).^1,14-16,21,22^ The distal phalanx, hyponychium, nail folds, and nail bed have dense capillary networks with a single layered rectangular plexus of capillaries in the plane of the nail matrix.^23,24^ Considering the aforementioned pathogenesis, increased vascular networks within the distal phalanx compared with the middle and proximal phalanx would make it more susceptible.^18,25,26^ This aligns with the “seed and soil” theory presented by Paget in 1889 and outlines how tumor emboli, such as seeds, search for appropriate tissue (soil) to grow.^27^ Historically, Piney described phalanges as being absent of marrow and thus unfavorable growth environments.^28^ Joll expanded on this and postulated that repeated trauma may influence acrometastasis by reducing inherent resistance and increasing blood flow.^29^ More recently, Healey theorized that metastases occurred more often in the dominant hand, due to greater blood supply than the nondominant counterpart.^14^ These theories of traumatized digits and the dominant hand having increased risk for acrometastasis have been propagated despite lack of further evidence in the literature.

We hypothesize that trauma brings the lesion to clinical attention, rather than contributing to susceptibility of developing a metastatic lesion. Trauma to the hand or wrist before presentation was only described in 9.3% of cases. In those, insignificant trauma was the most commonly (38.2%) described injury. These cases included patients who described injuring their digit by “pulling weeds,”^30^ “Lifting a bucket,”^31^ “pricked by a thorne,”^32^ “embedded steel wool while doing dishes,”^33^ and “placing hand in pocket.”^34^ Our hypothesis is supported by cases such as the one presented by Hsieh et al, who describe a patient with known cancer who underwent bone scintigraphy before surgery to rule out metastasis. The scan demonstrated increased uptake of the thumb, which was declared to be artifact. Three months later, the patient fell and presented to the emergency department with a pathological fracture from an esophageal metastatic lesion.^35^

Furthermore, the purported relationship between the location of acrometastasis and hand dominance is not supported by the literature. Surprisingly, few articles specified hand dominance, and of these, just more than one-third of patients had metastasis in their dominant hand. Furthermore, the rate of lesions to the right or left hand or wrist within the total patient population was near equivalent. If there were indeed a correlation, one would suspect to see a greater proportion of right-handed metastasis, given that right-handedness is more commonplace in the general population.

X-ray imaging remains a preliminary investigation of the hands and wrist, with osteolytic destruction being the classic presentation. This is hypothesized to be secondary to stimulation of osteoclast cells by malignant cell activity.^36^ Bone scans were the second most common imaging modality apart from x-rays. They remain an important tool in identifying areas of metastatic involvement or occult malignancies.^37^ Magnetic resonance imaging and PET scans were used infrequently but better define bone and soft tissue lesions and identify clusters of malignant cells.^38,39^ Of those with soft tissue lesions, 44.0% did not report any imaging, despite its role in ruling out bony involvement. In fact, of the patients with soft tissue lesions who did have imaging performed, half were found to have bony involvement. Biopsy was used in most cases and can provide definitive diagnosis and prevent treatment delays.

Acrometastasis remains a grave diagnosis as it often indicates widespread disease. It is encouraging that, on comparison of cases published before and after the year 2000, we have seen an increase in mean survival from 5.4 to 7.4 months. Given that the diversity of cancers presenting as acrometastasis has not changed, this increase in reported survival might suggest that advances in diagnostics and/or treatments are contributory. An independent factor of survival previously demonstrated in renal cell carcinomas is latency between primary malignancy diagnosis and acrometastasis development.^40^ This may demonstrate a more indolent cancer, or a higher patient resiliency as it was comparable across all cancer groups.

The mainstay of treatment for acrometastasis remains amputation most commonly proximal to the nearest uninvolved joint. Practitioners should balance the need for adequate resection to reduce risk of local recurrence with the goal of maximizing function. Radiotherapy or symptom management alone was used more commonly as the metastatic lesion became more proximal. In fact, with metastatic lesions involving the carpal bones, radiotherapy alone became more common than amputation alone, likely to preserve function and avoid invasive management. In addition, the percentage of patients electing for palliative or comfort care increased as the lesion became more proximal. There was no significant difference in survival when comparing patients who underwent radiotherapy alone versus amputation alone for lesions in similar locations. As immunotherapy and chemotherapeutic advancements are made, treatment of acrometastasis should continue to be an adjunct to systemic therapy directed by the primary cancer with the goal of improving patient quality of life. Owing to the rarity of acrometastasis, there are no standardized guidelines or treatment protocols. Given our extensive review of the literature, we provide recommendations below (Table 3).

**Table 3.** Treatment Recommendations for Patients With Acrometastasis to the Hand and Wrist.

Recommendation number	Description of recommendation
Recommendation no. 1	Patients with a history of cancer, particularly of the lung, kidney, breast, colon, or esophagus presenting with a new painful digit, should undergo initial imaging with an x-ray ± a biopsy to rule out acrometastasis.
Recommendation no. 2	If infection is suspected in a patient with a history of cancer, particularly those listed above, consider obtaining a biopsy of the tissue you culture.
Recommendation no. 3	Acrometastasis should be on the differential diagnosis for patients diagnosed with infection who are unresponsive to treatment, particularly if the patient has significant risk factors for malignancy.
Recommendation no. 4	Patients with risk factors for lung cancer should be screened for lung cancer if they present with a finger lesion suspicious for metastasis.
Recommendation no. 5	Treatment of acrometastasis should focus on symptom relief and function. Treatment should serve as an adjunct to systematic therapy directed by the primary malignancy and local tumor board.
Recommendation no. 6	Amputation should be performed to the nearest joint or to minimize tissue loss. There has been no superiority shown with further aggressive amputation.
Recommendation no. 7	Counsel patients to the grim prognosis associated with acrometastasis.

This exhaustive review has demonstrated that acrometastasis remains an uncommon presentation of metastatic disease with poor prognosis. Treatment currently focuses on pain management and optimizing functional outcomes, with amputation and radiotherapy being the most common modalities. Analysis of our findings in this review led to the development of 7 clinical recommendations that may inform clinical practice.

## Supplemental Material

sj-docx-1-han-10.1177_15589447231153175 – Supplemental material for Cancer Metastases to the Hand: A Systematic Review and Meta-analysis Supplemental material, sj-docx-1-han-10.1177_15589447231153175 for Cancer Metastases to the Hand: A Systematic Review and Meta-analysis by Todd Dow, Caitlin Davis, Rawan ElAbd, Donald Lalonde and Jason Williams in HAND

sj-docx-2-han-10.1177_15589447231153175 – Supplemental material for Cancer Metastases to the Hand: A Systematic Review and Meta-analysis Supplemental material, sj-docx-2-han-10.1177_15589447231153175 for Cancer Metastases to the Hand: A Systematic Review and Meta-analysis by Todd Dow, Caitlin Davis, Rawan ElAbd, Donald Lalonde and Jason Williams in HAND

sj-docx-3-han-10.1177_15589447231153175 – Supplemental material for Cancer Metastases to the Hand: A Systematic Review and Meta-analysis Supplemental material, sj-docx-3-han-10.1177_15589447231153175 for Cancer Metastases to the Hand: A Systematic Review and Meta-analysis by Todd Dow, Caitlin Davis, Rawan ElAbd, Donald Lalonde and Jason Williams in HAND
