# Supplementary material for: Cancer Metastases to the Hand: A Systematic Review and Meta-analysis
Source: Hand (N Y). 2023 Mar 1;19(6):865–74. doi: 10.1177/15589447231153175 (PMC11342693; doi:10.1177/15589447231153175)
Supplement: sj-docx-1-han-10.1177_15589447231153175 – Supplemental material for Cancer Metastases to the Hand: A Systematic Review and Meta-analysis [file sj-docx-1-han-10.1177_15589447231153175.docx]

**APPENDIX A: SEARCH STRATEGY**

**Medline Search Criteria:**

| 1 | Acrometast*.mp. | 138 |
| --- | --- | --- |
| 2 | Finger metast*.mp. | 9 |
| 3 | Hand metast*.mp. | 71 |
| 4 | Carpal metast*.mp. | 4 |
| 5 | Extrem* metast*.mp. | 67 |
| 6 | Digit metast*.mp. | 3 |
| 7 | Small bone metast*.mp. | 8 |
| 8 | Wrist metast*.mp. | 5 |
| 9 | 1 or 2 or 3 or 4 or 5 or 6 or 7 or 8 or 9 | 282 |
| 10 | Phalan*.mp. | 12381 |
| 11 | Metacarpal.mp. | 7630 |
| 12 | Scaphoid.mp. | 5489 |
| 13 | Trapezium.mp. | 1424 |
| 14 | Trapezoid.mp. | 2217 |
| 15 | Hamate.mp. | 1029 |
| 16 | Triquetrum.mp. | 580 |
| 17 | Lunate.mp. | 3169 |
| 18 | Pisiform.mp. | 588 |
| 19 | Capitate.mp. | 1264 |
| 20 | 10 or 11 or 12 or 13 or 14 or 15 or 16 or 17 or18 or 19 | 29607 |
| 21 | Metast*.mp. | 635414 |
| 22 | 20 and 21 | 428 |
| 23 | 9 or 22 | 649 |

**Results:** 649 articles

**Cochran Database Search Criteria:**

**Results:** 0 articles

**Pubmed Search Criteria:**

(metastasis) AND (Finger OR acrometast* OR hand OR Carpal OR digit OR Wrist OR Phalan* OR metacarpal OR scaphoid OR trapezium OR trapezoid OR hamate OR triquetrum OR lunate OR pisiform OR capitate)

**Results**: 8041 articles

**Embase Search Criteria using PICO tool:**

('hand'/exp OR 'hand' OR 'hand position' OR 'left hand' OR 'finger'/exp OR 'digit of the hand' OR 'digitus manus' OR 'finger' OR 'finger movement' OR 'finger size' OR 'finger thickness' OR 'fingers' OR 'hand digit' OR 'metacarpal bone'/exp OR 'metacarpal bone' OR 'metacarpal bones' OR 'metacarpus' OR 'digit body part'/exp OR 'phalanx'/exp OR 'phalangeal bone' OR 'phalangeal epiphyse' OR 'phalangization' OR 'phalanx' OR 'carpal bone'/exp OR 'carpal bone' OR 'carpal bones' OR 'carpus bone' OR 'os carpale' OR 'ossa carpalia' OR 'wrist'/exp OR 'articulatio radiocarpea' OR 'carpus' OR 'joint, wrist' OR 'radio-carpal joint' OR 'radiocarpal joint' OR 'wrist' OR 'wrist joint' OR 'small bones' OR 'scaphoid bone'/exp OR 'carpal navicular bone' OR 'carpal scaphoid bone' OR 'hand navicular bone' OR 'navicular bone, hand' OR 'os naviculare manus' OR 'scaphoid bone' OR 'trapezium bone'/exp OR 'bone, trapezium' OR 'os multangulum majus' OR 'os trapezium' OR 'trapezium bone' OR 'trapezoid bone'/exp OR 'bone, trapezoid' OR 'os multangulum minus' OR 'os trapezium minor' OR 'os trapezoideum' OR 'trapezoid bone' OR 'hamate bone'/exp OR 'bone, hamate' OR 'hamate bone' OR 'hamatum bone' OR 'os hamatum' OR 'triquetrum bone'/exp OR 'os triquetrum' OR 'pyramidal bone' OR 'triquetial bone' OR 'triquetral bone' OR 'triquetrial bone' OR 'triquetrum' OR 'triquetrum bone' OR 'lunate bone'/exp OR 'carpal lunate' OR 'carpal lunate bone' OR 'carpal semilunar bone' OR 'lunar bone' OR 'lunate' OR 'lunate bone' OR 'lunatum bone' OR 'os lunatum' OR 'semilunar bone' OR 'pisiform bone'/exp OR 'os pisiforme' OR 'pisiform bone' OR 'capitate bone'/exp OR 'capitate bone' OR 'os capitatum') AND ('acrometastasis'/exp OR acrometastases OR 'hand metastasis'/exp OR 'hand metastases' OR 'extremity metastasis' OR 'extremity metastases' OR 'small bone metastasis' OR 'small bone metastases' OR 'carpal metastasis' OR 'carpal metastases' OR 'finger metastasis'/exp OR 'finger metastases' OR 'wrist metastasis' OR 'wrist metastases')

**Results: 121**

Search performed January 7^th^, 2022
