# Supplementary material for: Cancer Metastases to the Hand: A Systematic Review and Meta-analysis
Source: Hand (N Y). 2023 Mar 1;19(6):865–74. doi: 10.1177/15589447231153175 (PMC11342693; doi:10.1177/15589447231153175)
Supplement: sj-docx-2-han-10.1177_15589447231153175 – Supplemental material for Cancer Metastases to the Hand: A Systematic Review and Meta-analysis [file sj-docx-2-han-10.1177_15589447231153175.docx]

APPENDIX B: REVIEWED ARTICLES

1. Komminoth J. Métastase cutanée digitale d'un cancer du plancher buccal. Annales doto-laryngologie et de chirurgie cervico faciale, bulletin de la Socit doto-laryngologie des hpitaux de Paris. 1977;94(1-2):53-56.
2. Isomura ET, Hamaguchi M, Nishimura N, Ushimura A, Namikawa M. Metastasis to the finger of oral floor squamous cell carcinoma: A case report. Clinical Case Reports. 2021 Jan;9(1):8-11.
3. Mohanty SN, Samanta DR, Avinash A, Senapati SN. Acral metastasis in carcinoma of buccal mucosa: an unusual presentation. Oncology Journal of India. 2018 Apr 1;2(2):35.
4. Fragiadakis EG, Panayotopoulos G. Metastatic carcinoma of the hand. The Hand. 1972 Jan 1;4(3):268-72.
5. Castello JR, Garro L, Romero F, Campo M, Najera A. Metastatic tumours of the hand: report of six additional cases. Journal of Hand Surgery. 1996 Aug;21(4):547-50.
6. Viswanathan PN, Rangad F, Roul RK. Metastases to the hand from carcinoma of the lower alveolus. Journal of Hand Surgery. 1996 Aug;21(4):544-6.
7. Shrivastava R, Singh KK, Umbarker BR, Karle R, Shrivastava M. Phalange metastasis from carcinoma of alveolus. Indian Journal of Dental Research. 2009 Oct 1;20(4):496-498.
8. Sandberg Y, van de Wiel BA, van Meerten E. Digital Acrometastasis. Arthritis & Rheumatology. 2015 May;67(5):1410-.
9. Trehan R, Pandey AK, Khosla D, Dimri K, Punia RS. Apropos of a case of cutaneous metastasis from laryngeal cancer with review of literature. Journal of cancer research and therapeutics. 2015 Jul 1;11(3):655.
10. Castigliano SG. Metastasis to the distal phalanx from a primary malignancy of the oral cavity: report of case. Journal of oral surgery (American Dental Association: 1965). 1966 Sep;24(5):467-9.
11. Vanel D, Luboinski B, Micheau C, Wibault P, Masselot J. Phalangette metastases of cancer of the mouth. An uncommon mode of propagation. Journal de radiologie. 1990 Mar 1;71(3):237-44.
12. Longo R, Torino F, Castellana M, Amici S, Verì A, Cacciamani F, Gasparini G, Lanzi G. Skin acrometastases in squamous cell carcinoma of the tongue. American Society of Clinical Oncology. Journal of Clinical Oncology. 2007;25(19);2847-2848
13. Aiempanakit K. Digital metastasis of tongue squamous cell carcinoma. JAAD case reports. 2018 Mar;4(2):200.
14. Mandadi SR, Kudva A. Acrocutaneous metastasis–A rare presentation. International journal of surgery case reports. 2018 Jan 1;51:178-80.
15. Joll CA. Metastatic tumors of bone. Br J Surg 1923;11:38–72.
16. Plotkine E, Coscas R, Guigui P. Digital metastatis of a nasopharyngeal carcinoma: A surgical trap: Case report and literature review. Chirurgie de la main. 2008 Sep 1;27(4):187-90.
17. TaesooChung. Metastatic malignancy to the bones of the hand. Journal of surgical oncology. 1983 Oct;24(2):99-102.
18. Sur YJ, Kang YK, Bahk WJ, Chang DK, Rhee SK. Metastatic malignant tumour in the hand. Journal of plastic surgery and hand surgery. 2011 Apr 1;45(2):90-5.
19. Gold GL, Reefe WE. Carcinoma and metastases to the bones of the hand. JAMA. 1963 Apr 20;184(3):237-9.
20. Sipahioglu S, Zehir S, Ozkanli U. Nasopharyngeal carcinoma with hand metastasis. Journal of hand surgery. European volume. 2012;37(6):578-9.
21. Filloux JF, Fontaine C. Soft tissue metastatic tumors of the fingers. Six cases. Chirurgie de la Main. 2000 Feb 1;19(1):63-6.
22. Pirschel J, Metzger HO, Wissmann C. Zur Metastasierung maligner Tumoren in die Skelettperipherie. InRöFo-Fortschritte auf dem Gebiet der Röntgenstrahlen und der bildgebenden Verfahren 1978 Nov (Vol. 129, No. 11, pp. 621-626). © Georg Thieme Verlag KG Stuttgart· New York.
23. Bazex A, Salvador R, Dupre A, Cantala P. Métastases symétriques des extrémités des annulaires secondaires à un cancer du larynx. Bull Soc Grancaise Dermatol Syphil. 1968;75:131-3.
24. Cohen HJ, Laszlo J. Influence of trauma on the unusual distribution of metastases from carcinoma of the larynx. Cancer. 1972 Feb;29(2):466-71.
25. Uriburu IJ, Morchio FJ, Marin JC. Metastases of carcinoma of the larynx and thyroid gland to the phalanges of the hand. Report of two cases. JBJS. 1976 Jan 1;58(1):134-6.
26. Mess D, Songer M. Head and neck carcinoma metastases to the hand and foot: a case report of simultaneous involvement. Orthopedics. 1986 Jul 1;9(7):975-7.
27. Lewin JS, Cleary KR, Eicher SA. An unusual metastasis to the thumb in a laryngectomized tracheoesophageal speaker. Archives of Otolaryngology–Head & Neck Surgery. 1997 Sep 1;123(9):1007-9.
28. Aydin O, Ustündağ E, Boyaci Z, Müezzinoğlu B. Skin metastasis: an unusual localization from laryngeal carcinoma. Kulak burun bogaz ihtisas dergisi: KBB= Journal of ear, nose, and throat. 2004 Mar 1;12(3-4):103-6.
29. Ottomani S, Levadoux M, Cathelineau O, Nguyen M, Monchal T. Double simultaneous fingertip metastasis of an epidermoid carcinoma. A case report. Chirurgie de la Main. 2008 Sep 4;27(5):243-5.
30. Kumar N, Kumar R, Bera A, Kumar P, Angurana SL, Ghosal S, Srinivasan R, Sharma SC. Palliative and supportive care in acrometastasis to the hand: case series. Indian journal of palliative care. 2011 Sep;17(3):241-244.
31. Shetty N, Sameer MS, Fernandes H, Shet D. Metastases of head and neck squamous cell carcinoma to soft tissue of fingertips. Indian journal of medical and paediatric oncology: official journal of Indian Society of Medical & Paediatric Oncology. 2016 Apr;37(2):122.
32. Elvey MH, Aghasi M, Wasrbrout Z, Avisar E. Metastasis of parotid basal cell adenocarcinoma to the hand—a case report. Hand. 2011 Sep;6(3):321-3.
33. Esther RJ, Bos GD. Management of metastatic disease of other bones. Orthopedic Clinics of North America. 2000 Oct 1;31(4):647-59.
34. Mason ML. Tumors of hands. Surg. Gynecol. Obstet.. 1937;64:129-48.
35. Ariel IM, Jerome AP, Pack GT. Treatment of tumors of the parotid salivary gland. Surgery. 1954 Jan 1;35(1):124-58.
36. Falkinburg LW, Fagan JH. Malignant mixed tumor of the parotid gland with a rare metastasis. The American Journal of Surgery. 1956 Feb 1;91(2):279-82.
37. Vinod SU, Gay RM. Adenoid cystic carcinoma of the minor salivary glands metastatic to the hand. Southern medical journal. 1979 Nov 1;72(11):1483-5.
38. Horn Y. Metastatic iodophilic carcinoma of thyroid to a hand bone. Journal of surgical oncology. 1982 Mar;19(3):123-6.
39. Reparaz Padros J, Arenas Planelles AJ, Martinez Mariscal J, Garbayo Marturet AJ. Acrometástasis. A propósito de dos Casos. Rev Esp Cir Osteoarticular. 2006;41(225):39-43.
40. Huri G. An atypical metastasis of follicular-type adenocarcinoma of the thyroid gland to thumb. Case reports in orthopedics. 2011 Dec 29;2011.
41. Krishnamurthy A, Ramshankar V. Metastatic iodophilic follicular carcinoma of thyroid to a hand bone. Thyroid Research and Practice. 2013 Jan 1;10(1):36.
42. Kattepur AK, Gopinath KS. Metastasis from thyroid carcinoma. N Engl J Med. 2014 May 29;370:2131.
43. Patil D, Kattepur AK, Gopinath SK, Swamy S, Shankarappa A, Srinivasachar GK. Iodophilic synchronous phalangeal and choroidal metastasis from follicular thyroid carcinoma: a case report and review. European thyroid journal. 2014;3(2):134-6.
44. Chakravarthy VK, Rao ND, Chandra ST. Study of papillary carcinoma of thyroid with uncommon sites of metastasis. Indian Journal of Otolaryngology and Head & Neck Surgery. 2010 Jun;62(2):198-201.
45. Shannon FJ, Antonescu CR, Athanasian EA. Metastatic thymic carcinoma in a digit: a case report. The Journal of hand surgery. 2000 Nov 1;25(6):1169-72.
46. Rendich RA, Levy AH. Unusual metastatic bone lesions. Am J Roentgenol Radium Ther Nucl Med. 1941;46:343-50.
47. Greene MH. Metastasis of pulmonary carcinoma to the phalanges of the hand. JBJS. 1957 Jul 1;39(4):972-5.
48. Smith RJ. Involvement of the carpal bones with metastatic tumor. The American journal of roentgenology, radium therapy, and nuclear medicine. 1963 Jun;89:1253-5.
49. Hicks MC, Kalmon Jr EH, Glasser SM. Metastatic malignancy to phalanges. Southern medical journal. 1964 Jan;57:85-8.
50. Mulvey RB. Peripheral bone metastases. The American journal of roentgenology, radium therapy, and nuclear medicine. 1964 Jan;91:155-60.
51. Taddei L, Pistocchi F. Phalangeal carcinomatous metastases. La Radiologia medica. 1965 Feb;51:145-50.
52. Mohanty S, Federowicz TE, Uehara H. Metastatic lesions of the fingers. Surgery. 1968;64(2):411-5.
53. Tondeur G, Verschraegen J, Depauw A, Leempoel A. Tumeur metastatique de la main. A propos d'une observation. Acta chir belg. 1969;68(2):141-6.
54. Fam AG, Cross EG. Hypertrophic osteoarthropathy, phalangeal and synovial metastases associated with bronchogenic carcinoma. The Journal of rheumatology. 1979 Nov 1;6(6):680-6.
55. Saitoh Y, Hirata K, Katoh T, Sugawara M, Miura S, Abo S, Kudo T, Watanuki T. A case of lung cancer metastasis to the hand. Rinsho hoshasen. Clinical radiography. 1983 Apr 1;28(4):483-6.
56. Rochet N, Pages M, Lassoued S, Poey C, Fournié B, Fournié A. Phalangeal metastasis of the hand. Apropos of a case. Revue du rhumatisme et des maladies osteo-articulaires. 1991 Jan;58(1):73-4.
57. Rousseau A, Madinier JF, Favre A, Michenet P. Metastatic tumors of the soft tissues of the hand. Apropos of a case. Review of the literature. Annales de chirurgie de la main et du membre superieur: organe officiel des societes de chirurgie de la main= Annals of hand and upper limb surgery. 1992 Jan 1;11(1):57-61.
58. Roncaglio C, Arena B. Metastasi alla mano da carcinoma broncopolmonare. Minerva Ortop Traumatol. 1993;44(1–2):37-43.
59. Knapp D, Abdul-Karim FW. Fine needle aspiration cytology of acrometastasis. A report of two cases. Acta cytologica. 1994 Jul 1;38(4):589-91.
60. Abrahams TG. Occult malignancy presenting as metastatic disease to the hand and wrist. Skeletal radiology. 1995 Feb;24(2):135-7.
61. Javed S, Fenyar B, Cilursu AM. Finger metastasis mimicking osteomyelitis case report and review of literature. Journal of clinical rheumatology: practical reports on rheumatic & musculoskeletal diseases. 1997 Jun 1;3(3):168-70.
62. Galmarini CM, Kertesz A, Oliva R, Porta J, Galmarini FC. Metastasis of bronchogenic carcinoma to the thumb. Medical oncology. 1998 Dec;15(4):282-5.
63. Matsuno Y, Watanabe K, Satoh H, Yamashita YT, Sekizawa K. Thenar metastasis from lung cancer. European journal of cancer care. 2002 Mar;11(1):61-2.
64. Raissouni Z, Ismael F, Elmrini A, Boutayeb F. Acrometastasis of the hand. Case report. Acta orthopaedica Belgica. 2002 Jun 1;68(3):297-300.
65. Afifi AM, Perez A. Finger metastasis from pulmonary carcinoma. A case report. REVUE DE GERIATRIE. 2004;29(4):267-72.
66. Campa T, Fagnoni E, Ripamonti C. Palliative surgery of acrometastases from lung cancer: a case report. Supportive care in cancer. 2004 Mar;12(3):202-4.
67. Keramidas E, Brotherston M. Extensive metastasis to the hand from undiagnosed adenocarcinoma of the lung. Scandinavian journal of plastic and reconstructive surgery and hand surgery. 2005 Apr 1;39(2):113-5.
68. Akjouj S, El Kettani N, Semlali S, Chaguar B, Chaouir S, Hanine A, Benameur M. Thumb acrometastasis revealing lung adenocarcinoma: a case report with review of literature. Chirurgie de la Main. 2006 Jun 1;25(2):106-8.
69. Han JQ, Han CY, Bi YH. Clinical features of patients with metastasis in phalanges as first symptom of primary lung cancer. Chinese Journal of Oncology. 2007 Jul 1;29(7):534.
70. Preto AS, Teixeira R, Cruz R. Atypical presentation of lung cancer [in Portuguese]. Acta Reumatol Port. 2007;32(3):282-286.
71. Ahlmann ER, Greene NW, Menendez LR, Stevanovic MV. Unusual locations for metastatic malignancy of the hand: a report of three cases. Journal of surgical orthopaedic advances. 2008 Jan 1;17(4):267-70.
72. Seth R, Athanassopoulos A, Mir S. First presentation of lung adenocarcinoma as a subungual metastasis. Hand. 2008 Mar;3(1):69-71.
73. Jakhar SL, Dana R, Punia DP. Cancer lung: An unusual presentation. Indian journal of medical and paediatric oncology: official journal of Indian Society of Medical & Paediatric Oncology. 2009 Oct;30(4):149-150.
74. Kodama T, Kikuchi N, Satoh H, Ohtsuka M. Metacarpal bone metastasis from lung cancer. Oncology Research and Treatment. 2009;32(4):216-7.
75. Myrehaug S, Bezjak A. Rapidly progressive bone destruction of the finger as first presentation of systemic metastases from lung cancer. Case Reports. 2010 Jan 1;2010:bcr0520091912.
76. Long LS, Brickner L, Helfend L, Wong T, Kubota D. Lung cancer presenting as acrometastasis to the finger: a case report. Case reports in medicine. 2010 Jun 13;2010.
77. Stathopoulos GP, Rigatos SK. Rare Site of Metastasis of Non-Small-Cell Lung Cancer. Journal of BU ON.: official journal of the Balkan Union of Oncology. 2010;15(1):189-90.
78. Amar MF, Almoubaker S, Lahrach K, Chbani B, Bennani A, Marzouki A, Boutayeb F. A giant tumor of the thumb revealing bronchic adenocarcinoma (a case report). Chirurgie de la Main. 2011 Feb 1;30(2):133-5.
79. Bhandari T, Brown E. Acrometastasis and the potential benefits of early positron emission tomography scanning. Annals of plastic surgery. 2011 Aug 1;67(2):189-92.
80. Huang FX, Zhang N, Liu L, Zhang H. Metastasis of lung adenocarcinoma to the fifth distal phalange of right hand. Case Reports. 2011 Sep 30;2011:bcr0520114276.
81. Sasaki Y, Minamiya Y, Okuyama M, Hibino M, Kudo S, Tenma K, Nakamura M, Ogawa J. A case of lung cancer with skin metastases responding to erlotinib. Gan to kagaku ryoho. Cancer & chemotherapy. 2011 Feb 1;38(2):271-4.
82. van Uden DJ, Kolkman K. A man with a painful finger. Nederlands tijdschrift voor geneeskunde. 2011 Jan 1;155(26):A2228-.
83. Ferraro D, Lucero P. Metastatic lung adenocarcinoma presenting as thumb pain. Chest. 2012 Oct 1;142(4):567A.
84. Gilardi R, Rosa ND, Pancaldi G, Landi A. Acrometastasis showing an occult lung cancer. Journal of plastic surgery and hand surgery. 2013 Dec 1;47(6):550-2.
85. Khmamouche MR, Aassab R, Debbagh A, et al. A thumb acrometastasis revealing lung adenocarcinoma: a case report and review. J Clin Case Rep. 2013;3:11000241.
86. Shaw KA, Balog TP, Grassbaugh JA. Acrometastasis of the Thumb. Orthopedics. 2013;36(12):903-66.
87. Liu WC, Ho CJ, Lu CK, Wu CC, Fu YC, Chien SH. Acrometastasis to metacarpal bone disclosing an occult lung cancer. Case Reports in Plastic Surgery and Hand Surgery. 2014 Jan 1;1(1):23-5.
88. Poh ME, Liam CK, Tan JL, Pang YK, Wong CK, Kow KS. Acrometastasis from an epidermal-growth-factor-receptor (EGFR) mutation-positive lung adenocarcinoma. Cancer Treatment Communications. 2014 Jan 1;2(2-3):21-3.
89. Sumodhee S, Huchot E, Peret G, Marchal C, Paganin F, Magnin V. Radiotherapy for a phalanx bone metastasis of a lung adenocarcinoma. Case Rep Oncol. 2014;7(3):727-731. doi:10.1159/000368345
90. van Veenendaal LM, de Klerk G, Velde DV. A painful finger as first sign of a malignancy. Geriatric orthopaedic surgery & rehabilitation. 2014 Mar;5(1):18-20.
91. Gorospe Sarasúa LG, Pérez LM, Blanco RE. Acrometastasis as the initial presentation of lung adenocarcinoma in a young woman. Archivos de bronconeumologia. 2016 Sep;52(9):482-3.
92. Reynolds J, Skandan SP. An uncommon presentation of non-small-cell lung cancer with acrometastases to the great toe and index finger. The Journal of community and supportive oncology. 2016 Mar 1;14(3):122-5.
93. Feng J, Song QB, Peng M. Two cases of lung cancer with bone metastasis of hand. Zhonghua zhong liu za zhi [Chinese journal of oncology]. 2017 Jun 1;39(6):477-8.
94. Xie P, Huang J. Solitary thumb acrometastasis identified on bone scintigraphy. Clinical nuclear medicine. 2017 Jul 1;42(7):549-50.
95. El Idrissi M, Akasbi N, Elibrahimi A, Elmrini A. Acrometastasis as the first manifestation of lung carcinoma. Eur J Rheumatol. 2018;5(3):212-213.
96. Millrose M, Buehren V, Heidenreich C. Ossäre Metastase eines Adenokarzinom an einer Fingerphalanx. Handchirurgie· Mikrochirurgie· Plastische Chirurgie. 2018 Jun;50(03):214-5.
97. Afrăsânie VA, Adavidoaiei AM, Zamisnicu IH, Funingănă IG, Marinca MV, Gafton B, Clement DE, Păduraru MI, Demşa I, Miron L, Alexa-Stratulat T. A very rare presentation of lung cancer: Metastases to the distal phalanx of index–case report. Medicine. 2019 Dec;98(49).
98. Machado V, San-Julian M. Prognosis and treatment of acrometastases: Observational study of 35 cases treated in a single institution. Revista Española de Cirugía Ortopédica y Traumatología (English Edition). 2019 Jan 1;63(1):49-55.
99. Clery E, Pisapia P, Migliatico I, Pepe F, De Luca C, Russo M, De Rosa F, Smeraglia F, Insabato L, Vigliar E, Malapelle U. Cytology meets next generation sequencing and liquid biopsy: A case of lung adenocarcinoma presenting as metastasis to the phalanx. Diagnostic cytopathology. 2020 Aug;48(8):759-64.
100. Kalakech S, El Abiad R, Kesrouani C, Kattan J, Nassereddine H. Acrometastasis revealing a pulmonary adenocarcinoma: Report of a case with unusual histopathological findings. InAnnales de Pathologie 2021 Apr 2.
101. Umana GE, Scalia G, Palmisciano P, Passanisi M, Pompili G, Amico P, Ippolito M, Sabini MG, Cicero S, Perrotta R. Spontaneous sacral fracture with associated acrometastasis of the hand. Surgical Neurology International. 2021;12.
102. Ross EF, Fodden JH. Metastasis of Bronchogenic Carcinoma. Canadian Medical Association journal. 1949;61(3):310.
103. Midell AI, Lochman DJ. An unusual metastatic manifestation of a primary bronchogenic carcinoma. Cancer. 1972 Sep;30(3):806-9.
104. Nissenbaum MA, Kutz JE, Lister GD. Clear-cell carcinoma of the lung metastatic to the hamate: a case report. Clinical orthopaedics and related research. 1978 Jul 1(134):293-6.
105. Letanche G, Dumontet C, Euvrard P, Souquet PJ, Bernard JP. Distal metastases of bronchial cancers. Bone and soft tissue metastases. Bulletin du cancer. 1990 Jan 1;77(10):1025-30.
106. De Abaffy AM, Richter RH, Grünert J. Peripheral bone metastasis of a rare lung cancer. Archives of orthopaedic and trauma surgery. 1998 Oct 1;117(8):477-8.
107. Rinonapoli G, Caraffa A, Antenucci R. Lung cancer presenting as a metastasis to the carpal bones: a case report. Journal of medical case reports. 2012 Dec;6(1):1-6.
108. Koyama M, Koizumi M. FDG-PET images of acrometastases. Clinical nuclear medicine. 2014 Mar 1;39(3):298-300.
109. Strooker JA, Maas M, Bulkmans N, Kreulen M. Painful metastasis in the lunate bone as the first symptom of a Pancoast tumor; a case report and review of the literature. Journal of hand and microsurgery. 2015 Dec;7(2):343-5.
110. Berthier C, Mhanna L, Lupon E. Surgical treatment of a digital metastasis of a large cell lung neuroendocrine carcinoma: A rare secondary anatomical localization. InAnnales de chirurgie plastique et esthetique 2020 Feb (Vol. 65, No. 1, pp. 87-90).
111. Graham RM, Sharp MC, Ashcroft GP. An unusual case of finger swelling: A case report. Cases journal. 2009 Dec;2(1):1-2.
112. Bonvoisin B, Joasson JM, Bouvier M, Bejui-Thivolet F, Galy P. Distal bone metastasis (index finger) with a pseudophlegmonous appearance, of bronchial origin. La Nouvelle presse medicale. 1981 May 2;10(20):1656-7.
113. Asencio G, Hafdi C, Pujol H, Allieu Y. Osseous metastases in the hand. A general review of three cases. Annales de chirurgie de la main: organe officiel des societes de chirurgie de la main. 1982 Jan 1;1(2):137-45.
114. Dyck P. Unusual metastasis of a pleural mesothelioma.(Report on an observed case). Zentralblatt fur Chirurgie. 1965 Jun 1;90(23):903-6.
115. Kanbay A, Oguzulgen KI, Ozturk C, Memis L, Demircan S, Kurkcuoglu C, Akyurek N, Kurul C. Malignant pleural mesothelioma with scalp, cerebellar, and finger metastases: a rare case. Southern medical journal. 2007 Jan 1;100(1):63-6.
116. Celik G, Saryal S, Enacar N. Mixed squamous and small cell lung carcinoma with separate histologic subtypes in eye and phalanx metastases. Journal of experimental & clinical cancer research: CR. 1998 Mar 1;17(1):129-30.
117. Floridis GG, Nixon CE, Ginsburg HM. Primary carcinoma of the lung with unusual manifestations. California and western medicine. 1934 May;40(5):365.
118. Brahdy L, Kahn S. Trauma and Disease. 2nd ed. Philadelphia: Lea and Febiger; 1941:471.
119. Brailsford JF. The radiology of bones and joints. J. & A. Churchill; 1953. 99-100
120. Reboul J, Delorme G, Bobo J, Sorin Y. Etude radioclinque du cancer secondaire des os. Ann Radiol. 1960;3:727-51.
121. Vancura J, Jakoubková J, Kolár J. Metastases in Small Bones of the Hand in Pulmonary Cancer. Zentralblatt fur Chirurgie. 1960 Jul 16;85:1554-7.
122. Amalric R, Clement R, Roux G, Lesto J. De la frequence des metastases osseuses des carcnomes bronchiques. In Journal de radiologie d electrologie et de medicine nucleaire. 1966;47(11):711
123. Patryn A. A case of metastasis of bronchial cancer to the hand recognized and treated as tuberculosis of the wrist. Polski tygodnik lekarski (Warsaw, Poland: 1960). 1967 Nov 13;22(46):1777-8.
124. Dolich BH, Spinner M, Kaufman G. Isolated metastasis to the carpal bones. Report of a case. Bulletin of the Hospital for Joint Diseases. 1970 Apr 1;31(1):78-84.
125. Hammer B, Gollmann G. Phalangenmetastasen bei bronchuskarzinom: beltrag zur uberlebenszeit. Muenchen Med Wochenschr. 1972;114:61-3.
126. Rolle J, Berner H. Isolierte metastase eines bronchialcarcinoms im bereich des rechten daumens. Handchirurgie. 1974;6:148.
127. Pantoja E, Cross VF, Vitale P, Wendth AJ. Neoplastic involvement of terminal digits masquerading clinically as benign disease. Revista interamericana de radiologia. 1976 Jul 1;1(1):9-13.
128. Bricout PB. Acrometastases. J Natl Med Assoc 1981;73:325–9.
129. Cary PC, Helms CA, Genant HK. Metastatic disease to the carpus. The British journal of radiology. 1981 Nov;54(647):992-5.
130. Cross AB. Bronchogenic carcinoma presenting as an injured thumb. Emergency Medicine Journal. 1985 Jun 1;2(2):93-6.
131. Morris DM, House HC. The significance of metastasis to the bones and soft tissues of the hand. Journal of surgical oncology. 1985 Feb;28(2):146-50.
132. Lederer A, Flückiger F, Wildling R, Fruhwirth J. A solitary metastasis in the trapezium bone [in German]. Radiologe. 1990;30(2):79-80.
133. Heidarpour M, Rajabi P, Eftekhari A, Ghasemibasir HR. Lung carcinoma metastasis to the distal part of the index finger: a case report. Iran J Pathol. 2006;1(4):173-176.
134. Tzaveas A, Paraskevas G, Pazis I, Dimitriadis A, Kitsoulis P, Vrettakos A. Metastasis of bronchogenic carcinoma to the 5 th metacarpal bone: a case report. Cases Journal. 2008 Dec;1(1):1-4.
135. Bowles F, Wells H. Paronychia: not always what it seems. Emergency Medicine Journal. 2011 Apr 1;28(4):342.
136. Cavit A, Özcanli H, Sançmiş M, Ocak GA, Gürer Eİ. Tumorous conditions of the hand: a retrospective review of 402 cases. Turk Patoloji Derg. 2018 Jan 1;34(1):66-72.
137. Tabrizi A, Afshar A, Shariyate MJ, Gharalari FH, Aidenlou A. Clinical Cases and Observations on Hand Surgery, Reconstruction Procedures, Innovative Ideas, and Emerging Techniques: Part I: Isolated Metastatic Carcinoma to the Hamate Bone: The First Manifestation of an Occult Malignancy. Journal of Hand and Microsurgery. 2019 Oct;11(Suppl 1):S01.
138. Parungao AJ, Milner S. A Swollen, Draining Thumb. American family physician. 2002 Jan 1;65(1):105-106.
139. Alkhayat H, Hong CH. Cutaneous metastases from non-small cell lung cancer.Journal of Cutaneous Medicine and Surgery. 2006;10(6):304-307
140. Bülbül Y, Özsu S, Özlü T, Öztuna F. Acrometastasis in a case with bronchial carcinoma. Toraks Dergisi. 2006;7(3):200-3.
141. Marcos Sánchez, MI AC. Distal phalange metastases as the first sign of an undifferentiated lung carcinoma. InAnales de medicina interna (Madrid, Spain: 1984) 2006 Mar 1 (Vol. 23, No. 3, pp. 147-147).
142. Flynn CJ, Danjoux C, Wong J, Christakis M, Rubenstein J, Yee A, Yip D, Chow E. Two cases of acrometastasis to the hands and review of the literature. Current oncology. 2008 Oct;15(5):51-8.
143. Gaston RG, Lourie GM, Scott CC. Isolated metastatic lesion of the trapezium. Am J Orthop (Belle Mead NJ). 2008 Aug 1;37(8):E144-5.
144. Kontogeorgakos V, Korompilias A, Georgousis M, Papachristou D. Digital metastasis presenting as infection. Journal of hand and microsurgery. 2011 Jun 1;3(1):25-7.
145. Song Y, Yao J. Trapezial Metastasis as the First Indication of Primary Non–small Cell Carcinoma of the Lung. The Journal of hand surgery. 2012 Jun 1;37(6):1242-4.
146. Gjorup AC, Ottesen SS, Gjerloff C. Acrometastasis as the first sign of an occult non-small cell carcinoma of lung. Journal of plastic surgery and hand surgery. 2017 Mar 4;51(2):156-7.
147. Baños-Arévalo AJ, López-Navarro N, Gallego-Domínguez E, Herrera E. Acral metastasis of the fingers: report of 2 cases. Actas Dermo-Sifiliográficas (English Edition). 2018 Dec 1;109(10):e1-4.
148. White RZ, Sampson MJ. Metastatic destruction of the pisiform. Journal of Medical Imaging and Radiation Oncology. 2021:1-3
149. Colson GM, Willcox A. Phalangeal metastases in bronchogenic carcinoma: report of three cases. The Lancet. 1948 Jan 17;251(6490):100-2.
150. Bell JL, Mason ML. Metastatic tumors of the hand; report of two cases. Quarterly Bulletin of the Northwestern University Medical School. 1953;27(2):114.
151. Stock HJ. Zwei seltene knochengeschwulste an der hand. Zentralbl Chir. 1977;102:420-5.
152. Bunkis JU, Mehrhof A, Stayman J. Metastatic lesions of the hand and foot. Orthopedic Review. 1980;9(9):97-10I.
153. Józsa L, Renner A. Tumors and tumor-like lesions arising in tendons. Archives of orthopaedic and trauma surgery. 1991 Feb 1;110(2):83-6.
154. Kolomiets SA, Lytkin AS. Metastasis of small-cell lung cancer to the bone of the finger simulating osseous paronychia. Vestnik khirurgii imeni II Grekova. 1991;147(7-8):55-6.
155. Königsberger H, Goth D. Bone metastasis of bronchial carcinoma to the wrist. Case report. Handchirurgie, Mikrochirurgie, Plastische Chirurgie: Organ der Deutschsprachigen Arbeitsgemeinschaft fur Handchirurgie: Organ der Deutschsprachigen Arbeitsgemeinschaft fur Mikrochirurgie der Peripheren Nerven und Gefasse: Organ der V... 1996 Jan 1;28(1):46-9.
156. Mehta NJ, Mehta RN, Jani K, Nehra A. Subungual and ocular metastases from small cell carcinoma of the lung. Hospital Physician. 2001 Sep;37(9):38-40.
157. Carvalho HD, Tsai PW, Takagaki TY. Thumb metastasis from small cell lung cancer treated with radiation. Revista do Hospital das Clínicas. 2002;57(6):283-6.
158. Anandan SM, Iyer S, Uppal R. Metastases to the finger masquerading as flexor tenosynovitis. Journal of Hand Surgery (European Volume). 2010 Sep;35(7):597-8.
159. Ragois P, Didailler P, Rizzi P. Skin metastasis of lung carcinoma like Dupuytren's disease. Chirurgie de la Main. 2012 Aug 9;31(5):259-61.
160. Hinterstoisser H. Ein Fall von primärem Carcinom der großen Luftwege mit Metastasen an einem Fingerglied. Wien. klin. Wschr. 1889;19:374.
161. Froboese C. Mschr. Unfallkh. 1941:149
162. Smithers DW, Price LW. Isolated secondary deposit in a terminal phalanx in a case of squamous-cell carcinoma of the lung. The British Journal of Radiology. 1945 Sep;18(213):299-300.
163. Preissner T. Schwierigkeiten bei der Diagnose der Bronchialcarcinome, seltene Knochenmetastase. Zbl. Chir. 1948;73:696.
164. Brason FW, Eschner EG, Sanes S, Milkey G. Secondary carcinoma of the phalanges. Radiology. 1951;57(6):864-7.
165. Freni DR, Averill JH. Metastatic carcinoma of the lung to the thumb. The American Journal of Surgery. 1952 Jan 1;83(1):115-6.
166. Strang R. Phalangeal metastases as a first clinical sign of bronchogenic carcinoma. Journal of British Surgery. 1952 Jan;39(156):372-3.
167. De Pass SW, Roswitt B, Unger SM. Metastatic carcinoma in the bones of the hand; a report of two cases. The American journal of roentgenology, radium therapy, and nuclear medicine. 1958 Apr;79(4):643-4.
168. Kerin R. Metastatic tumors of the hand. Plastic and Reconstructive Surgery. 1958 Jul 1;22(1):80.
169. Marmor L, Horner RL. Metastasis to a phalanx simulating infection in a finger. American journal of surgery. 1959 Feb;97(2):236-7.
170. Pfeiffer K. Beitrag zur peripheren skelettmetastasierung. Z Gesamte Inn Med. 1959;14:244-52.
171. Kolář J, Jakoubkova J, Kacl J, Vančura J. Symmetrische knochenmetastasen in den fingern beim lungenkrebs. InRöFo-Fortschritte auf dem Gebiet der Röntgenstrahlen und der bildgebenden Verfahren 1960 May (Vol. 92, No. 05, pp. 588-590). © Georg Thieme Verlag KG Stuttgart· New York.
172. Trachtenberg AS, Roswit B. Bronchogenic carcinoma metastatic to the hand. The American journal of roentgenology, radium therapy, and nuclear medicine. 1961 May;85:886-90.
173. Karten I, Bartfeld H. Bronchogenic Carcinoma Simulating; Early Rheumatoid Arthritis: Metastases To The Finger. JAMA. 1962 Jan 13;179(2):162-4.
174. Ferguson AD, Chall HG, Shapiro RI. Metastatic tumors of the hand. Grace Hosp Bull. 1963;41:20-22.
175. Bailey AH. Case report on unusual metastatic spread of bronchogenic carcinoma. The Journal-lancet. 1964 Dec;84:479-81.
176. Grant KB, Roller GJ. Metastasis of bronchogenic carcinoma to the terminal phalanx of a finger. Journal of the Iowa Medical Society. 1966 Nov;56(11):1132-5.
177. Camiel MR, Aron BS, Alexander LL, Benninghoff DL, Minkowitz S. Metastases to palm, sole, nailbed, nose, face and scalp from unsuspected carcinoma of the lung. Cancer. 1969 Jan;23(1):214-20.
178. Sneddon J. Painless metastatic deposit in a finger presenting as a pulp infection with osteitis. The British journal of clinical practice. 1969 Dec 12;23(12):511-3.
179. Mladenović V, Nikolić J, Kerimović D. Metastasis of bronchial carcinoma in the bones of hands and feet. Reumatizam. 1972;19(4):138-46.
180. Graham WP, Kilgore ES, Williams EH, Gordon SL. Metastatic tumours of the hand. Hand. 1973;5(2):177-9.
181. Singh HK. Unusual metastases in bronchogenic carcinoma. Journal of the Indian Medical Association. 1974 May 16;62(10):351-2.
182. Seyss R. Maligne tumoren im bereich der phalangen. Radiologe. 1975;15(2):69-71.
183. Lombard A, JM DD. Métastase phalangienne simulant une ostéite et révélant un cancer bronchique. Le semaine des hopitaux. 1976;52(2):119-121.
184. La Salle AJ, Andrassy RJ, Stanford W. Bronchogenic squamous cell carcinoma in childhood: a case report. J Pediatr Surg 1977;12:519–21.
185. Patel MR, Silver JW. Metastatic carcinoma of hand; clinical manifestations of occult or overt bronchogenic carcinoma. 1978;78(14):2233-7.
186. Vaezy A, Budson DC. Phalangeal metastases from bronchogenic carcinoma. JAMA. 1978 Jan 16;239(3):226-7.
187. Wu KK, Guise ER. Metastatic tumors of the hand: a report of six cases. The Journal of hand surgery. 1978 May 1;3(3):271-6.
188. Nagendran T, Patel MN, Gaillard WE, Imm F, Walker M. Metastatic bronchogenic carcinoma to the bones of the hand. Cancer. 1980 Feb 15;45(4):824-8.
189. Wu KK, Winkelman NZ, Guise ER. Metastatic bronchogenic carcinoma to the finger simulating acute osteomyelitis. Orthopedics. 1980 Jan 1;3(1):25-8.
190. Drewes J, Sailer R, Schmitt-Graff A. Malignommetastasen der hand. Handchirurgie. 1981;13:296-304.
191. Sarma DP, Socorro N. Metastasis of bronchogenic carcinoma to finger. The Journal of the Louisiana State Medical Society: official organ of the Louisiana State Medical Society. 1981 Mar 1;133(3):37-.
192. Khokhar N, Lee JD. Phalangeal metastasis: first clinical sign of bronchogenic carcinoma. Southern medical journal. 1983 Jul 1;76(7):927-.
193. Martin KA, Dove AF. Metastatic carcinoma of the hand. Hand 1983;15:343–6.
194. Rose BA, Wood FM. Metastatic bronchogenic carcinoma masquerading as a felon. The Journal of hand surgery. 1983 May 1;8(3):325-8.
195. Dubost C, Assens P, Garin G, Charbonnier JY. Métastases osseuses des extrémités: à propos d'une observation de métastase du premier métacarpien avec hypercalcémie. La Semaine des hôpitaux de Paris. 1984;60(24):1699-702.
196. Ioia JV, Sumner JM, Gallagher T. Presentation of malignancy by metastasis to the carpal navicular bone. Clinical orthopaedics and related research. 1984 Sep 1(188):230-3.
197. Weidmann CE, Ganz PA. Multiple synchronous lesions of acral metastasis. Western Journal of Medicine. 1984 Mar;140(3):451.
198. Kosuda SH, Gokan TA, Tamura KO, Dokiya TA, Kubo AT, Hashimoto SH. Radionuclide imaging of two patients with metastasis to a distal phalanx of the hand. Clinical nuclear medicine. 1986 Sep 1;11(9):659-60.
199. Henderson JJ. Metastatic carcinoma in the hand presenting as an acute paronychia. The British journal of clinical practice. 1987 Jun;41(6):805-6.
200. Sim E. Misinterpretation of a metastatic process within the scope of Sudeck's dystrophy. A case report. Aktuelle Traumatologie. 1989 Apr;19(2):85-9.
201. Farouk AR, McBride DJ, Bingham J. A case of tumour simulating pulp space infection. The British journal of clinical practice. 1990 Dec 1;44(12):737-8.
202. Stone RL, Davies JP. Isolated digital metastasis in bronchogenic carcinoma. Palliative medicine. 1990 Apr;4(2):137-8.
203. Desmanet E, Amrani M, Fievez R, Six C. Acrometastases. Apropos of 2 cases. Review of the literature. Annales de chirurgie de la main et du membre superieur: organe officiel des societes de chirurgie de la main= Annals of hand and upper limb surgery. 1991 Jan 1;10(2):154-7.
204. Moens P, De Smet L, Fabry G, Van Damme B. Acrometastasis of the hand presenting as a whitlow. General review apropos of a case. Revue de chirurgie orthopedique et reparatrice de l'appareil moteur. 1993 Jan 1;79(1):66-9.
205. De Maeseneer M, Machiels F, Naegels S, Verhaeghe W. Hand and foot acrometastases in a patient with bronchial carcinoma. Journal belge de radiologie. 1995 Oct 1;78(5):274-5.
206. Saglike Y, Demirtas M, Demirors H. Metastatic tumours of the hand. International orthopaedics. 1996 Apr;20(2):123-4.
207. Hatakeyama S, Tachibana A, Suzuki K, Okano H. A case of metastasis to the metacarpal bone of the right thumb from squamous cell carcinoma of the lung [Japanese, abstract]. Jpn J Lung Cancer. 1997;37:531-535.
208. Baran R, Guillot P, Tosti A. Metastasis from carcinoma of the bronchus to the distal aspect of two digits. The British journal of dermatology. 1998 Apr 1;138(4):708-.
209. Chang SE, Choi JH, Sung KJ, Moon KC, Koh JK. Metastatic squamous cell carcinoma of the nail bed: a presenting sign of lung cancer. The British journal of dermatology. 1999 Nov 1;141(5):939-40.
210. Vanhooteghem O, Dumont M, Andre J, Leempoel M, De La Brassinne M. Bilateral subungual metastasis from squamous cell carcinoma of the lung: a diagnostic trap!. Revue medicale de Liege. 1999 Aug 1;54(8):653-4.
211. Ryu JS, Cho JW, Moon TH, Lee HL, Han HS, Choi GS. Squamous cell lung cancer with solitary subungual metastasis. Yonsei medical journal. 2000 Oct 1;41(5):666-8.
212. Caglar M, Ceylan E. Isolated carpal bone metastases from bronchogenic cancer evident on bone scintigraphy. Clinical nuclear medicine. 2001 Apr 1;26(4):352-3.
213. Theunissen CC, Engelkens HJ, Mulder LJ, Dees A. Diagnostic image (107). A man with weight loss and skin nodes. Nederlands tijdschrift voor geneeskunde. 2002 Sep 28;146(39):1832.
214. Sahbaz S, Kilinç O, Vayvada H, Topçu A, Yörükoğlu K, Uçan ES. Distal phalanx metastasis in operated bronchial carcinoma. Tuberkuloz ve toraks. 2004 Jan 1;52(4):378-81.
215. Nakamura H, Shimizu T, Kodama K, Shimizu H. Metastasis of lung cancer to the finger: a report of two cases. International journal of dermatology. 2005 Jan;44(1):47-9.
216. Gawley B, Motykie G, Piazza RC, Holding J, Gould LJ. Rapid progression of metastatic bronchogenic carcinoma after felon drainage. Orthopedics. 2006 Nov 1;29(11):1035-36
217. Elhassan B, Fakhouri A. Metastasis of squamous-cell carcinoma of the lung to the first web space of the hand. The Journal of bone and joint surgery. British volume. 2007 Sep;89(9):1243-6.
218. Bahar T, Borman H, Ertas NM, Seyhan T. Three years' survival after diagnosis of finger metastasis from end-stage lung cancer. Dermatologic surgery. 2008 Aug 1;34(8):1128-30.
219. Madjidi A, Cole P, Laucirica R. Digital acrometastasis: a rare initial sign of occult pulmonary squamous cell carcinoma. Journal of Plastic, Reconstructive & Aesthetic Surgery. 2009 Oct 1;62(10):e365-7.
220. Lozić AA, Besser Silconi Ž, Mišljenović N. Metastases to rare locations as the initial manifestation of non-small cell lung cancer: two case reports. Collegium antropologicum. 2010 Jun 30;34(2):609-12.
221. Lucilli N, Mattacheo A, Palacios A. Acrometastasis due to lung cancer. A case presentation. Archivos de Bronconeumologia (English Edition). 2010;5(46):279-80.
222. Chao D, Harinarayanan S, Reynolds D. The acromet. J Thorac Dis. 2011;3(3):209-210.
223. Ingerslev K. Digital metastasis as an unusual primary symptom of cancer. Ugeskrift for laeger. 2014 Dec 1;176(25A).
224. Lambe G, Le P, Clay TD. A finding with a diagnosis: I just can't put my finger on it. Case Reports. 2014 Dec 22;2014:bcr2014208665.
225. Walton ZJ, Holmes RE, Chapin RW, Lindsey KG, Leddy LR. Bronchogenic squamous cell carcinoma with soft-tissue metastasis to the hand: an unusual case presentation and review of the literature. Am J Orthop. 2014 Dec 1;43(12):E324-7.
226. Babacan NA, Kiliçkap S, Sene S, Kacan T, Yucel B, Eren MF, Cihan S. A case of multifocal skin metastases from lung cancer presenting with vasculitic-type cutaneous nodule. Indian journal of dermatology. 2015 Mar;60(2):213.
227. Baltazard T, Arnault JP, Dillied AS, Joly JP, Lok C. What is the prognostic significance of acrometastases?. Dermatology online journal. 2015;21(9).
228. Soylemez S, Demiroglu M, Yayla MA, Ozkan K, Alpan B, Ozger H. Lung metastasis mimicking fingertip infection. Case reports in oncological medicine. 2015 Jul 7;2015.
229. Sahoo TK, Das SK, Majumdar SK, Senapati SN, Parida DK. Digital acrometastasis as initial presentation in carcinoma of lung a case report and review of literature. Journal of clinical and diagnostic research: JCDR. 2016 Jun;10(6):XD01.
230. Muñoz-Mahamud E, Combalia A, Carreño A, Arandes JM. Five cases of acrometastasis to the hand from a carcinoma and review of the literature. Hand Surgery and Rehabilitation. 2017 Feb 1;36(1):12-6.
231. Ross RJ, Mann NC. Synchronous distal phalangeal metastases from primary non-small-cell lung cancer. The Lancet. 2017 Jul 22;390(10092):399.
232. Espinosa JA, Hollings D, Nagpal S, Pratt JW. A rare case of hand pain and metacarpal metastasis as the presenting symptom of lung cancer. Innovations. 2018;13(1S):S33-34.
233. Castillo R, Albayda J. Distal Hand Metastases: Clinical and Ultrasonographic Appearance. JCR: Journal of Clinical Rheumatology. 2019 Aug 1;25(5):e69-70.
234. Khaja M, Mundt D, Dudekula RA, Ashraf U, Mehershahi S, Niazi M, Lvovsky D, Malik S, Diaz-Fuentes G. Lung cancer presenting as skin metastasis of the back and hand: a case series and literature review. Case reports in oncology. 2019;12(2):480-7.
235. Peeters CM, Gosens T. Metastasis from lung carcinoma to the finger: A case report. Acta Orthopædica Belgica. 2019 Mar 1;85:1-2019.
236. Garabet Diramerian L, Griffin E, Pendergrast K, Arsura E, Roberts M. Metastatic Lung Cancer to the Distal Finger Presenting as Osteomyelitis. Cureus. 2020 Nov;12(11).
237. Hirano Y, Sato R, Ohshima N, Matsui H. Acrometastasis of Lung Cancer. Internal Medicine. 2020 Aug 1;59(15):1919-20.
238. Cottignoli C, Romagnolo C, Fringuelli FM, Burroni L. Lung cancer metastasis mimicking fingertip osteomyelitis: An 18F-fluorodeoxyglucose positron emission tomography-computed tomography study. World Journal of Nuclear Medicine. 2021 Apr 1;20(2):185.
239. Nivar I, Panico LC, Yoo C, Parrish W. Trapezial Acrometastasis as the First Presentation of Occult Lung Cancer: A Case Report. JBJS Case Connector. 2021 Apr 1;11(2):e20.
240. Mangini UM. Tumors of the skeleton of the hand. Bulletin of the Hospital for Joint Diseases. 1967;28(2):61.
241. Michel PJ, Colson P, Pipard C, Grimand P. Epithélioma ulcéré du dos de la main, métastase exceptionnelle par sa localisation et restée unique pendant plusieurs années d'un cancer du sein opéré 30 ans auparavant. Bull Soc Fr Dermatol Syphiligr. 1967;74:780-3.
242. Panebianco AC, Kaupp HA. Bilateral thumb metastasis from breast carcinoma. Archives of Surgery. 1968 Feb 1;96(2):216-8.
243. Nadzhmitdinov NN, Zadarski LT. Rare site of metastasis of a breast cancer. Klin Khir. 1970;5:50-1.
244. Kumar PP, Kovi J. Metastases to bones of the hands and feet. Journal of the National Medical Association. 1978 Nov;70(11):837.
245. Liszka G, Péter Z, Hérics I. " Rare localisation" of bone metastases by patients with primary mamma carcinoma (author's transl). Magyar traumatologia, orthopaedia es helyreallito sebeszet. 1980 Jan 1;23(1):64-9.
246. Vijayakumar S, Creditor M. Metastasis to the Hand. J Natl Med Assoc. 1986;78(5):441-442.
247. Vadivelu R, Drew S. Phalangeal metastasis secondary to an occult breast carcinoma. Archives of orthopaedic and trauma surgery. 2002 Jan 1;122(9-10):530-1.
248. French FP, Murray PM, Perdikis G. Extensive cutaneous metastatic breast carcinoma of the hand and upper extremity: a case report. The Journal of hand surgery. 2007 Feb 1;32(2):252-5.
249. Wavreille G, Baroncini M, Rtaimate M. A rare cause of pseudoclubbing (pseudohippocratic finger): the acrometastasis. A case report. Chirurgie de la Main. 2009 Sep 20;28(6):381-3.
250. Biyi A, Oufroukhi Y, Doudouh A. Hand and foot acrometastasis secondary to breast carcinoma. Chirurgie de la main. 2009 Sep 17;29(1):40-3.
251. Brygger L, Cold S. Acrometastasis from breast cancer after the use of frozen gloves by adjuvant chemotherapy. Ugeskrift for laeger. 2015 Jan 1;177(2A):26-7.
252. Basora J, Fery A. Metastatic malignancy of the hand. Clinical orthopaedics and related research. 1975;108:182-6.
253. Patel MR, Anand VS, Desai SS. Metastatic Tumor of the Hand from Malignant Cystosarcoma Phylloides of the Breast: A Case Report. Orthopedics. 1985 Mar 1;8(3):373-5.
254. Looi CS, Arumugam M. Acrometastases: The nasty alter ego of fingertip infections. Malaysian family physician: the official journal of the Academy of Family Physicians of Malaysia. 2021 Jul 22;16(2):86.
255. Umebayashi Y. Distal phalangeal metastasis of extramammary Paget's disease. The Journal of dermatology. 2004 Jan;31(1):63-5.
256. Wu CY, Gao HW, Huang WH, Chao CM. Infection-like acral cutaneous metastasis as the presenting sign of an occult breast cancer. Clinical and experimental dermatology. 2009 Oct;34(7):e409-10.
257. Ravind R, Prameela CG, Gurram BC, Dinesh M. Synchronous phalangeal metastases in upper and lower limbs from primary breast malignancy: a rare case scenario with a review of the literature. Case Reports. 2015 Dec 23;2015:bcr2015213246.
258. Toth JG. Metastasis to the fingers from breast cancer. Canadian Medical Association Journal. 1983 Sep 15;129(6):534.
259. Ammons A, Garcia A, Cable M. Acral Metastasis: An Uncommon Site of Cancer Spread. In journal of investigative medicine. 2021;69(2):548.
260. Carty HM, Simons AW, Isgar B. Breast carcinoma bone metastasis first presenting to single middle phalanx. The Breast. 2006 Feb 1;15(1):127-9.
261. Vijaya B, Veeranna S, Manjunath G. Erythematous nodules of the hand: A rare site of metastatic breast carcinoma. Indian journal of dermatology, venereology and leprology. 2011 Nov 1;77(6):695.
262. De Smet L. Late appearance and slow progression of a breast carcinoma metastasis of the index metacarpal. Acta Chirurgica Belgica. 2004 Jan 1;104(3):345-6.
263. Handley WS. Cancer of the breast and its operative treatment. Murray.1906.
264. Bendick AJ, Jacobs AW. Report of a case of extensive generalized skeletal metastases following primary carcinoma of the breast. AJR Am J Roentgenol. 1925;14:35-8.
265. Pack GT. Symposium: Tumors of the Hands and Feet: Introduction. Surgery. 1939 Jan 1;5(1):1-26.
266. Grilli FP. Su due casi di metastasi carcinomatosa delle ossa della mano. Tedazione. 1958;30:386-93.
267. Toubiana CG, Proux C. Les metastases osseuses distales. Annales de radologie-radiologie Clinique radiobiologie. 1965 Jan 1;8(3-4):R217.
268. Carroll RE. Tumors of the hand skeleton. Hand Surgery. 2nd ed. Baltimore: Williams and Wilkins. 1975;678.
269. Bloom RA, Sulkes A, Freilick G, Libson E. Breast metastases to bones of the extremities: simultaneous involvement of all four limbs. Clinical oncology. 1992 Jan 1;4(1):58-9.
270. Witthaut, J. and Steffens, K., 1996. Misinterpretation of a solitary breast carcinoma metastasis of the hand as an enchondroma. Case report and literature review. Handchirurgie, Mikrochirurgie, Plastische Chirurgie: Organ der Deutschsprachigen Arbeitsgemeinschaft fur Handchirurgie: Organ der Deutschsprachigen Arbeitsgemeinschaft fur Mikrochirurgie der Peripheren Nerven und Gefasse: Organ der V.1996;28(5):271-277.
271. Kaplan MJ, Propeck T, Fessell DP. Diffuse swelling of one finger in a patient with metastatic breast cancer. Journal of clinical rheumatology: practical reports on rheumatic & musculoskeletal diseases. 2000 Aug 1;6(4):228-30.
272. Asthana S, Deo SV, Shukla NK, Raina V. Carcinoma breast metastatic to the hand and the foot. Australasian radiology. 2001 Aug 15;45(3):380-2.
273. Cattelan M, Dumontier C. Metastatic tumor of the hand-Three new cases and a literature review. Journal of Plastic, Reconstructive & Aesthetic Surgery. 2021 Apr 19.
274. Reichbach EJ, Levinson JD, Fagin RR. Unusual osseous metastases of hepatoma. JAMA. 1970 Sep 21;213(12):2078-9.
275. Delsmann BM, Lienemann A, Nerlich A, Hoffmann E, Caselmann WH, Refior HJ. Primary manifestation of hepatocellular carcinoma as osteolytic hand metastasis--a case report. Zeitschrift fur Orthopadie und ihre Grenzgebiete. 1998 Nov 1;136(6):571-3.
276. Lee KS, Lee SH, Kang KH, Oh KJ. Metastatic hepatocellular carcinoma of the distal phalanx of the thumb. Hand Surg. 1999;4(1): 95-100.
277. Fang YR, Huang YS, Wu JC, Chao Y, Tsay SH, Chan CY, Chang FY, Lee SD. An Un usual Cu ta ne ous Me tas ta sis from Hepatocellular Car ci noma. Chin Med J (Tai pei). 2001;64:253-7.
278. Fontana T, Siciliano M, Franceschelli A, Annicchiarico BE, Rossi P, Bigotti G, Bombardieri G. An atypical bone metastasis of hepatocellular carcinoma: case report and review of the literature. La Clinica Terapeutica. 2004 Oct 1;155(10):447-51.
279. Corrales Pinzón R, Sánchez JA, de la Mano González S, Tarazona KE. Metástasis única en los huesos carpianos como primera manifestación clínica de un hepatocarcinoma. Radiología. 2014 Nov 1;56(6):e42-5.
280. Rauer T, Gorzelany K, Rindlisbacher A, Zünd, M, Acrometastasis - A rare initial sign of a hepatocellular carcinoma (HCC). Zuger Kantonsspital. 2016.
281. Otsuji M, Matsunaga S, Koga H, Kawabata N, Imakiire T, Hiwaki T, Tashiro Y, Shirahama H, Komiya S. An atypical extrahepatic metastasis of the distal phalanx from hepatocellular carcinoma. International journal of clinical oncology. 2009 Apr;14(2):159-62.
282. Kim JI, Song CH, Gong HS. Finger skin metastasis from hepatocellular carcinoma: a case report. Hand Surg. 2012;17(1):131-134.
283. Rauf MS, Motta L, Connolly C. Digital acrometastases as first sign of hepatocellular carcinoma. Scottish medical journal. 2012 Nov;57(4):1-3.
284. Rommer E, Leilabadi SN, Lam G, Soltani A, Ellis CV, Rizvi M, Wong AK. Metastasis of hepatocellular and renal cell carcinoma to the hand. Plast Reconstr Surg Glob Open. 2013;1(9):1-4. doi:10.1097/GOX.0000000000000020
285. Boldo E, Santafe A, Mayol A, Lozoya R, Coret A, Escribano D, Fortea-Sanchis C, Muñoz A, Pastor JC, de Lucia GP, Bosch N. Rare Site Hepatocellular Carcinoma Metastasis. Journal of hepatocellular carcinoma. 2020;7:39.
286. Mousavi SR, Ghasemi A, Tajodini A. Metastasis of esophageal cancer to finger. Arch Iranian Med. 2005;8(3):319-320.
287. Wurapa RK, Bickel BA, Mayerson J, Mowbray JG. Metastatic esophageal adenocarcinoma of the carpus. Am J Orthop (Belle Mead NJ). 2010;39(6):283-285.
288. Jenzer A, Badur N, Vögelin E. Composite bone cement arthrodesis in acrometastasis of the proximal phalanx of the hand—a case report. Internet J Orthop Surg. 2011;19(1).
289. Kamolz LP, Stiglbauer W, Längle F. Palmar metastasis of an adenocarcinoma of the esophago-gastric-junction: First case report. International journal of surgery case reports. 2012 Jan 1;3(9):412-4.
290. Zhang YJ, Wang YY, Yang Q, Li JB. Scaphoid metastasis as the first sign of occult gastroesophageal junction cancer: A case report. World journal of clinical cases. 2020 Apr 6;8(7):1287.
291. Katsumata F, Kamiya K, Okada H, Maekawa T, Komine M, Ohtsuki M. A case of finger metastasis from esophageal basaloid squamous cell carcinoma. International journal of dermatology. 2021 Nov 23.
292. Kanatani T, Fujioka H, Yamasaki K. Re: Metastatic oesophageal carcinoma presenting in a finger. Journal of Hand Surgery (European Volume). 2008 Aug;33(4):537-8.
293. Zakharov BI. A case of metastasization of oesophageal cancer into the ungual phalanx. Ventnik Roentgenol. Radiol. 1970;45:102-3.
294. Kumar PP. Metastases to the bones of the hand. Journal of the National Medical Association. 1975 Jul;67(4):275.
295. Levack B, Scott G, Flanagan JP. Metastatic carcinoma presenting as a pulp space infection. The Hand. 1983 Oct 1;15(3):341-2.
296. Haas O, Bernard A, Cougard P, Viard H. Phalangeal metastasis of cancer of the esophagus. Apropos of a case [in French]. Ann Chir. 1988;42(1):37-38.
297. Tenenbaum F, Reverberi J, Vacher B, Pulik M. Metastasis in the carpal bones and cancer of the esophagus. Journal de chirurgie. 1988;125(6-7):443-4.
298. Moutet F, Bellon-Champel P, Lebrun C, Sarrazin R. Isolated metastasis to the capitate bone. Annales de chirurgie de la main et du membre superieur: organe officiel des societes de chirurgie de la main= Annals of hand and upper limb surgery. 1991;10(2):148-50.
299. Umebayashi Y. Metastasis of esophageal carcinoma manifesting as whitlow‐like lesions. The Journal of dermatology. 1998 Apr;25(4):256-9.
300. Yasaka N, Ando I, Kukita A. An acral ‘inflammatory’cutaneous metastasis of oesophageal carcinoma. British Journal of Dermatology. 1999 Nov;141(5):938-9.
301. Houston JD, Telepak RJ. An isolated digital metastasis of esophageal basaloid squamous cell carcinoma. Clinical nuclear medicine. 2000 Jul 1;25(7):557-8.
302. Silfen R, Amir A, Tobar A, Hauben DJ. The digital pulp as a presenting site of metastatic esophageal carcinoma. Annals of plastic surgery. 2001 Feb 1;46(2):183-4.
303. Bujanda AD, Bohn Sarmiento U, Aguiar Morales J. Some Unusual Paraneoplastic Syndromes: Case 1. Metastatic Squamous Cell Esophageal Cancer to the Thumb. Journal of clinical oncology. 2003 Jul 1;21(13):2620-.
304. Dimri K, Neeraj R, Punita L. Carcinoma of esophagus with unusual metastasis to gingiva and phalanx. Indian journal of cancer. 2003 Jan 1;40(1):37-9.
305. Chou YC, Lin JY, Chen HH, HO HC. A patient of esophageal carcinoma presenting with fever and multiple nodules on fingers. Dermatologica Sinica. 2004;22(2):199-200.
306. Hsieh CY, Bai LY, Lo WC, Huang HH, Chiu CF. Esophageal squamous cell carcinoma with a solitary phalangeal metastasis. Southern medical journal. 2008 Nov 1;101(11):1159-60.
307. Dar AM, Sharma ML, Bhat MA. Unusual metastasis to all the digits of both hands in a patient previously operated on for esophageal carcinoma. General thoracic and cardiovascular surgery. 2011 Mar;59(3):225-7.
308. Purkayastha J. Isolated bony metastasis to upper limb from carcinoma of the oesophagus: report of three cases. HAND. 2015 Mar;10(1):137-9.
309. Arbeláez Echeverri P, García MF, Garzón J, Morales LC, Messa Botero O, Zúñiga MI, Lozada Mujica CJ. Acrometástasis óseas, serie de casos y revisión de la literatura. Revista chilena de radiología. 2019 Oct;25(3):87-93.
310. Chaudhary S, Caplash Y, Foster-Smith E. Metastatic squamous cell carcinoma of distal phalanx presenting incidentally from a presumed subungal haematoma: a unique case. ANZ journal of surgery. 2021 May;91(5):1028-30.
311. Craigen MA, Chesney RB. Metastatic adenocarcinoma of the carpus: a case report. The Journal of Hand Surgery: British & European Volume. 1988 Aug 1;13(3):306-7.
312. DiSpaltro FX, Bickley LK, Nissenblatt N, Devereux D. Cutaneous acral metastasis in a patient with primary gastric adenocarcinoma. Journal of the American Academy of Dermatology. 1992;27(1):117-8.
313. Okada H, Qing J, Ohnishi T, Watanabe S. Metastasis of gastric carcinoma to a finger. The British journal of dermatology. 1999 Apr;140(4):776-7.
314. Chang HC, Lew KH, Low CO. Metastasis of an adenocarcinoma of the stomach to the 4th metacarpal bone. Hand Surgery. 2001 Dec;6(02):239-42.
315. Bahk WJ, Rhee SK, Kang YK, Lee AH, Park JM, Chung YG. Gastric cancer acrometastases to all digits of one hand following closed intramedullary nailing. Skeletal radiology. 2006 Jul;35(7):529-32.
316. Park KH, Rho YH, Choi SJ, Hong SJ, Lee JH, Choi IK, Kim SJ, Seo JH, Choi CW, Kim BS, Shin SW. Acute arthritis of carpal bones secondary to metastatic gastric cancer. Clinical rheumatology. 2006 Mar;25(2):258-61.
317. Bigot P, Desbois E, Benoist N, Besnier L, Moui Y. Isolated pain of the hand revealing a metastatic tumor of the hand. Report of a case. Chirurgie de la Main. 2007 Oct 22;26(6):300-2.
318. Miyamoto W, Yamamoto S, Uchio Y. Metastasis of gastric cancer to the fifth metacarpal bone. Hand Surgery. 2008;13(03):193-5.
319. Spiteri V, Bibra A, Ashwood N, Cobb J. Managing acrometastases treatment strategy with a case illustration. Annals of The Royal College of Surgeons of England. 2008 Oct;90(7):W8.
320. Naito K, Furuya H, Han C, Orita H, Sato K, Kaneko K, Obayashi O. Gastric cancer with metastases to the scaphoid: a case report. Journal of Orthopaedic Science. 2015 Jan;20(1):213-6.
321. Okamoto M, Yamazaki H, Yoshimura Y, Aoki K, Tanaka A, Kato H. Massive trapezial metastasis from gastric adenocarcinoma resected and reconstructed with a vascularized scapular bone graft: a case report. Medicine. 2017 Dec;96(50).
322. Gomi D, Fukushima T, Kobayashi T, Sekiguchi N, Sakamoto A, Mamiya K, Koizumi T. Gastric cancer initially presenting as bone metastasis: Two case reports and a literature review. Oncology letters. 2018 Nov 1;16(5):5863-7.
323. Kumar A. Acrometastases to the hand in stomach carcinoma: a rare entity. BMJ Case Reports CP. 2019 Aug 1;12(8):e229390.
324. Harris H, Khan M, Jaunoo S. Distal phalanx: an unusual site for a gastric adenocarcinoma metastasis. BMJ Case Reports CP. 2020 Sep 1;13(9):e236259.
325. Drury BJ. Adenocarcinoma of the rectum with metastasis to the nail-bed of the finger. California medicine. 1959 Jul;91(1):35.
326. Hummel J, Scott RM. Adenocarcinoma of the rectum with metastases to a phalanx; A Case Report. The Journal of the Kentucky Medical Association. 1962 Feb;60:158-9.
327. Guttmann G, Stein I. Metastatic tumor of the thumb from adenocarcinoma of the colon. International surgery. 1968 Mar;49(3):217-21.
328. Gottlieb JA, Schermer DR. Cutaneous metastases from carcinoma of the colon. JAMA. 1970;213(12):2083.
329. Bryan RS, Soule EH, Dobyns JH, Pritchard DJ, Linscheid RL. Metastatic lesions of the hand and forearm. Clinical Orthopaedics and Related Research (1976-2007). 1974 Jun 1;101:167-70.
330. Buckley N, Brown P. Metastatic tumors in the hand from adenocarcinoma of the colon. Diseases of the colon & rectum. 1987 Feb 1;30(2):141-3.
331. Hindley CJ, Metcalfe JW. A colonic metastatic tumor in the hand. The Journal of hand surgery. 1987 Sep 1;12(5 Pt 1):803-5.
332. Bourne MH, Amadio PC, Wold LE, Sim FH. Metastatic lesions of the hand: report of a case. Orthopedics. 1988 Jan 1;11(1):219-21.
333. Müller S, Dörner A, Dallek M, Supra T. Hand metastases--a rare metastatic form of colorectal carcinoma. Deutsche medizinische Wochenschrift (1946). 1988 May 1;113(18):728-30.
334. Mendez Lopez JM, Garcia Mas R, Salva Coll G. Metastasis of an adenocarcinoma of the colon to the 1st metacarpal bone. Annales de chirurgie de la main et du membre superieur: organe officiel des societes de chirurgie de la main= Annals of hand and upper limb surgery. 1997 Jan 1;16(2):134-7.
335. Rümenapf G, Rupprecht H, Groitl H, Grünert J, Hohenberger W. Fingermetastase eines Kolonkarzinoms. coloproctology. 1997 Jul;19(4):169-72.
336. Baron JM, Borrego P, Solis JA. Metastasis en la mano, a proposito de un caso. Oncologia. 1998;21(6):184-6.
337. Augustine AJ, Pai KR, Nagendhar MY. Colonic carcinoma presenting with metastasis to finger. Indian Journal of Gastroenterology. 2000 Jul 1;19(3).
338. Henderson MC, Jehangir S. A swollen finger and abdominal pain. The Lancet. 2001 Aug 25;358(9282):636.
339. Oron A, Reshef N, Siegelmann-Danieli N, Lin E, Aghasi M. Colon cancer metastazing to the proximal phalanx of an index finger—A case report. The Journal of Hand Surgery. 2003 Jan 1;28:26-7.
340. Mátrai Z, Péley G, Farkas E, Kovács T, Köves I. The similarities between the mechanism of wound healing and tumor development--literature review on the occasion of a patient with colonic adenocarcinoma metastasis in a dog-bite wound. Orvosi hetilap. 2005 Jan 1;146(3):99-109.
341. Ozcanli H, Ozdemir H, Ozenci AM, Söyüncü Y, Aydin AT. Metastatic tumors of the hand in three cases. Acta Orthop Traumatol Turc. 2005 Jan 1;39(5):445-8.
342. Gallagher B, Yousef G, Bishop L. Subungual metastasis from a rectal primary: case report and review of the literature. Dermatologic surgery. 2006 Apr;32(4):592-5.
343. Gamblin TC, Santos RS, Baratz M, Landreneau RJ. Metastatic colon cancer to the hand. The American Surgeon. 2006 Jan;72(1):98-100.
344. Ishikawa N, Tanaka N, Yokoi K, Seya T, Horiba K, Oaki Y, Tajiri T. A case of rectal metastatic tumor in the soft tissue of the hand. Journal of Nippon Medical School. 2007;74(4):309-13.
345. Nikolić I, Patrnogić A, Stojiljković B, Bogdanović B, Kukić B. Thumb metastases in a patient with colorectal cancer. Arch Oncol. 2007;15(3e4):97e98.
346. Anoop TM, George S, Divya KP, Jabbar PK. Metastatic phalangeal osteolysis as an initial presentation of carcinoma colon. The American Journal of Surgery. 2010 Nov 1;200(5):e61-3.
347. Borobio León G, García Plaza A, García Cepeda I, González Alconada R, Hernández Cosido L. Hand metastasis of rectal cancer. An unusual case [in Spanish]. Cir Esp. 2010;88(3):195-197.
348. Vasić L. Osteolysis of hand bones due to metastatic deposits from colon cancer: A case report. Medicinski pregled. 2010;63(9-10):719-22.
349. Çetın B, Büyükberber S, Yüksel M, Coşkun U, Yildiz R, Beneklı M. Metastasis of rectal cancer to soft tissue of the hand: an unusual case. The Turkish journal of gastroenterology: the official journal of Turkish Society of Gastroenterology. 2011;22(2):229-30.
350. Roohi SA, Samsudin OC, Shukur MH, Ibrahim S. Metastatic Adenocarcinoma to the Wrist Presenting as Carpal Tunnel Syndrome: A Report of an Unusual Cause. Malaysian Orthopaedic Journal. 2011;5(1):75-7.
351. Fadli AR, Azmi MN, David O, Zailani MH. Isolated metacarpal bone metastasis from advanced rectosigmoid carcinoma. IIUM Medical Journal Malaysia. 2012 Jun 1;11(1).59-61
352. Gharwan H, Yarlagadda L, Duffy A. Acrometastasis as the Initial Presentation of aKRAS-Positive Colon Cancer. Case reports in oncology. 2012;5(2):404-8.
353. Ko JH, Young A, Wang KH. Paronychia-like digital cutaneous metastasis. British Journal of Dermatology. 2014 Sep 1;171(3):663-5.
354. Wang XX, Liu HQ, Sui JC. Distal Bone Metastasis From Primary Rectal Cancer: A Case Report. American journal of therapeutics. 2016 May 1;23(3):e926-9.
355. Ito H, Horie H, Sadatomo A, Naoi D, Tahara M, Kono Y, Inoue Y, Koinuma K, Lefor AK, Sata N. Metachronous solitary metacarpal bone metastasis from rectal cancer. Journal of surgical case reports. 2017 Dec;2017(12):rjx247.
356. Rau ME, Rau ME. All Hands on Deck: A Rare Case of Metastatic Colorectal Cancer. In journal of the American geriatrics society 2019. 2019 Apr 1;66:S183-183.
357. Voskuil RT, Smith JR, Swafford RE, Jemison DM. Colon adenocarcinoma with metastases to the scaphoid: a case report and review of the literature. Journal of surgical case reports. 2019 Feb;2019(2):rjz011.
358. Gordinou de Gouberville MC, Hoeijmakers F, de Waard JD. A rare cause of swelling of the little finger. Nederlands Tijdschrift Voor Geneeskunde. 2021 Feb 4;165.
359. Verardino GC, Silva RS, Obadia DL, Gripp AC, Alves MD. Rare cutaneous metastasis from a probable basaloid carcinoma of the colon mimicking pyogenic granuloma. Anais brasileiros de dermatologia. 2011;86:537-40.
360. Brette M, Grandmottet P, Tissot A. Métastase métacarpienne isolée d'un épithélioma colique. Lyonmed. 1961;205:733-9.
361. Lichtenstein L. Bone Tumors. St. Louis: CV Mosby Co. 1952;283
362. Molina-Cerrillo J, Barquin-García A, Alonso-Gordoa T. Phalangeal metastasis in colon adenocarcinoma. Med. clín (Ed. impr.). 2019:e23-.
363. Henkert K, Berge G. Malignant metastases on the hand. Zentralblatt fur Chirurgie. 1991 Jan 1;116(5):337-41.
364. Balta AZ, Sinan H, Ozdemir Y, Yucel E, Ince M. Gastrointestinal: Palmar metastasis in undifferentiated colonic carcinoma. Journal of gastroenterology and hepatology. 2011 Feb;26(2):412-.
365. Fabre P, Dambrin P. Tumeur pseudo-aneurysmale de la main. Ann. d'anat. path. 1935;12:380-383
366. Kinsella JD. Unusual metastasis of a renal cell carcinoma. Southern Medical Journal. 1957;50(6):803-5.
367. Schmitt-Köppler A, Richter G. Repeated surgical therapy of metastases in a patient with hypernephroma. Zentralblatt fur Chirurgie. 1969 Feb 1;94(6):193-8.
368. De Oliveira JA, Silveira E, Marinst JL. Metastasis from renal carcinoma to a phalanx of the hand. British journal of urology. 1978 Aug;50(4):280-.
369. Kobus RJ, Leinberry C, Kirkpatrick WH. Metastatic renal carcinoma in the hand: treatment with preoperative irradiation and ray resection. Orthopaedic review. 1992 Aug 1;21(8):983-4.
370. Vine JE, Cohen PR. Renal Cell Carcinoma Metastatic to the Thumb: A Case Report and Review of Subungual Metastases From All Primary Sites. The Journal of Urology. 1998 Feb;159(2S):618-9.
371. Adegboyega PA, Adesokan A, Viegas SF. Acrometastasis in renal cell carcinoma. South Med J. 1999;92(10):1009-1012.
372. Ghert MA, Harrelson JM, Scully SP. Solitary renal cell carcinoma metastasis to the hand: the need for wide excision or amputation. The Journal of hand surgery. 2001 Jan 1;26(1):156-60.
373. Tolo ET, Cooney WP, Wenger DE. Renal cell carcinoma with metastases to the triquetrum: case report. The Journal of hand surgery. 2002 Sep 1;27(5):876-81.
374. Mitrovic R, Pakevic N, Jeremic B, Cesarevic D, Zivanovic P. S74 Renal cell carcinoma metastasis to hand: rare localisations of solitary metastases. European Urology Supplements. 2009 Sep 1;8(8):632.
375. Anglada-Curado FJ, De Haro-Padilla J, Carrasco-Valiente J, Alvarez-Kindelan J, Ruiz-Garcia J, Requena-Tapia MJ. Hand metastasis from renal carcinoma. Urology. 2010 Oct 1;76(4):846.
376. Beach DF, Somer R. Metastatic Renal Cell Carcinoma to the Phalanx. Urology. 2012 May 1;79(5):e77.
377. Borgohain B, Borgohain N, Khonglah T, Bareh J. Occult renal cell carcinoma with acrometastasis and ipsilateral juxta-articular knee lesions mimicking acute inflammation. Advanced biomedical research. 2012;1.48.
378. Hernández-Cortés P, Caba-Molina M, Gómez-Sánchez R, Ríos-Peregrina R. Renal clear cell carcinoma acrometastasis. an unusual terminal condition. Journal of hand and microsurgery. 2015 Jun;7(01):149-51.
379. Humphries LS, Baluch DA, Nystrom LM, Borys D, Bednar MS. Interfascicular renal cell carcinoma metastasis to the ulnar nerve: A case report. Hand. 2016 Jun;11(2):NP1-4.
380. Kumar AM. Finger metastases in renal cell carcinoma: Acase report. Asian J Pharm Clin Res. 2016;9(1):4-6.
381. Selvi F, Faquin WC, Michaelson MD, August M. Three synchronous atypical metastases of clear cell renal carcinoma to the maxillary gingiva, scalp and the distal phalanx of the fifth digit: a case report. Journal of Oral and Maxillofacial Surgery. 2016 Jun 1;74(6):1286-e1.
382. Żyluk A, Janowski P. Metastasis of renal cancer to the wrist and hand: a case report. Pomeranian journal of life sciences. 2016 Dec 8;62(2).
383. Lechmiannandan NA, Paul AG, Thevarajah S. “Thumb's off” for acrometastasis of renal cell carcinoma: Is there a role for acrometastasectomy in the era of targeted therapy?. Asian journal of urology. 2018 Jul;5(3):199.
384. Oshina T, Azuma T. Finger metastasis from renal cell carcinoma. BMJ. 2018 Apr 26;361.
385. Ho PS, Yip LY, Nguyen M, Wijesinghe W, Sahu A. A Painful Finger: An Unusual Presentation of Von Hippel-Lindau-Associated Advanced Renal Cell Carcinoma. Case reports in oncology. 2020;13(1):245-8.
386. Milionis V, Vlachodimitropoulos D, Goutas D, Goutas N (2020) Acrometastases in Renal Cell Carcinoma: A Case Report and Review of Literature. Clin Oncol Case Rep. 2020;2:2.
387. Rao SB, Suresh PK, Fernandes M, Prabhu LG, Sreeram S, Annappa R. Diagnostic dilemma of a thumb swelling: An unpropitious turn of events. Journal of Cancer Research and Therapeutics. 2020 Oct 1;16(4):926.
388. Greither A, Tritsch H. Die Geschwülste der Haut: ihr klinisches und feingewebliches Bild; ihre Erkennung und Behandlung. Thieme; 1957.
389. Barnett LS, Morris JM. Metastases of renal-cell carcinoma simultaneously to a finger and a toe: a case report. JBJS. 1969 Jun 1;51(4):773-4.
390. Bunnell S. Surgery of the Hand. Revised by Boyes, JH. 1970.
391. Warda E, Kamiński J, Modrzewski K. Tumors and tumor-like lesions of the hand. Chirurgia narzadow ruchu i ortopedia polska. 1974 Jan 1;39(6):773-8.
392. Brüchle H, Brüser P, Thiele F. Tumors of the hand. Die Medizinische Welt. 1977;28(7):321-5.
393. Troncoso A, Ro JY, Grignon DJ, Han WS, Wexler H, Von Eschenbach A, Ayala AG. Renal cell carcinoma with acrometastasis: report of two cases and review of the literature. Modern pathology: an official journal of the United States and Canadian Academy of Pathology, Inc. 1991 Jan 1;4(1):66-9.
394. Bibi C, Benmeir P, Maor E, Sagi A. Hand metastasis from renal cell carcinoma with no bone involvement. Annals of plastic surgery. 1993 Oct 1;31(4):377-8.
395. Kierney PC, van Heerden JA, Segura JW, Weaver AL. Surgeon's role in the management of solitary renal cell carcinoma metastases occurring subsequent to initial curative nephrectomy: an institutional review. Annals of surgical oncology. 1994 Jul;1(4):345-52.
396. Sidhu PS, Lewis M, Nicholson DA. Soft tissue metastasis from a renal cell carcinoma. British journal of urology (Print). 1994;74(6):799-801.
397. Giberti C, Mantero R, Schenone M, Costa M, Lavagna M. Unusual location for secondary bone metastasis from advanced renal cell carcinoma. Acta urologica italica. 1999;13(3):177-8.
398. Fusetti C, Kurzen P, Bonaccio M, Büchler U, Nagy L. Hand metastasis in renal cell carcinoma. Urology. 2003 Jul 1;62(1):141.
399. Riter HG, Ghobrial IM. Renal cell carcinoma with acrometastasis and scalp metastasis. Mayo Clin Proc. 2004;79(1):76.
400. Salesi N, Russillo M, Marandino F, Ruggeri EM, Metro G, Cognetti F, Fabi A. Bone of the hands as unusual metastastatic site of renal cell carcinoma. Journal of experimental & clinical cancer research: CR. 2007 Dec 1;26(4):595-7.
401. Pugliese F, Pagliuca V. Images in clinical medicine. Metastasis of renal-cell carcinoma. The New England Journal of Medicine. 2021;265(18):e38.
402. Sonoda LI, Halim MY, Balan KK. Solitary phalangeal metastasis of renal cell carcinoma on bone scintigram. Clinical nuclear medicine. 2011 Mar 1;36(3):237-9.
403. Nakagawa R, Susa M, Nakayama R, Watanabe I, Horiuchi K, Toyama Y, Nakamura M, Matsumoto M, Morioka H. Metastasis of renal cell carcinoma to the trapezium. The Journal of Hand Surgery (Asian-Pacific Volume). 2017 Jun;22(02):262-5.
404. Abro C, Sedhom R, Soni A, Markowski M. Cutaneous finger and tongue metastases in renal cell carcinoma. BMJ Case Rep. 2019 Jun 1;12:e230516.
405. Panaiyadiyan S, Singh P, Nayak B. Acrometastasis to the Hand in Renal Cell Carcinoma. Indian Journal of Surgery. 2020 Oct;83(5):1294-6.
406. Tan DW, Lateef F. Have You Heard of Acrometastasis. J Med Case Rep Case Series. 2020;1(1).
407. De Massary E, Weil P. Carcinose generalisee. Cancers des doigts simulant des troubles trophiques. Bull Soc Med Hopitaux Paris. 1907;24:1456-62.
408. Kovařík V. Metastázy Grawitzova nádoru do prstov. Kovalenko VN: Klinicheskala effektivnost ' Probl Tuberk 1971;49:11-4.
409. Bunkis J, Carter RD. Peripheral bone metastasis from genitourinary tumors. Urology. 1982 Mar 1;19(3):304-5.
410. Radó J, Svastits E, Pánovics J. Kidney cancer causing bone metastasis of unusual localization. Orvosi hetilap. 1982 Mar 1;123(12):735-6.
411. Battistelli JM, Truong P, Nicollet O, Dal Soglio S, Bretagnolle P. Metastase osseuse du pouce sous forme pseudo-angiomateuse. A propos d'un cas. Journal de radiologie (Paris). 1985;66(8-9):551-3.
412. Jebson PJL, Buckwalter JA, Blair WF, Platz CE. Hand metastasis from renal carcinoma. Iowa Orthop J. 1992;12:85-87.
413. Witthaut J, Steffens K, Koob E. An unusual case of metastasis to the soft tissues of the palm of the hand with compression of the median and ulnar nerve by kidney cancer. Case report. Handchirurgie, Mikrochirurgie, Plastische Chirurgie: Organ der Deutschsprachigen Arbeitsgemeinschaft fur Handchirurgie: Organ der Deutschsprachigen Arbeitsgemeinschaft fur Mikrochirurgie der Peripheren Nerven und Gefasse: Organ der V... 1994 May 1;26(3):137-40.
414. Blanes M, Bañuls J, Guijarro J, Betlloch I, Payá A. Metástasis acral digital ósea por adenocarcinoma renal con afectación cutánea. Actas Dermo-Sifiliográficas. 2003 Jan 1;94(8):555-8.
415. Tan KT, Simpson C, Chandrasekar CR. Hand metastasis: an unusual presentation of renal cell carcinoma. Hand. 2012;7(2): 204-206.
416. Hayes MM, Jones EC, Verma AK, Lim CH, Milne G, Tse E. Transitional cell carcinoma of the renal pelvis metastatic to the metacarpal. A case report correlating cytologic and histologic findings. Acta cytologica. 1992 Nov 1;36(6):946-50.
417. Carando M. Su una rara metastasi falangea di carcinoma della vescica. Urologia. 1951;18:256.
418. Martín-Jiménez AJ, Corzo-Gilabert JR, Lidón-Mazón A, García-Gil D. Metastatic phalangeal disease. QJM: An International Journal of Medicine. 2019 Mar 1.
419. Assem H, Broeke N, Coleman L, Gotto G, Bismar TA. Case–Highly aggressive urothelial carcinoma of the bladder presenting with solitary metastasis to the phalanx. Canadian Urological Association Journal. 2020 Nov;14(11):E607.
420. Heymans M, Jardon-Jeghers C, Vanwijck R. Hand metastases from urothelial tumor. The Journal of hand surgery. 1990 May 1;15(3):509-11.
421. Marya SK, Singh SU, Jaswal TS, Garg PR, Gupta AK. Digital and clavicular bone metastasis from transitional cell carcinoma of urinary bladder: a case report. Indian journal of pathology & microbiology. 1993 Jan 1;36(1):75-7.
422. Walsh TM, Mileski RA, Ferlic TP. Metastatic transitional cell carcinoma to the hand. J Hand Surg Am. 1994;19(5):806-808.
423. Taleb C, Pelissier P, Choughri H. Bladder urothelial carcinoma with acrometastasis: a case report and review of the literature. Chirurgie de la Main. 2011 Feb 1;30(2):136-9.
424. Yoneda SU, Kinjo TA, Nonomura DA, Yamamoto Y, Nomura HI, Tei N, Takada S, Matsumiya K, Fujioka HI. A case of bladder cancer with metastasis to the bone of the hand. Hinyokika kiyo. Acta urologica Japonica. 2013 Feb 1;59(2):129-31.
425. Bauer, P., Saalfeld, U., Schmidt, G. and Partecke, B.D., 1997. A rare case of metastasis of urinary bladder carcinoma in the pisiform bone. A case report. Handchirurgie, Mikrochirurgie, Plastische Chirurgie: Organ der Deutschsprachigen Arbeitsgemeinschaft fur Handchirurgie: Organ der Deutschsprachigen Arbeitsgemeinschaft fur Mikrochirurgie der Peripheren Nerven und Gefasse: Organ der V. 1997;29(3):154-157.
426. Turan I, Sjöden GO, Kalen A. Ovarian carcinoma metastasis to the little finger. Acta orthopaedica Scandinavica. 1990 Jan 1;61(2):185-6.
427. Falk DP, Scully R, Moss D, Shaffer M. Pathologic tuft fracture in a thumb: a rare presentation of metastatic endometrioid ovarian carcinoma: a case report and review of the literature. JBJS case connector. 2017 Sep 1;7(3):e50.
428. Riba LW. Testicular tumors: chorionepithelioma with generalized and skin metastases. Surgical Clinics of North America. 1950 Feb 1;30(1):158
429. Gartmann H. Seminommetastasen der haut. Dermatol Wochenschr. 1958;138:828-9.
430. Ornetti P, Favier L, Varbedian O, Ansemant T. Digital acrometastasis revealing endometrial cancer relapse. Arthritis & Rheumatism. 2012 Oct;64(10):3167-.
431. Hetzel DJ, Olt GJ, Sorosky JI, Mortel R, Singapuri K, Podczaski ES. Gestational Trophoblastic Disease Presenting asa Large Metastasis to the Finger. Gynecologic oncology. 1996 Oct 1;63(1):123-6.
432. Afshar A, Ayatollahy H, Lotfinejad S. A rare metastasis in the hand: a case of cutaneous metastasis of choriocarcinoma to the small finger. The Journal of hand surgery. 2007 Mar 1;32(3):393-6.
433. Marek S, Vortel V. Late metastases to the thumb thirteen years after hysterectomy for cancer of the corpus uteri. Rozhl Chir (Prague). 1949;28:272-6.
434. Nobuhara K. Metastatic malignant tumor erroneously diagnosed as felon of the little finger. Seikei-Geka. 1967;18:141-5.
435. Elamurugan TP, Agrawal A, Naskar D, Kate V, Reddy R, Elamurugan S, Basu D. Palmar cutaneous metastasis from carcinoma cervix. Indian Journal of Dermatology, Venereology & Leprology. 2011 Mar 1;77(2).
436. Tully AJ, Shirley SW. A Case of Adenocarcinoma of the Prostate Presenting as Tenosynovitis of the Hand. The Journal of urology. 1972 Jul;108(1):120-2.
437. García-Galaviz R, Domínguez-Cherit J, Caro-Sánchez C, Salazar-Rojas E. Subungual Metastasis of an Adenocarcinoma of the Prostate in a Finger. Skin appendage disorders. 2019;5(1):46-9.
438. Lander RD, O’Donnell MJ. A Case of Metastatic Prostate Cancer to the Distal Phalanx. HAND. 2021 Jul 3:15589447211028922.
439. Ruggiero A, Borri M. Rara localizzazione di metastasi rivelatrici di tumore renale. Nuntius Radiol. 1955;21:209-.
440. Schinz HR, Baensch WE, Friedl E, Uehlinger E. Roentgen-Diagnostics. Translated by JT Case. New York, Grune and Stratton, 1952;2(2):992.
441. Massraf AB, Wand JS. Haemorrhagic secondary prostatic metastasis of the terminal phalanx of the thumb. Injury. 1998 Apr 1;29(3):243-5.
442. Korsten P. Distal extremity metastases from prostate cancer in a patient with rheumatoid arthritis. 2019.
443. Nagano A, Ohno T, Oshima K, Ishimaru D, Nishimoto Y, Ohno Y, Hirakawa A, Miyazaki T, Akiyama H. Metastatic prostate cancer of hand. Case reports in orthopedics. 2016 Oct 23;2016.
444. Gallardo-Alvarado L, Ramos AA, Perez-Montiel D, Ramirez-Morales R, Diaz E, Cantu-de Leon D. Hand metastasis in a patient with cervical cancer: A case report. Medicine. 2020 Jul 2;99(27).
445. Dalicho FH, Beckmann D, Hübner L. Metastasis of cervical cancer to the end phalanx of the small finger. Zentralblatt fur Gynakologie. 1988 Jan 1;110(2):111-4.
446. Pertzborn S, Buekers TE, Sood AK. Hematogenous skin metastases from cervical cancer at primary presentation. Gynecologic oncology. 2000 Mar 1;76(3):416-7.
447. Folasire AM, Michael AI, Oladeji AA, Ogun GO. Bilateral, non-bony metastases to the hand from cervical carcinoma. Annals of Ibadan Postgraduate Medicine. 2019;17(2):187-9.
448. Khosla D, Rai B, Patel FD, Sapkota S, Srinvasan R, Sharma SC. Acrometastasis to hand in vaginal carcinoma: a rare entity. Journal of cancer research and therapeutics. 2012 Jul 1;8(3):430.
449. Miyakawa M, Ito M, Ueyama H, Kuze M. Metastasis of the penile cancer to the skin of thumb--a study on metastasis of the penile cancer. Hinyokika kiyo. Acta urologica Japonica. 1972 Feb 1;18(2):88-96.
450. Cegla P, Konstanty E, Fundowicz M, Pietrasz K, Matuszewski K, Heydrych A, Piotrowski T. First case of acrometastases to the wrist reported from penile cancer. Journal of Cancer Research and Therapeutics. 2021 Apr 1;17(2):599.
451. Gelberman RH, Stewart WR, Harrelson JM. Hand metastasis from melanoma: a case study. Clinical orthopaedics and related research. 1978 Oct 1(136):264-6.
452. Tochigi H, Nakao Y, Horiuchi Y, Toyama Y. Metastatic malignant melanoma in the hand muscle. Hand Surgery. 2000 Jul;5(1):69-72.
453. Xavier T, Conill C, Combalia A, Pomes J, Castel T, Nicolau C. Malignant melanoma with metastasis into the capitate. European journal of radiology. 2005 Dec 1;56(3):362-4.
454. Stahl S, Stahl AS, Lotter O, Pfau M, Perner S, Schaller HE. Palliative surgery for skeletal metastases from melanoma in the scaphoid–A critical case report appraisal. Journal of plastic, reconstructive & aesthetic surgery. 2012 Aug 1;65(8):1111-5.
455. Lambert D, Escallier F, Collet E, Dallac S, Maingon P, Mayer F, Bastien H. Distal phalangeal metastasis of a chondrosarcoma presenting initially as bilateral onycholysis. Clinical and experimental dermatology. 1992 Nov;17(6):463-5.
456. Froimson AI. 17 Metastatic Chondrosarcoma of the Hand Report of a Case. Clinical Orthopaedics and Related Research®. 1967 Jul 1;53:155-60.
457. Ramseier LE, Dumont CE, Ulrich Exner G. Multiple subungual soft tissue metastases from a chondrosarcoma. Scandinavian journal of plastic and reconstructive surgery and hand surgery. 2007 Jan 1;41(6):332-3.
458. Ozcanli H, Oruc F, Aydin AT. Bilateral multiple cutaneous hand metastases of chondrosarcoma. Journal of the European Academy of Dermatology and Venereology: JEADV. 2006 Aug 1;20(7):893-4.
459. Emori M, Kaya M, Sugita S, Soma T, Sasaki M, Yamashita T. Hand metastasis from a sacral chordoma. The Annals of The Royal College of Surgeons of England. 2014 Nov;96(8):e8-11.
460. Smith Z, Girard N, Hansford BG. Multifocal metastatic chordoma to the soft tissues of the fingertips: a case report including sonographic features and a review of the literature. Skeletal radiology. 2018 Mar;47(3):401-6.
461. King DT, Gurevitch AW, Hirose FM. Multiple cutaneous metastases of a scapular chondrosarcoma. Archives of dermatology. 1978 Apr 1;114(4):584-6.
462. Sanjay B, Raj GA, Vishwakarma G. A small-cell osteosarcoma with multiple skeletal metastases. Archives of orthopaedic and traumatic surgery. 1987 Dec;107(1):58-60.
463. Marcove RC, Charosky CB. Phalangeal sarcomas simulating infections of the digits: review of the literature and report of four cases. Clinical Orthopaedics and Related Research®. 1972;83:224-31.
464. Chin KR, Pess GM, Jupiter JB. Chronic lymphocytic leukemia presenting as pyogenic arthritis of the proximal interphalangeal joint. The Journal of hand surgery. 1998 May 1;23(3):545-50
465. Afshar A, Ilkhanizadeh B. Leukaemia cutis of the distal phalanx of the right thumb. Journal of Hand Surgery (European Volume). 2010 Feb;35(2):153-4.
466. Cruz D, Wild T, Glavynskyi I, Weissenberg K, Frenzel S, Florschütz A, Winter J. Unusual Manifestation of Chronic Lymphocytic Leukemia in the Hand. The Journal of hand surgery. 2021 Jan 1;46(1):74-e1.
467. Fino P, Fioramonti P, Onesti MG, Passaretti D, Scuderi N. Skin metastasis in patient with hairy cell leukemia: case report and review of literature. in vivo. 2012 Mar 1;26(2):311-4.
468. Chang YD, Whitaker LA, Larossa D. Acute monomyelocytic leukemia presenting as a felon: Case Report. Plastic and reconstructive surgery. 1975 May 1;55(5):623-4.
469. Roushdi I, Jeswani T, Clark D. Lymphoma presenting as a metastasis to the hand. Journal of hand surgery. European volume. 2012;37(3):286-7.
470. Meyerding HW. Multiple myeloma. Radiology 1925;5:143.
471. Pobanz DM, Condon JV, Baker LA. Plasma-cell myelomatosis: report of a case with multiple large tumors involving the digits of both hands. AMA archives of internal medicine. 1955 Dec 1;96(6):828-32.
472. Cho EY, Kim TH, Park SD, Yun KJ, Choi SC, Kim HC, Nah YH. Acral metastasis in a patient with ampullary carcinoma. The Korean journal of internal medicine. 2007 Mar;22(1):55.
473. Reichert B, Hoch J, Plötz W, Mailänder P, Moubayed P. Metastatic clear-cell sarcoma of the capitate: A case report. JBJS. 2001 Nov 1;83(11):1713-7.
474. Bubau M, Georgiu C, Fodor L. Bilateral Hand Metastases From a Pleomorphic/Dedifferentiated Leiomyosarcoma: A Case Report. The American Journal of Dermatopathology. 2021 Nov 1.
475. Cuadros AV, Riesco MR, Curto CR. Cutaneous acrometastasis from an undifferentiated pleomorphic sarcoma with giant cells. Indian journal of dermatology, venereology and leprology. 2021 Apr 23:1-3.
476. Diflo T, Cantelmo NL, Haudenschild CC, Watkins MT. Atrial myxoma with remote metastasis: case report and review of the literature. Surgery. 1992 Mar 1;111(3):352-6.
477. Frank VH, Pratt Jr CI. A lymphosarcoma with metastasis. Journal of oral surgery. 1951 Jan;9(1):19-24.
478. Chen TJ, Chiou CC, Chen CH, Kuo TT, Hong HS. Metastasis of mediastinal epithelioid angiosarcoma to the finger. American journal of clinical dermatology. 2008 Jun;9(3):181-3.
479. Brownlow HC, Ioannidis G, Gibbons MC, Jones AC, Athanasou N. Digital metastases of giant cell rich malignant fibrous histiocytoma. Sarcoma. 1999 Dec 1;3(3-4):167-70.
480. Chirodian N, Dickson MG, Kerr PS. Fingertip metastasis presenting with a history of trauma. Hospital medicine (London, England: 1998). 1998 Oct 1;59(10):819-.
481. Giordano V, Giordano M, Giordano C, Giordano J, Koch HA, Knackfuss IG. Metastatic tumor of the hand of unknown primary origin. SAGE open medical case reports. 2019 Mar;7:2050313X19836894.
482. Vittali HP. Metastatische tumoren des handskelets. Archiv für orthopädische und Unfall-Chirurgie, mit besonderer Berücksichtigung der Frakturenlehre und der orthopädisch-chirurgischen Technik. 1961 Jul 1;53(4):364-70.
483. Healey JH, Turnbull AD, Miedema B, Lane JM. Acrometastases. A study of twenty-nine patients with osseous involvement of the hands and feet. The Journal of bone and joint surgery. American volume. 1986 Jun 1;68(5):743-6.
484. Leeson MC, Makley JT, Carter JR. Metastatic skeletal disease distal to the elbow and knee. Clinical orthopaedics and related research. 1986 May 1(206):94-9.
485. Amadio PC, Lombardi RM. Metastatic tumors of the hand. The Journal of hand surgery. 1987 Mar 1;12(2):311-6.
486. Libson E, Bloom RA, Husband JE, Stoker DJ. Metastatic tumours of bones of the hand and foot. Skeletal radiology. 1987 Jul;16(5):387-92.
487. Morris G, Evans S, Stevenson J, Kotecha A, Parry M, Jeys L, Grimer R. Bone metastases of the hand. The Annals of The Royal College of Surgeons of England. 2017 Sep;99(7):563-7.
488. El Abiad JM, Aziz K, Levin AS, McCarthy EM, Morris CD. Osseous metastatic disease to the hands and feet. Orthopedics. 2019 Mar 1;42(2):e197-201.
489. Tani S, Morizaki Y, Uehara K, Sawada R, Kobayashi H, Shinoda Y, Kawano H, Tanaka S. Bone metastasis of limb segments: Is mesometastasis another poor prognostic factor of cancer patients?. Japanese journal of clinical oncology. 2020 Jun;50(6):688-92.
