# Supplementary material for: Cancer Metastases to the Hand: A Systematic Review and Meta-analysis
Source: Hand (N Y). 2023 Mar 1;19(6):865–74. doi: 10.1177/15589447231153175 (PMC11342693; doi:10.1177/15589447231153175)
Supplement: sj-docx-3-han-10.1177_15589447231153175 – Supplemental material for Cancer Metastases to the Hand: A Systematic Review and Meta-analysis [file sj-docx-3-han-10.1177_15589447231153175.docx]

| **Author** | **Year** | **Age/ Sex** | **Site of Primary Cancer** | **Cancer Cell Type** | **Site of Acrometastasis** | **Known History of Malignancy** | **Acrometastasis Treatment** | **Survival** |
| --- | --- | --- | --- | --- | --- | --- | --- | --- |
| **Cancers of the Head and Neck** | | | | | | | | |
| Komminoth^A1^ | 1977 | 52/M | Oral Cancer | Squamous cell carcinoma | Left 5DP | Yes | Amputation/ Excision | NM |
| Isomura et al.^A2^ | 2020 | 65/M | Oral Cancer (Buccal Floor) | Squamous cell carcinoma | Right 3DP | Yes | NM | 2 Months |
| Mohanty et al.^A3^ | 2018 | 40/M | Oral Cancer (Gingivobuccal sulcus) | Squamous cell carcinoma | Right 3MP | Yes | Amputation/ Excision | 2 Months |
| Fragiadakis & Panayotopoulos^A4^ | 1972 | 60/M | Oral Cancer (Hard Palate) | Squamous cell carcinoma | Left 4MC, 5MC, 4PP, 5PP | Yes | Amputation/ Excision | Alive At Time of Publication |
| Castello et al.^A5^ | 1996 | 68/M | Oral Cancer (Hard Palate) | Squamous cell carcinoma | Right 1DP | No | Amputation/ Excision | 10 Months |
| Viswanathan et al.^A6^ | 1996 | 70/M | Oral Cancer (Lower alveolus) | Squamous cell carcinoma | Right 2PP, 2MP, 2DP, 3DP | Yes | Radiation | NM |
| Shrivastava et al.^A7^ | 2009 | 66/M | Oral Cancer (Lower alveolus) | Squamous cell carcinoma | Left 3MP | Yes | Radiation | 3 Months |
| Sandberg et al.^A8^ | 2015 | 53/F | Oral Cancer (Oropharyngeal) | Squamous cell carcinoma | Left 2MP | Yes | Conservative/ Palliative Treatment | NM |
| Trehan et al.^A9^ | 2015 | 55/M | Oral Cancer (Supraglotic) | Squamous cell carcinoma | Right Dorsum of Hand (ST) | Yes | Radiation | NM |
| Castigliano^A10^ | 1966 | 65/M | Oral Cancer (Tongue) | Squamous cell carcinoma | Left 1DP | Yes | Amputation/ Excision | 2 Months |
| Vanel^A11^ | 1990 | 68/NM | Oral Cancer (Tongue) | Squamous cell carcinoma | Right 2DP | NM | NM | NM |
| Vanel^A11^ | 1990 | 72/M | Oral Cancer (Tongue) | Squamous cell carcinoma | Left 2DP | NM | NM | NM |
| Longo et al.^A12^ | 2007 | 66/M | Oral Cancer (Tongue) | Squamous cell carcinoma | Left Pulp D5 (ST) | Yes | Conservative/ Palliative Treatment | 2 Months |
| Aiempanakit^A13^ | 2018 | 61/M | Oral Cancer (Tongue) | Squamous cell carcinoma | Left Subungal D2 (ST) | Yes | Conservative/ Palliative Treatment | 0.25 Months |
| Mandadi & Kudva^A14^ | 2018 | 66/M | Oral Cancer (Tongue) | Squamous cell carcinoma | Right 5DP | Yes | Amputation/ Excision | NM |
| Joll^A15^ | 1923 | 45/M | Nasopharyngeal Cancer | NM | NS "Phalanx", "Metacarpals" | Yes | NM | NM |
| Plotkine et al.^A16^ | 2008 | 80/M | Nasopharyngeal Cancer | NM | Right 3DP | Yes | Amputation/ Excision | Alive At Time of Publication |
| TaeSooChung^A17^ | 1983 | 56/M | Nasopharyngeal Cancer | Plasmocytoma | Left 1DP | Yes | Radiation | Alive At Time of Publication |
| Sur et al.^A18^ | 2011 | 62/F | Nasopharyngeal Cancer | Plasmocytoma | Left 5MP,  Right 1DP | Yes | Amputation/ Excision | 10 Months |
| Gold & Reefe^A19^ | 1963 | 73/M | Nasopharyngeal Cancer | Squamous cell carcinoma | Left 3PP | No | NM | 1.7 Months |
| Castello et al.^A5^ | 1996 | 67/M | Nasopharyngeal Cancer | Squamous cell carcinoma | Right Wrist (ST) | Yes | Amputation/ Excision | 8 Months |
| Sipahioglu et al.^A20^ | 2012 | 60/M | Nasopharyngeal Cancer | Squamous cell carcinoma | NS Pulp D3 (ST) | Yes | Amputation/ Excision | 24 Months |
| Filloux & Fontaine^A21^ | 2000 | 54/M | Pharyngeal Cancer | NM | Right Pulp D5 (ST) | Yes | Amputation/ Excision | 3 Months |
| Pirschel et al.^A22^ | 1978 | 65/F | Pharyngeal Cancer | Transitional cell | Left "Diffuse Bones",  Right "Diffuse Bones" | No | Radiation | NM |
| Bazex et al.^A23^ | 1968 | 57/M | Laryngeal Cancer | Squamous cell carcinoma | Left 3DP,  Right 3DP | Yes | NM | NM |
| Cohen & Laszlo^A24^ | 1972 | 58/M | Laryngeal Cancer | Squamous cell carcinoma | Left Trapezium, Scaphoid, 1MC, 2MC | Yes | Conservative/ Palliative Treatment | 1 Month |
| Uriburu et al.^A25^ | 1976 | 65/M | Laryngeal Cancer | Squamous cell carcinoma | Left 3PP | Yes | Amputation/ Excision | 4 Months |
| Mess & Sanger^A26^ | 1986 | 55/M | Laryngeal Cancer | Squamous cell carcinoma | Right 2PP | Yes | Amputation/ Excision | 3 Months |
| Castello et al.^A5^ | 1996 | 65/M | Laryngeal Cancer | Squamous cell carcinoma | Right 2DP | Yes | Amputation/ Excision | 6 Months |
| Lewin et al.^A27^ | 1997 | 70/M | Laryngeal Cancer | Squamous cell carcinoma | Left Pulp D1 (ST) | Yes | NM | 1.5 Months |
| Filloux & Fontaine^a21^ | 2000 | 58/M | Laryngeal Cancer | Squamous cell carcinoma | Left Volar 3MP (ST) | Yes | Amputation/ Excision | 14 Months |
| Aydin et al.^A28^ | 2004 | 61/M | Laryngeal Cancer | Squamous cell carcinoma | Left Volar 5PP (ST) | Yes | Amputation/ Excision | 3 Months |
| Ottomani et al.^A29^ | 2008 | 52/M | Laryngeal Cancer | Squamous cell carcinoma | Left Pulp D2 (ST),  Right Pulp D3 (ST), Subungal D3 (ST) | Yes | Amputation/ Excision | 9 Months |
| Kumar et al.^A30^ | 2011 | 55/M | Laryngeal Cancer | Squamous cell carcinoma | Left 1DP, 2DP, 3DP, 4DP, 5DP | Yes | Conservative/ Palliative Treatment | NM |
| Shetty et al.^A31^ | 2016 | 55/M | Pyriform sinus | Squamous cell carcinoma | Left 1DP, 4DP | Yes | NM | NM |
| Elvey et al.^A32^ | 2011 | 67/M | Parotid Gland | Basal Cell Adenocarcinoma | Left 2MC, 3MC, 4MC, 5MC | Yes | Amputation/ Excision | Alive At Time of Publication |
| Esther & Bos^A33^ | 2000 | 58/F | Parotid Gland | Mucoepidermoid Carcinoma | Right Capitate | Yes | Radiation | Alive At Time of Publication |
| Mason^A34^ | 1937 | 41/F | Parotid Gland | NM | Right "DP" | NM | NM | NM |
| Ariel et al.^A35^ | 1954 | NM/F | Parotid Gland | NM | Right Subungal D3 (ST) | Yes | NM | "Expired" |
| Falkinburg & Fagan^A36^ | 1956 | 68/F | Parotid Gland | Squamous cell carcinoma | Left Pulp D3 (ST) | Yes | Amputation/ Excision | 12 Months |
| Vinod & Gay^A37^ | 1979 | 47/M | Salivary Gland Cancer | adenoid cystic carcinoma | Right Palm (ST) | Yes | NM | NM |
| **Thyroid Cancers** | | | | | | | | |
| Uriburu et al.^A25^ | 1976 | 64/F | Thyroid Cancer | Follicular Carcinoma | Left 4MP | Yes | Amputation/ Excision | Alive At Time of Publication |
| Horn^A38^ | 1982 | 50/F | Thyroid Cancer | Follicular Carcinoma | Left 3PP | Yes | Radioiodine | 3 Months |
| Reparaz Padros et al.^A39^ | 2006 | 61/F | Thyroid Cancer | Follicular Carcinoma | Left 4DP | Yes | Amputation/ Excision | NM |
| Huri^A40^ | 2011 | 84/M | Thyroid Cancer | Follicular Carcinoma | Right 1DP | Yes | Amputation/ Excision | NM |
| Krishnamurthy & Ramshankar^A41^ | 2013 | 45/M | Thyroid Cancer | Follicular Carcinoma | Left 3MC | No | Radiation | Alive At Time of Publication |
| Kattepur & Gopinath^A42^ | 2014 | 50/F | Thyroid Cancer | Follicular Carcinoma | Left 5DP | No | Conservative/ Palliative Treatment | Alive At Time of Publication |
| Patil & Kumaraswamy Kattepur^A43^ | 2014 | 54/F | Thyroid Cancer | Follicular Carcinoma | Left 5DP | No | Radioiodine | Alive At Time of Publication |
| Chakravarthy et al.^A44^ | 2010 | 32/F | Thyroid Cancer | Papillary Carcinoma | Right 3MC, 4MC, 5MC | No | NM | NM |
| Shannon et al.^A45^ | 2000 | 54/M | Thyroid Cancer | Undifferentiated | Right 4DP | Yes | Amputation/ Excision | 2 Months |
| **Lung Cancers** | | | | | | | | |
| Rendich & Levy^A46^ | 1941 | 40/M | Lung Cancer | Adenocarcinoma | Right 5PP | Yes | Conservative/ Palliative Treatment | 6 Months |
| Greene^A47^ | 1957 | 34/M | Lung Cancer | Adenocarcinoma | Right 3PP | Yes | Amputation/ Excision | 8 Months |
| Smith^A48^ | 1963 | 41/M | Lung Cancer | Adenocarcinoma | Right Trapezoid | No | Radiation | 2 Months |
| Hicks et al.^A49^ | 1964 | 43/M | Lung Cancer | Adenocarcinoma | Right 2PP | Yes | Declined Treatment | 21 Months |
| Mulvey^A50^ | 1964 | 68/F | Lung Cancer | Adenocarcinoma | Left 2PP | Yes | NM | NM |
| Taddei & Pistocchi^A51^ | 1965 | 63/F | Lung Cancer | Adenocarcinoma | Left 2DP | Yes | Radiation | 4 Months |
| Mohanty et al.^A52^ | 1968 | 57/M | Lung Cancer | Adenocarcinoma | Left 4DP | No | Amputation/ Excision | 8 Months |
| Tondeur et al.^A53^ | 1969 | 61/M | Lung Cancer | Adenocarcinoma | Left 3PP, 3MC | No | Amputation/ Excision | 4 Months |
| Fam & Cross^A54^ | 1979 | 61/F | Lung Cancer | Adenocarcinoma | Right 1PP, 3PP | Yes | Conservative/ Palliative Treatment | 6 Months |
| Saitoh et al.^A55^ | 1983 | 57/M | Lung Cancer | Adenocarcinoma | Right 1DP | Yes | Radiation | NM |
| Rochet et al.^A56^ | 1991 | 62/M | Lung Cancer | Adenocarcinoma | Left 1DP | No | Amputation/ Excision + Radiation | NM |
| Rousseau et al.^A57^ | 1992 | 72/M | Lung Cancer | Adenocarcinoma | Left Volar 5PP (ST) | Yes | Amputation/ Excision | 2 Months |
| Roncaglio & Arena^A58^ | 1993 | 52/M | Lung Cancer | Adenocarcinoma | Left Lunate | No | NM | 4 Months |
| Knapp & Abdul-Karim^A59^ | 1994 | 89/F | Lung Cancer | Adenocarcinoma | Right 2MP | No | Radiation | 4 Months |
| Abrahams^A60^ | 1995 | 62/M | Lung Cancer | Adenocarcinoma | Right 1PP | No | Radiation | NM |
| Javed et al.^A61^ | 1997 | 72/F | Lung Cancer | Adenocarcinoma | Right 2DP | No | Amputation/ Excision | "Expired" |
| Galmarini et al.^A62^ | 1998 | 53/M | Lung Cancer | Adenocarcinoma | Left 1DP | No | Amputation/ Excision | 2 Months |
| Matsuno et al.^A63^ | 2002 | 70/M | Lung Cancer | Adenocarcinoma | Right Thenar Emenience (ST) | Yes | Conservative/ Palliative Treatment | 3 Months |
| Raissouni et al.^A64^ | 2002 | 36/F | Lung Cancer | Adenocarcinoma | Right 3MC | No | Conservative/ Palliative Treatment | 0.75 Months |
| Afifi & Perez^A65^ | 2004 | 70/M | Lung Cancer | Adenocarcinoma | Left 5DP | No | Amputation/ Excision | 4 Months |
| Campa et al.^A66^ | 2004 | 72/F | Lung Cancer | Adenocarcinoma | Right 4DP | Yes | Amputation/ Excision | 6 Months |
| Keramidas & Brotherston^A67^ | 2005 | 66/F | Lung Cancer | Adenocarcinoma | Right Capitate, Hamate, 3MC, 4MC, 5MC | No | Referred to Oncology | 3 Months |
| Akjouj et al.^A68^ | 2006 | 57/M | Lung Cancer | Adenocarcinoma | Left 1DP | No | Amputation/ Excision | 3 Months |
| Han et al.^A69^ | 2007 | 56/M | Lung Cancer | Adenocarcinoma | NS 5DP | No | Amputation/ Excision | Alive At Time of Publication |
| Preto et al.^A70^ | 2007 | 61/M | Lung Cancer | Adenocarcinoma | Right Trapezoid, 1MC, 2MC | No | NM | 6 Months |
| Ahlmann et al.^A71^ | 2008 | 62/F | Lung Cancer | Adenocarcinoma | Left 1PP, 1DP, Pulp D1 (ST) | Yes | Amputation/ Excision | Alive At Time of Publication |
| Ahlmann et al.^A71^ | 2008 | 65/M | Lung Cancer | Adenocarcinoma | Left Hamate | No | Radiation | 12 Months |
| Ahlmann et al.^A71^ | 2008 | 73/M | Lung Cancer | Adenocarcinoma | Left 4PP | No | Amputation/ Excision | 3 Months |
| Seth et al.^A72^ | 2008 | 46/M | Lung Cancer | Adenocarcinoma | Right Subungal D4 (ST) | No | NM | 4 Months |
| Jakhar et al.^A73^ | 2009 | 70/M | Lung Cancer | Adenocarcinoma | Right 4PP | No | Radiation | NM |
| Kodama et al.^A74^ | 2009 | 78/M | Lung Cancer | Adenocarcinoma | Left Hamate | Yes | Radiation | 22 Months |
| Myrehaug & Bezjak^A75^ | 2009 | "Middle aged"/M | Lung Cancer | Adenocarcinoma | Right 2PP | Yes | Radiation | NM |
| Long et al.^A76^ | 2010 | 53/M | Lung Cancer | Adenocarcinoma | Left 3DP | No | Conservative/ Palliative Treatment | 1 Month |
| Stathopoulos & Rigatos^A77^ | 2010 | 35/M | Lung Cancer | Adenocarcinoma | Right 1DP | Yes | Amputation/ Excision | NM |
| Amar et al.^A78^ | 2011 | 40/F | Lung Cancer | Adenocarcinoma | Left 1DP | No | Amputation/ Excision | 11 Months |
| Bhandari & Brown^A79^ | 2011 | 52/M | Lung Cancer | Adenocarcinoma | Left 2PP | No | Amputation/ Excision | NM |
| Huang et al.^A80^ | 2011 | 46/M | Lung Cancer | Adenocarcinoma | Right 5DP | Yes | Conservative/ Palliative Treatment | Alive At Time of Publication |
| Sasaki et al.^A81^ | 2011 | 67/F | Lung Cancer | Adenocarcinoma | Left Dorsum of Hand (ST) | Yes | Conservative/ Palliative Treatment | 9 Months |
| van Uden & Kolkman^A82^ | 2011 | 75/M | Lung Cancer | Adenocarcinoma | Left 2MP | No | Amputation/ Excision + Radiation | "Expired" |
| Ferraro & Lucero^A83^ | 2012 | 52/F | Lung Cancer | Adenocarcinoma | Left 1PP | No | NM | 1.5 Months |
| Gilardi et al.^A84^ | 2013 | 55/M | Lung Cancer | Adenocarcinoma | Left Trapezium | No | Radiation | Alive At Time of Publication |
| Khmamouche et al.^A85^ | 2013 | 72/M | Lung Cancer | Adenocarcinoma | Left 1DP | No | Amputation/ Excision | 1 Month |
| Shaw et al.^A86^ | 2013 | 71/F | Lung Cancer | Adenocarcinoma | Right 1DP | No | Amputation/ Excision + Radiation | 2 Months |
| Liu et al.^A87^ | 2014 | 53/F | Lung Cancer | Adenocarcinoma | Left 2MC | No | Amputation/ Excision | Alive At Time of Publication |
| Poh et al.^A88^ | 2014 | 51/F | Lung Cancer | Adenocarcinoma | Right 5MC | No | Amputation/ Excision | Alive At Time of Publication |
| Sumodhee et al.^A89^ | 2014 | 61/M | Lung Cancer | Adenocarcinoma | Left 4PP | No | Radiation | Alive At Time of Publication |
| van Veenendaal et al.^A90^ | 2014 | 83/F | Lung Cancer | Adenocarcinoma | Right 3PP | Yes | Amputation/ Excision | NM |
| Gorospe Sarasúa et al.^A91^ | 2015 | 43/F | Lung Cancer | Adenocarcinoma | Left 4MP | No | NM | NM |
| Reynolds & Skandan^A92^ | 2016 | 71/F | Lung Cancer | Adenocarcinoma | Left 2DP | No | Amputation/ Excision | 2 Months |
| Feng et al.^A93^ | 2017 | 65/F | Lung Cancer | Adenocarcinoma | Right 2MC | Yes | Radiation | Alive At Time of Publication |
| Xie & Huang^A94^ | 2017 | 45/M | Lung Cancer | Adenocarcinoma | Left 1PP | Yes | Amputation/ Excision | NM |
| El Idrissi et al.^A95^ | 2018 | 68/M | Lung Cancer | Adenocarcinoma | Right 5MC | No | NM | NM |
| Millrose et al.^A96^ | 2018 | 50/M | Lung Cancer | Adenocarcinoma | Left 4PP | Yes | Amputation/ Excision | Alive At Time of Publication |
| Afrăsânie et al.^A97^ | 2019 | 58/M | Lung Cancer | Adenocarcinoma | Right 2DP | No | Amputation/ Excision | 27 Months |
| Machado & San-Julian^A98^ | 2019 | 35/M | Lung Cancer | Adenocarcinoma | Left 1MC | No | Amputation/ Excision | NM |
| Clery et al.^A99^ | 2020 | 69/F | Lung Cancer | Adenocarcinoma | Right 2MP, 2DP | No | Amputation/ Excision | NM |
| Kalakech et al.^A100^ | 2021 | 56/M | Lung Cancer | Adenocarcinoma | Left 4DP | No | Chemotherapy/ Immunotherapy | Poor Prognosis |
| Umana et al.^A101^ | 2021 | 72/F | Lung Cancer | Adenocarcinoma | Right 4DP | No | Amputation/ Excision | Alive At Time of Publication |
| Ross & Fodden^A102^ | 1949 | 59/M | Lung Cancer | Large Cell Carcinoma | Right 1PP | No | Amputation/ Excision | "Expired" |
| Midell & Lochman^A103^ | 1972 | 62/M | Lung Cancer | Large Cell Carcinoma | Left "wrist bones" | Yes | Radiation | 0.25 Months |
| Nissenbaum et al.^A104^ | 1978 | 46/M | Lung Cancer | Large Cell Carcinoma | Right Hamate | No | Amputation/ Excision | 4 Months |
| Letanche et al.^A105^ | 1990 | 53/M | Lung Cancer | Large Cell Carcinoma | Right 5MC | No | Chemotherapy/ Immunotherapy | 2 Months |
| De Abaffy et al.^A106^ | 1998 | 54/M | Lung Cancer | Large Cell Carcinoma | Right 5PP | Yes | Amputation/ Excision | Alive At Time of Publication |
| Rinonapoli et al.^A107^ | 2012 | 74/M | Lung Cancer | Large Cell Carcinoma | Left Trapezium, Trapezoid, Scaphoid | No | Amputation/ Excision | Poor Prognosis |
| Koyama & Koizumi^A108^ | 2014 | 62/M | Lung Cancer | Large Cell Carcinoma | Right Hamate | Yes | NM | NM |
| Strooker et al.^A109^ | 2015 | 83/M | Lung Cancer | Large Cell Carcinoma | Left Lunate | No | Amputation/ Excision | 2 Months |
| Berthier et al.^A110^ | 2020 | 61/M | Lung Cancer | Large Cell Carcinoma | Left 4DP | Yes | Amputation/ Excision | NM |
| Graham et al.^A111^ | 2009 | 66/M | Lung Cancer | Leiomyosarcoma | Left Volar 4MP (ST) | Yes | Amputation/ Excision | "Expired" |
| Bonvoisin et al.^A112^ | 1981 | 75/M | Lung Cancer | Malphighian carcinoma | Right 2DP | No | Amputation/ Excision | 2 Months |
| Asencio et al.^A113^ | 1982 | 66/M | Lung Cancer | Malphighian carcinoma | Left 2DP,  Right 3DP | No | Conservative/ Palliative Treatment | 1.5 Months |
| Asencio et al.^A113^ | 1982 | 68/M | Lung Cancer | Malphighian carcinoma | Left Trapezium, 1MC, 2MC | Yes | Amputation/ Excision | 4 Months |
| Dyck^A114^ | 1965 | 58/M | Lung Cancer | Mesothelioma | Right 5MC | No | Conservative/ Palliative Treatment | 5 Months |
| Kanbay et al.^A115^ | 2007 | 47/M | Lung Cancer | Mesothelioma | Left Pulp D2 (ST) | Yes | Conservative/ Palliative Treatment | 6 Months |
| Celik et al.^A116^ | 1998 | 36/M | Lung Cancer | Mixed Squamous and Small Cell Carcinoma | Left 2DP, 3DP | No | Radiation | 4 Months |
| Floridis et al.^A117^ | 1934 | 47/M | Lung Cancer | NM | Left 3MP | No | Amputation/ Excision | 4 Months |
| Brahdy & Kahn^A118^ | 1941 | NM/F | Lung Cancer | NM | NS "Finger" | No | NM | NM |
| Brailsford^A119^ | 1953 | 60/M | Lung Cancer | NM | Right Trapezoid, Capitate, 2MC | No | NM | NM |
| Reboul et al.^A120^ | 1960 | NM/NM | Lung Cancer | NM | Left 5MC | NM | NM | NM |
| Vancura et al.^A121^ | 1960 | 53/M | Lung Cancer | NM | Right 2MP | Yes | Radiation | 5 Months |
| Vancura et al.^A121^ | 1960 | 64/M | Lung Cancer | NM | Left Scaphoid | Yes | Radiation | 10 Months |
| Amalric et al.^A122^ | 1966 | NM/NM | Lung Cancer | NM | Left Hamate | NM | NM | NM |
| Patryn^A123^ | 1967 | 62/M | Lung Cancer | NM | Left 4MC, 5MC | No | Declined Treatment | NM |
| Dolich et al.^A124^ | 1970 | 69/F | Lung Cancer | NM | Right Trapezoid, Capitate, Hamate, Scaphoid, Lunate, Triquetrum, Pisiform | No | Conservative/ Palliative Treatment | 1.5 Months |
| Hammer & Gollmann^A125^ | 1972 | 46/M | Lung Cancer | NM | Left 2MP | No | Amputation/ Excision | 50 Months |
| Hammer & Gollmann^A125^ | 1972 | NM/M | Lung Cancer | NM | Left 3MP | No | Radiation | Alive At Time of Publication |
| Rolle & Berner^A126^ | 1974 | NM/NM | Lung Cancer | NM | Right 1PP | Yes | Conservative/ Palliative Treatment | NM |
| Pantoja et al.^A127^ | 1976 | 52/M | Lung Cancer | NM | Left 5DP,  Right 1DP, 4DP, 5DP | NM | Amputation/ Excision + Radiation | NM |
| Pantoja et al.^A127^ | 1976 | 68/M | Lung Cancer | NM | Right 1DP | NM | Conservative/ Palliative Treatment | NM |
| Bricout^A128^ | 1981 | 50/F | Lung Cancer | NM | Right 3PP | Yes | Radiation | 1.5 Months |
| Bricout^A128^ | 1981 | 63/M | Lung Cancer | NM | Right 3PP, 1MC | Yes | Radiation | Lost to Follow-up |
| Cary et al.^A129^ | 1981 | 54/M | Lung Cancer | NM | Left Trapezium, Capitate | Yes | NM | NM |
| Cross^A130^ | 1985 | 74/M | Lung Cancer | NM | Right 1DP | No | Amputation/ Excision | 4 Months |
| Morris & House^A131^ | 1985 | 46/M | Lung Cancer | NM | Right 2MC | NM | NM | 2 Months |
| Morris & House^A131^ | 1985 | 71/M | Lung Cancer | NM | Left 1MC | NM | NM | 3 Months |
| Lederer et al.^A132^ | 1990 | 51/M | Lung Cancer | NM | Right Trapezium, Trapezoid | Yes | Amputation/ Excision | NM |
| Castello et al.^A5^ | 1996 | 68/F | Lung Cancer | NM | Right 2DP | Yes | Amputation/ Excision | 7 Months |
| Filloux & Fontaine^A21^ | 2000 | 67/M | Lung Cancer | NM | Right Pulp D2 (ST) | Yes | Amputation/ Excision | 1 Month |
| Filloux & Fontaine^A21^ | 2000 | 72/M | Lung Cancer | NM | Left Pulp D4 (ST) | No | Amputation/ Excision | 6 Months |
| Heidarpour et al.^A133^ | 2006 | 78/F | Lung Cancer | NM | Left 2DP | No | Conservative/ Palliative Treatment | 2 Months |
| Tzaveas et al.^A134^ | 2008 | 68/M | Lung Cancer | NM | Right 5MC | Yes | Referred to Oncology | NM |
| Bowles & Wells^A135^ | 2011 | 63/M | Lung Cancer | NM | Right 4DP | No | Amputation/ Excision | NM |
| Sur et al.^A18^ | 2011 | 54/M | Lung Cancer | NM | NS "PP" | NM | NM | 4 Months |
| Sur et al.^A18^ | 2011 | 69/F | Lung Cancer | NM | NS "PP" | NM | Amputation/ Excision | 6 Months |
| Cavit et al.^A136^ | 2018 | 43/M | Lung Cancer | NM | Left 1PP | NM | NM | NM |
| Tabrizi et al.^A137^ | 2019 | 60/M | Lung Cancer | NM | Left Hamate | No | Amputation/ Excision | 20 Months |
| Parungao & Milner^A138^ | 2002 | 65/M | Lung Cancer | Non-small Cell Carcinoma | Right 1DP | No | Amputation/ Excision | NM |
| Alkhayat & Hong^A139^ | 2006 | 72/F | Lung Cancer | Non-small Cell Carcinoma | Left 4DP | No | Conservative/ Palliative Treatment | 6 Months |
| Bülbül et al.^A140^ | 2006 | 68/M | Lung Cancer | Non-small Cell Carcinoma | Right 5MP | No | Conservative/ Palliative Treatment | 8 Months |
| Marcos Sanchez et al.^A141^ | 2006 | 54/M | Lung Cancer | Non-small Cell Carcinoma | Right 5DP | No | Conservative/ Palliative Treatment | 0.5 Months |
| Flynn et al.^A142^ | 2008 | 78/F | Lung Cancer | Non-small Cell Carcinoma | Left 2MC | No | Radiation | Alive At Time of Publication |
| Gaston et al.^A143^ | 2008 | "40's"/F | Lung Cancer | Non-small Cell Carcinoma | Right Trapezium | Yes | Amputation/ Excision | Poor Prognosis |
| Kontogeorgakos et al.^A144^ | 2011 | 75/F | Lung Cancer | Non-small Cell Carcinoma | Right 3DP | Yes | Amputation/ Excision | 15 Months |
| Song & Yao^A145^ | 2012 | 70/M | Lung Cancer | Non-small Cell Carcinoma | Right Trapezium | No | Conservative/ Palliative Treatment | Poor Prognosis |
| Gjorup et al.^A146^ | 2017 | 55/F | Lung Cancer | Non-small Cell Carcinoma | Right 3MP | No | Amputation/ Excision | 2 Months |
| Baños-Arévalo et al.^A147^ | 2018 | 77/M | Lung Cancer | Non-small Cell Carcinoma | Right "D5" | No | Amputation/ Excision | "Expired" |
| White & Sampson^A148^ | 2021 | 60/F | Lung Cancer | Non-small Cell Carcinoma | Left Pisiform | No | Conservative/ Palliative Treatment | "Expired" |
| Colson & Willcox^A149^ | 1948 | 65/F | Lung Cancer | Small Cell Carcinoma | Left 1DP, 4DP,  Right 1DP | Yes | NM | 1 Month |
| Bell & Mason^A150^ | 1953 | 45/M | Lung Cancer | Small Cell Carcinoma | Left 1DP | Yes | Conservative/ Palliative Treatment | 1 Month |
| Stock^A151^ | 1977 | 68/M | Lung Cancer | Small Cell Carcinoma | Right 3PP | No | Amputation/ Excision | 3 Months |
| Bunkis et al.^A152^ | 1980 | 54/M | Lung Cancer | Small Cell Carcinoma | Right 4DP | Yes | Radiation | 4 Months |
| Józsa & Renner^A153^ | 1991 | 73/M | Lung Cancer | Small Cell Carcinoma | NS Dorsum of Hand (ST) | NM | NM | NM |
| Kolomiets & Lytkin^A154^ | 1991 | 50/M | Lung Cancer | Small Cell Carcinoma | Left 2PP | No | Amputation/ Excision | NM |
| Königsberger & Goth^A155^ | 1996 | 54/M | Lung Cancer | Small Cell Carcinoma | Right Hamate | No | Radiation | 16 Months |
| Mehta & Mehta^A156^ | 2001 | 61/F | Lung Cancer | Small Cell Carcinoma | Right Subungal D1 (ST) | Yes | Amputation/ Excision | 4 Months |
| Carvalho Hde et al.^A157^ | 2002 | 51/F | Lung Cancer | Small Cell Carcinoma | Right 1DP | Yes | Radiation | 10 Months |
| Anandan et al.^A158^ | 2010 | 64/M | Lung Cancer | Small Cell Carcinoma | Left 3PP | Yes | Radiation | "Expired" |
| Ragois et al.^A159^ | 2012 | 72/M | Lung Cancer | Small Cell Carcinoma | Left Hypothenar Emenience (ST) | Yes | Amputation/ Excision | 2 Months |
| Hinterstoisser^A160^ | 1889 | 59/M | Lung Cancer | Squamous cell carcinoma | Right 4DP | No | Amputation/ Excision | 9 Months |
| Froboese^A161^ | 1941 | 48/M | Lung Cancer | Squamous cell carcinoma | Right 3DP | Yes | Amputation/ Excision | 2 Months |
| Smithers & Price^A162^ | 1945 | 49/M | Lung Cancer | Squamous cell carcinoma | Left 5DP | Yes | Amputation/ Excision + Radiation | Alive At Time of Publication |
| Colson & Willcox^A149^ | 1948 | 55/M | Lung Cancer | Squamous cell carcinoma | Left 1DP | No | NM | 2 Months |
| Preissner^A163^ | 1948 | 60/M | Lung Cancer | Squamous cell carcinoma | Right 2PP | No | Amputation/ Excision | 7 Months |
| Brason et al.^A164^ | 1951 | 60/M | Lung Cancer | Squamous cell carcinoma | Left 3DP | No | Amputation/ Excision | 3 Months |
| Freni & Averill^A165^ | 1952 | 58/M | Lung Cancer | Squamous cell carcinoma | Left 1DP | Yes | Amputation/ Excision | 1 Month |
| Strang^A166^ | 1952 | 66/M | Lung Cancer | Squamous cell carcinoma | Left 4MP | No | Amputation/ Excision | 4 Months |
| De Pass et al.^A167^ | 1958 | 50/M | Lung Cancer | Squamous cell carcinoma | Left 1MC | No | Amputation/ Excision | Alive At Time of Publication |
| De Pass et al.^A167^ | 1958 | 58/M | Lung Cancer | Squamous cell carcinoma | Left 1PP | Yes | Radiation | Alive At Time of Publication |
| Kerin^A168^ | 1958 | 47/M | Lung Cancer | Squamous cell carcinoma | Right 2DP | No | Amputation/ Excision | 6 Months |
| Kerin^A168^ | 1958 | 58/M | Lung Cancer | Squamous cell carcinoma | Left 1MC | No | Amputation/ Excision | 7 Months |
| Kerin^A168^ | 1958 | 58/M | Lung Cancer | Squamous cell carcinoma | Left 1DP | Yes | Amputation/ Excision | 2 Months |
| Marmor & Horner^A169^ | 1959 | 49/M | Lung Cancer | Squamous cell carcinoma | Left 3DP | Yes | Amputation/ Excision | 2 Months |
| Pfeiffer^A170^ | 1959 | 52/F | Lung Cancer | Squamous cell carcinoma | Right 3MP | Yes | Amputation/ Excision | 4 Months |
| Kolář et al.^A171^ | 1960 | 72/M | Lung Cancer | Squamous cell carcinoma | Left 1DP, 2DP, 3DP,  Right 1DP, 2DP, 3PP, 3DP | No | Radiation | 4 Months |
| Vancura et al.^A121^ | 1960 | 72/M | Lung Cancer | Squamous cell carcinoma | Right 2DP, 3PP, 3DP | Yes | Radiation | 2 Months |
| Trachtenberg & Roswit^A172^ | 1961 | 65/M | Lung Cancer | Squamous cell carcinoma | Left 3MP | No | Amputation/ Excision | 3 Months |
| Trachtenberg & Roswit^A172^ | 1961 | 66/M | Lung Cancer | Squamous cell carcinoma | Right 5DP | Yes | Amputation/ Excision | 2 Months |
| Karten & Bartfeld^A173^ | 1962 | 59/M | Lung Cancer | Squamous cell carcinoma | Left 1PP, 3MP, 3DP,  Right "D3", "D4", "D5" | No | Conservative/ Palliative Treatment | 1.25 Months |
| Ferguson et al.^A174^ | 1963 | 48/F | Lung Cancer | Squamous cell carcinoma | Left 1DP, Pulp D1 (ST),  Right 4DP, Pulp D4 (ST) | Yes | Amputation/ Excision | NM |
| Bailey^A175^ | 1964 | 64/M | Lung Cancer | Squamous cell carcinoma | Right 5DP | Yes | Amputation/ Excision | 2 Months |
| Mulvey^A50^ | 1964 | 60/M | Lung Cancer | Squamous cell carcinoma | Right 5DP | Yes | NM | NM |
| Mulvey^A50^ | 1964 | 69/M | Lung Cancer | Squamous cell carcinoma | Left 4PP | Yes | Radiation | "Expired" |
| Grant & Roller^A176^ | 1966 | 63/M | Lung Cancer | Squamous cell carcinoma | Right 5DP | Yes | Amputation/ Excision | 0.5 Months |
| Mohanty et al.^A52^ | 1968 | 70/M | Lung Cancer | Squamous cell carcinoma | Right 2DP | No | NM | 1 Month |
| Camiel et al.^A177^ | 1969 | 47/M | Lung Cancer | Squamous cell carcinoma | Left Palm (ST), Subungal D3 (ST) | No | NM | "Expired" |
| Sneddon^A178^ | 1969 | 53/M | Lung Cancer | Squamous cell carcinoma | Right 4DP | Yes | Amputation/ Excision + Radiation | 2 Months |
| Mladenović et al.^A179^ | 1972 | 44/M | Lung Cancer | Squamous cell carcinoma | Left 1DP, 3DP, 5MP | No | NM | 1 Month |
| Graham et al.^A180^ | 1973 | 47/M | Lung Cancer | Squamous cell carcinoma | Left 3DP | No | Conservative/ Palliative Treatment | "Expired" |
| Singh^A181^ | 1974 | 58/F | Lung Cancer | Squamous cell carcinoma | Right 3DP | No | Amputation/ Excision | NM |
| Seyss^A182^ | 1975 | 68/NM | Lung Cancer | Squamous cell carcinoma | Right 3MP | NM | NM | NM |
| Seyss^A182^ | 1975 | 70/F | Lung Cancer | Squamous cell carcinoma | Right 2DP | NM | NM | NM |
| Lombard & Dubois De Montreynaud^A183^ | 1976 | 49/M | Lung Cancer | Squamous cell carcinoma | Left 5PP | No | Amputation/ Excision | 8 Months |
| La Salle et al.^A184^ | 1977 | 10/M | Lung Cancer | Squamous cell carcinoma | Left 4MC | Yes | Radiation | 6 Months |
| Patel & JW^A185^ | 1978 | 56/M | Lung Cancer | Squamous cell carcinoma | Left 3PP | No | Amputation/ Excision + Radiation | 2 Months |
| Patel & JW^A185^ | 1978 | 69/F | Lung Cancer | Squamous cell carcinoma | Right 5DP | Yes | NM | Alive At Time of Publication |
| Vaezy & Budson^A186^ | 1978 | 52/M | Lung Cancer | Squamous cell carcinoma | Right 4MP | No | Amputation/ Excision | NM |
| Wu et al.^A187^ | 1978 | 70/F | Lung Cancer | Squamous cell carcinoma | Right 1DP | Yes | Chemotherapy/ Immunotherapy | 1 Month |
| Nagendran et al.^A188^ | 1980 | 59/M | Lung Cancer | Squamous cell carcinoma | Right 3DP, 5DP | Yes | Amputation/ Excision | NM |
| Wu et al.^A189^ | 1980 | 70/M | Lung Cancer | Squamous cell carcinoma | Right 4DP | No | Amputation/ Excision | 3 Months |
| Drewes et al.^A190^ | 1981 | 58/M | Lung Cancer | Squamous cell carcinoma | Right 1DP | Yes | Amputation/ Excision | 1 Month |
| Sarma & Socorro^A191^ | 1981 | 52/M | Lung Cancer | Squamous cell carcinoma | Right 4DP | No | Amputation/ Excision | 5 Months |
| Asencio et al.^A113^ | 1982 | 73/M | Lung Cancer | Squamous cell carcinoma | Right 2MC | Yes | Radiation | 11 Months |
| Khokhar & Lee^A192^ | 1983 | 67/M | Lung Cancer | Squamous cell carcinoma | Right 3DP | No | Amputation/ Excision | 6 Months |
| Martin & Dove^A193^ | 1983 | 52/M | Lung Cancer | Squamous cell carcinoma | Right 4DP | No | Radiation | 8 Months |
| Martin & Dove^A193^ | 1983 | 76/M | Lung Cancer | Squamous cell carcinoma | Right 1DP | No | Amputation/ Excision | 4 Months |
| Rose & Wood^A194^ | 1983 | 53/M | Lung Cancer | Squamous cell carcinoma | Left 2DP | No | Amputation/ Excision | NM |
| TaeSooChung^A17^ | 1983 | 65/M | Lung Cancer | Squamous cell carcinoma | Right 5MP, 5DP | Yes | Radiation | 0.5 Months |
| Dubost et al.^A195^ | 1984 | 69/M | Lung Cancer | Squamous cell carcinoma | Right 1MC | Yes | NM | 5 Months |
| Ioia et al.^A196^ | 1984 | 69/M | Lung Cancer | Squamous cell carcinoma | Right Scaphoid | No | Amputation/ Excision | Poor Prognosis |
| Weidmann & Ganz^A197^ | 1984 | 63/M | Lung Cancer | Squamous cell carcinoma | Left 5DP,  Right Palm (ST) | Yes | Amputation/ Excision + Radiation | 2 Months |
| Kosuda et al.^A198^ | 1986 | 47/F | Lung Cancer | Squamous cell carcinoma | Left 3DP | Yes | NM | NM |
| Henderson^A199^ | 1987 | 63/M | Lung Cancer | Squamous cell carcinoma | Left 1DP | No | Amputation/ Excision | NM |
| Sim^A200^ | 1989 | 49/F | Lung Cancer | Squamous cell carcinoma | Right Trapezium, Scaphoid, Lunate, Triquetrum | No | NM | 5 Months |
| Farouk et al.^A201^ | 1990 | 66/M | Lung Cancer | Squamous cell carcinoma | Right 4DP | No | Amputation/ Excision | NM |
| Letanche et al.^A105^ | 1990 | 59/M | Lung Cancer | Squamous cell carcinoma | Right Volar 3MP (ST) | Yes | Amputation/ Excision | 11 Months |
| Stone & Davies^A202^ | 1990 | 51/F | Lung Cancer | Squamous cell carcinoma | Left 5MP, 5DP | Yes | Amputation/ Excision + Radiation | "Expired" |
| Desmanet et al.^A203^ | 1991 | 62/M | Lung Cancer | Squamous cell carcinoma | Right Hamate | Yes | Radiation | 10 Months |
| Moens et al.^A204^ | 1993 | 65/M | Lung Cancer | Squamous cell carcinoma | Right 5DP | Yes | Amputation/ Excision | 5 Months |
| Roncaglio et al.^A58^ | 1993 | 55/M | Lung Cancer | Squamous cell carcinoma | Right 4DP | Yes | Amputation/ Excision | 1 Month |
| Abrahams^A60^ | 1995 | 59/F | Lung Cancer | Squamous cell carcinoma | Left Lunate | No | NM | NM |
| De Maeseneer et al.^A205^ | 1995 | 60/M | Lung Cancer | Squamous cell carcinoma | Left 1DP,  Right 1DP | Yes | Radiation | NM |
| Castello et al.^A5^ | 1996 | 74/M | Lung Cancer | Squamous cell carcinoma | Right 1DP | Yes | Amputation/ Excision | 3 Months |
| Saglike et al.^A206^ | 1996 | 36/M | Lung Cancer | Squamous cell carcinoma | Left 2DP, 4DP | No | Amputation/ Excision + Radiation | 12 Months |
| Hatakeyama et al.^A207^ | 1997 | 72/M | Lung Cancer | Squamous cell carcinoma | Right 1MC | No | Radiation | Poor Prognosis |
| Baran et al.^A208^ | 1998 | 70/F | Lung Cancer | Squamous cell carcinoma | Left 3DP | Yes | NM | "Expired" |
| Chang et al.^A209^ | 1999 | 57/M | Lung Cancer | Squamous cell carcinoma | Right Subungal D1 (ST) | No | Radiation | "Expired" |
| Vanhooteghem et al.^A210^ | 1999 | 61/F | Lung Cancer | Squamous cell carcinoma | Left Subungal D5 (ST),  Right Subungal D5 (ST) | No | Radiation | 5 Months |
| Ryu et al.^A211^ | 2000 | 63/M | Lung Cancer | Squamous cell carcinoma | NS Subungal D4 (ST) | No | Radiation | 6 Months |
| Caglar & Ceylan^A212^ | 2001 | 51/M | Lung Cancer | Squamous cell carcinoma | Right Capitate, Hamate | No | NM | NM |
| Theunissen et al.^A213^ | 2002 | 63/M | Lung Cancer | Squamous cell carcinoma | Right Pulp D1 (ST), Pulp D3 (ST) | No | Conservative/ Palliative Treatment | NM |
| Sahbaz et al.^A214^ | 2004 | 54/M | Lung Cancer | Squamous cell carcinoma | Right 4DP | Yes | Radiation | NM |
| Nakamura et al.^A215^ | 2005 | 71/M | Lung Cancer | Squamous cell carcinoma | Left Pulp D4 (ST) | Yes | NM | NM |
| Nakamura et al.^A215^ | 2005 | 73/M | Lung Cancer | Squamous cell carcinoma | Left Pulp D1 (ST) | No | Radiation | 6 Months |
| Gawley et al.^A216^ | 2006 | 65/M | Lung Cancer | Squamous cell carcinoma | Left 5DP | No | Amputation/ Excision | 3 Months |
| Elhassan & Fakhouri^A217^ | 2007 | 68/M | Lung Cancer | Squamous cell carcinoma | Left 1MC | No | Amputation/ Excision | Alive At Time of Publication |
| Han et al.^A69^ | 2007 | 51/M | Lung Cancer | Squamous cell carcinoma | NS 5DP | No | Amputation/ Excision | 22 Months |
| Bahar et al.^A218^ | 2008 | 68/F | Lung Cancer | Squamous cell carcinoma | Right Pulp D3 (ST) | Yes | Amputation/ Excision | 36 Months |
| Madjidi et al.^A219^ | 2009 | 55/M | Lung Cancer | Squamous cell carcinoma | Right 2DP | No | Amputation/ Excision | 2 Months |
| Lozíc et al.^A220^ | 2010 | 68/M | Lung Cancer | Squamous cell carcinoma | Right 1DP | No | Amputation/ Excision | 3.5 Months |
| Lucilli et al.^A221^ | 2010 | 63/M | Lung Cancer | Squamous cell carcinoma | Left 1DP | Yes | Amputation/ Excision | 6 Months |
| Chao et al.^A222^ | 2011 | 62/F | Lung Cancer | Squamous cell carcinoma | Left 3PP | Yes | Radiation | NM |
| Ingerslev^A223^ | 2014 | 80/M | Lung Cancer | Squamous cell carcinoma | Right 4DP | No | Amputation/ Excision | 6 Months |
| Lambe et al.^A224^ | 2014 | 72/M | Lung Cancer | Squamous cell carcinoma | Right 5DP | Yes | Radiation | 0.5 Months |
| Walton et al.^A225^ | 2014 | 56/M | Lung Cancer | Squamous cell carcinoma | Right Palm (ST) | Yes | NM | NM |
| Babacan et al.^A226^ | 2015 | 55/M | Lung Cancer | Squamous cell carcinoma | NS "Subungal (ST)" | No | Radiation | 10 Months |
| Baltazard et al.^A227^ | 2015 | 49/M | Lung Cancer | Squamous cell carcinoma | Right Subungal D3 (ST) | No | Chemotherapy/ Immunotherapy | 1 Month |
| Soylemez et al.^A228^ | 2015 | 47/M | Lung Cancer | Squamous cell carcinoma | Left 5DP,  Right 2DP | Yes | Amputation/ Excision | Alive At Time of Publication |
| Sahoo et al.^A229^ | 2016 | 79/M | Lung Cancer | Squamous cell carcinoma | Left 2DP | No | Amputation/ Excision | NM |
| Feng et al.^A93^ | 2017 | 53/M | Lung Cancer | Squamous cell carcinoma | Right 1PP | Yes | Radiation | 3 Months |
| Muñoz-Mahamud et al.^A230^ | 2017 | 52/F | Lung Cancer | Squamous cell carcinoma | Left 3MP | No | Amputation/ Excision | 12 Months |
| Muñoz-Mahamud et al.^A230^ | 2017 | 63/M | Lung Cancer | Squamous cell carcinoma | Right 1MC | No | Declined Treatment | 6 Months |
| Ross & Mann^A231^ | 2017 | 78/M | Lung Cancer | Squamous cell carcinoma | Left 3DP,  Right 5DP | Yes | Amputation/ Excision | "Expired" |
| Espinosa et al.^A232^ | 2018 | 61/M | Lung Cancer | Squamous cell carcinoma | Left 2MC | No | NM | NM |
| Castillo & Albayda^A233^ | 2019 | 60/M | Lung Cancer | Squamous cell carcinoma | Left 3DP, Pulp D1 (ST) | Yes | Declined Treatment | 0.75 Months |
| Khaja et al.^A234^ | 2019 | 78/F | Lung Cancer | Squamous cell carcinoma | Right Palm (ST) | No | Conservative/ Palliative Treatment | 1 Month |
| Peeters & Gosens^A235^ | 2019 | 79/M | Lung Cancer | Squamous cell carcinoma | Right 4MP | No | Radiation | 8 Months |
| Garabet Diramerian et al.^A236^ | 2020 | 69/M | Lung Cancer | Squamous cell carcinoma | Right 2DP | No | NM | Poor Prognosis |
| Hirano et al.^A237^ | 2020 | 70/M | Lung Cancer | Squamous cell carcinoma | Right 4DP | No | NM | NM |
| Cottignoli et al.^A238^ | 2021 | 53/M | Lung Cancer | Squamous cell carcinoma | Left 1DP | No | Amputation/ Excision | NM |
| Dow et al. | 2021 | 84/M | Lung Cancer | Squamous cell carcinoma | Left 5PP | Yes | Amputation/ Excision + Radiation | Alive At Time of Publication |
| Nivar et al.^A239^ | 2021 | 56/F | Lung Cancer | Squamous cell carcinoma | Right Trapezium | No | Radiation | 9 Months |
| Hicks et al.^A49^ | 1964 | 53/M | Lung Cancer | Undifferentiated Carcinoma | Left 3PP | Yes | Radiation | 7 Months |
| Mulvey^A50^ | 1964 | 32/F | Lung Cancer | Undifferentiated Carcinoma | Left 3DP | Yes | Radiation | 4 Months |
| **Breast Cancer** | | | | | | | | |
| Kerin^A168^ | 1958 | 62/F | Breast Cancer | Adenocarcinoma | Right Trapezium, Trapezoid, Capitate, Scaphoid, 1MC, 2MC, 3MC | Yes | Conservative/ Palliative Treatment | 2 Months |
| Pfeiffer^A170^ | 1959 | 42/F | Breast Cancer | Adenocarcinoma | Left 4MC,  Right 1DP, 2DP | Yes | NM | 2.5 Months |
| Mangini^A240^ | 1967 | 56/F | Breast Cancer | Adenocarcinoma | Right 1MC | Yes | Amputation/ Excision | NM |
| Michel et al.^A241^ | 1967 | 78/F | Breast Cancer | Adenocarcinoma | Right 4MC, 5MC, Dorsum of Hand (ST) | Yes | Amputation/ Excision | 39 Months |
| Panebianco & Kaupp^A242^ | 1968 | 74/F | Breast Cancer | Adenocarcinoma | Left 1DP,  Right 1DP | Yes | Amputation/ Excision | 7 Months |
| Nadzhmitdinov & Zadarski^A243^ | 1970 | 46/F | Breast Cancer | Adenocarcinoma | Right 2DP | Yes | Amputation/ Excision | NM |
| Kumar & Kovi^A244^ | 1978 | 53/F | Breast Cancer | Adenocarcinoma | Right 2MC | Yes | Radiation | 3 Months |
| Liszka et al.^A245^ | 1980 | 42/F | Breast Cancer | Adenocarcinoma | Right Scaphoid | Yes | NM | NM |
| Vijayakumar & Creditor^A246^ | 1986 | 91/F | Breast Cancer | Adenocarcinoma | Right 1DP | Yes | Radiation | 3 Months |
| Vadivelu & Drew^A247^ | 2002 | 46/F | Breast Cancer | Adenocarcinoma | Right 4MP | No | Radiation + Chemotherapy/ Immunotherapy | Alive At Time of Publication |
| French et al.^A248^ | 2007 | 39/F | Breast Cancer | Adenocarcinoma | Right Palm (ST), Dorsum of Hand (ST) | Yes | Amputation/ Excision | 18 Months |
| Wavreille et al.^A249^ | 2009 | 48/F | Breast Cancer | Adenocarcinoma | Right 5DP | Yes | Amputation/ Excision | NM |
| Biyi et al.^A250^ | 2010 | 37/F | Breast Cancer | Adenocarcinoma | Left 1PP | Yes | Radiation | 18 Months |
| Brygger & Cold^A251^ | 2015 | 41/F | Breast Cancer | Adenosquamous | Left 2DP, Subungal D2 (ST) | Yes | NM | 4 Months |
| Basora & Fery^A252^ | 1975 | 63/F | Breast Cancer | Cystosarcoma phylloides | Right 4DP | Yes | Amputation/ Excision | 0.25 Months |
| Patel et al.^A253^ | 1985 | 47/F | Breast Cancer | Cystosarcoma phylloides | Left 5DP | Yes | Amputation/ Excision | 1.5 Months |
| Looi & Arumugam^A254^ | 2021 | 58/F | Breast Cancer | Cystosarcoma phylloides | Left 3DP | Yes | Amputation/ Excision | 0.5 Months |
| Umebayashi^A255^ | 2004 | 68/M | Breast Cancer | Extramammary Paget's Disease | Right 4DP | Yes | Conservative/ Palliative Treatment | 0.75 Months |
| Cary et al.^A129^ | 1981 | 32/F | Breast Cancer | Infiltrating Ductal Carcinoma | Right Trapezium | Yes | Amputation/ Excision | NM |
| Morris & House^A131^ | 1985 | 42/F | Breast Cancer | Infiltrating Ductal Carcinoma | Right 3DP | Yes | Amputation/ Excision | 2 Months |
| Wu et al.^A256^ | 2009 | 74/F | Breast Cancer | Infiltrating Ductal Carcinoma | Right Volar 3MP (ST) | No | NM | Alive At Time of Publication |
| Ravind et al.^A257^ | 2015 | 40/F | Breast Cancer | Infiltrating Ductal Carcinoma | Left 2PP | Yes | Radiation | Poor Prognosis |
| Toth^A258^ | 1983 | 30/F | Breast Cancer | Medullary Carcinoma | Left Pulp D5 (ST),  Right Pulp D1 (ST) | Yes | Radiation | 10 Months |
| Ammons et al.^A259^ | 2021 | 70/F | Breast cancer | Metaplastic carcinoma | Left 1DP | Yes | Radiation | Alive At Time of Publication |
| Carty et al.^A260^ | 2006 | 68/F | Breast Cancer | Mixed Ductal Lobular Carcinoma | Left 3PP | Yes | Amputation/ Excision | Alive At Time of Publication |
| Vijaya et al.^A261^ | 2011 | 38/F | Breast Cancer | Mucinous Carcinoma | Left Pulp D1 (ST), Pulp D2 (ST), Pulp D3 (ST), Pulp D4 (ST), Pulp D5 (ST) | Yes | NM | NM |
| Baños-Arévalo et al.^A147^ | 2018 | 76/F | Breast Cancer | Myoepithelial carcinoma with sqamous differentiation | Right Pulp D3 (ST) | Yes | Conservative/ Palliative Treatment | "Expired" |
| De Smet^A262^ | 2004 | 71/F | Breast Cancer | Neuroendocrine | Right 2MC | Yes | Radiation | Alive At Time of Publication |
| Handley^A263^ | 1906 | NM/F | Breast Cancer | NM | Right 3MC, 4MC, 5MC | Yes | NM | NM |
| Bendick & Jacobs^A264^ | 1925 | 39/F | Breast Cancer | NM | Left Lunate, 1MC, 2MC, 2PP, 3DP, 4PP,  Right 3MC, 5MC, 3PP, 4PP, 4MP, 5PP | Yes | Conservative/ Palliative Treatment | 4 Months |
| Pack^A265^ | 1939 | NM/F | Breast Cancer | NM | NS "Phalanx" | NM | NM | NM |
| Grilli^A266^ | 1958 | 52/F | Breast Cancer | NM | Left 5MC | NM | NM | NM |
| Toubiana & Proux^A267^ | 1965 | 50/F | Breast Cancer | NM | Left 1DP, 2DP, 3DP | Yes | Conservative/ Palliative Treatment | 1 Month |
| Toubiana & Proux^A267^ | 1965 | 57/F | Breast Cancer | NM | Left Scaphoid, Lunate, Triquetrum,  Right 3MP | Yes | Radiation | 11 Months |
| Carroll^A268^ | 1975 | NM/M | Breast Cancer | NM | Left 2MC | NM | NM | NM |
| Wu & Guise^A187^ | 1978 | 42/F | Breast Cancer | NM | Right 4MP | Yes | NM | 4 Months |
| Wu & Guise^A187^ | 1978 | 46/F | Breast Cancer | NM | Left Subungal D4 (ST) | Yes | Conservative/ Palliative Treatment | 3 Months |
| Liszka et al.^A245^ | 1980 | 49/F | Breast Cancer | NM | Left Capitate, 3MC, 4MC | Yes | NM | NM |
| Morris & House^A131^ | 1985 | 62/F | Breast Cancer | NM | Right 1DP | NM | NM | 4 Months |
| Bloom et al.^A269^ | 1992 | 51/F | Breast Cancer | NM | Left "Diffuse Bones",  Right "Diffuse Bones" | Yes | Radiation | Alive At Time of Publication |
| Witthaut & Steffens^A270^ | 1996 | 67/F | Breast Cancer | NM | Left 4MP | Yes | Amputation/ Excision | Alive At Time of Publication |
| Kaplan et al.^A271^ | 2000 | 71/F | Breast Cancer | NM | Right Capitate, Scaphoid, 3MC 3PP, 3MP, 3DP | Yes | Radiation + Chemotherapy/ Immunotherapy | NM |
| Asthana et al.^A272^ | 2001 | 40/F | Breast Cancer | NM | Left 1PP | Yes | Radiation | 6 Months |
| Flynn et al.^A142^ | 2008 | 65/F | Breast Cancer | NM | Right Capitate, 3MC, 5MC | Yes | Radiation | Alive At Time of Publication |
| Sur et al.^A18^ | 2011 | 63/F | Breast Cancer | NM | Right Pulp D2 (ST) | Yes | Amputation/ Excision | 8 Months |
| Cattelan & Dumontier^A273^ | 2021 | 68/F | Breast Cancer | NM | Left 5DP | Yes | Conservative/ Palliative Treatment | 6 Months |
| Hicks et al.^A49^ | 1964 | 73/F | Breast Cancer | Scirrhous carcinoma | Left 3MP | Yes | Declined Treatment | NM |
| **Liver Cancers** | | | | | | | | |
| Reichbach et al.^A274^ | 1970 | 62/M | Liver Cancer | Hepatocellular carcinoma | Right 1DP | No | Amputation/ Excision | 7 Months |
| Delsmann et al.^A275^ | 1998 | 62/M | Liver Cancer | Hepatocellular carcinoma | Right 1MC | No | Amputation/ Excision | 15 Months |
| Lee et al.^A276^ | 1999 | 47/M | Liver Cancer | Hepatocellular carcinoma | Right 1DP | Yes | Amputation/ Excision | 5 Months |
| Fang et al.^A277^ | 2001 | 49/M | Liver Cancer | Hepatocellular carcinoma | Right Palm (ST), Dorsum of Hand (ST), Pulp D2 (ST) | Yes | Amputation/ Excision | 2 Months |
| Fontana et al.^A278^ | 2004 | 62/M | Liver Cancer | Hepatocellular carcinoma | Right 2MP | Yes | Conservative/ Palliative Treatment | 4 Months |
| Corrales Pinzón et al.^A279^ | 2014 | 59/M | Liver Cancer | Hepatocellular carcinoma | Right Trapezium, Trapezoid, Capitate, Hamate, Scaphoid, Lunate, Triquetrum, Pisiform | No | Conservative/ Palliative Treatment | Poor Prognosis |
| Rauer et al.^A280^ | 2016 | 70/M | Liver Cancer | Hepatocellular carcinoma | Left 4DP | No | Amputation/ Excision | NM |
| Otsuji et al.^A281^ | 2009 | 49/F | Liver Cancer | NM | Left 5DP | Yes | Amputation/ Excision | Alive At Time of Publication |
| Kim et al.^A282^ | 2012 | 55/M | Liver Cancer | NM | Left Pulp D1 (ST) | Yes | Amputation/ Excision | 6 Months |
| Rauf et al.^A283^ | 2012 | 66/M | Liver Cancer | NM | Left 5DP | No | Amputation/ Excision | 2 Months |
| Rommer et al.^A284^ | 2014 | 30/M | Liver Cancer | NM | Left 4DP, 5DP | Yes | Amputation/ Excision | NM |
| Boldo et al.^A285^ | 2020 | 66/F | Liver Cancer | NM | Left Subungal D1 (ST) | Yes | Conservative/ Palliative Treatment | 0.5 Months |
| **Cancers of the Digestive Tract** | | | | | | | | |
| Knapp & Abdul-Karim^A59^ | 1994 | 38/M | Esophageal Cancer | Adenocarcinoma | Left 4MP | Yes | Radiation | 3 Months |
| Mousavi et al.^A286^ | 2005 | 80/F | Esophageal Cancer | Adenocarcinoma | Right 1DP | Yes | Amputation/ Excision | NM |
| Wurapa et al.^A287^ | 2010 | 80/F | Esophageal Cancer | Adenocarcinoma | Left Trapezium, Trapezoid, Scaphoid | Yes | Amputation/ Excision | 7 Months |
| Jenzer et al.^A288^ | 2011 | 85/M | Esophageal Cancer | Adenocarcinoma | Right 4PP | Yes | Amputation/ Excision | 2 Months |
| Kamolz et al.^A289^ | 2012 | 69/M | Esophageal Cancer | Adenocarcinoma | Left Palm (ST) | Yes | Amputation/ Excision | NM |
| Zhang et al.^A290^ | 2020 | 57/M | Esophageal Cancer | Adenocarcinoma | Left Scaphoid | No | Amputation/ Excision | 6 Months |
| Katsumata et al.^A291^ | 2021 | 74/M | Esophageal Cancer | Basaloid squamous cell carcinoma | Right 5DP | Yes | Amputation/ Excision | Alive At Time of Publication |
| Kanatani et al.^A292^ | 2008 | 57/M | Esophageal Cancer | NM | Left 5DP | Yes | Amputation/ Excision | 2 Months |
| Zakharov^A293^ | 1970 | 61/F | Esophageal Cancer | Squamous cell carcinoma | Left 1DP | Yes | Amputation/ Excision | Alive At Time of Publication |
| Kumar^A294^ | 1975 | 64/M | Esophageal Cancer | Squamous cell carcinoma | Right 2MC, 3MC, 4MC, 5MC | Yes | Radiation | 4 Months |
| Levack et al.^A295^ | 1983 | 38/F | Esophageal Cancer | Squamous cell carcinoma | Left 3DP | No | Amputation/ Excision | NM |
| Kosuda et al.^A198^ | 1986 | 61/M | Esophageal Cancer | Squamous cell carcinoma | Left 4DP | Yes | NM | NM |
| Haas et al.^A296^ | 1988 | 51/F | Esophageal Cancer | Squamous cell carcinoma | Right 5PP | Yes | Radiation | 6 Months |
| Tenenbaum et al.^A297^ | 1988 | 56/M | Esophageal Cancer | Squamous cell carcinoma | Right Capitate, Hamate, Wrist (ST) | No | Radiation | Alive At Time of Publication |
| Desmanet et al.^A203^ | 1991 | 48/M | Esophageal Cancer | Squamous cell carcinoma | Right 4DP | Yes | Amputation/ Excision | 8 Months |
| Moutet et al.^A298^ | 1991 | 56/M | Esophageal Cancer | Squamous cell carcinoma | Right Capitate | Yes | Amputation/ Excision | 24 Months |
| Umebayashi^A299^ | 1998 | 81/M | Esophageal Cancer | Squamous cell carcinoma | Right 2DP | Yes | Conservative/ Palliative Treatment | 1.5 Months |
| Yasaka et al.^A300^ | 1999 | 64/M | Esophageal Cancer | Squamous cell carcinoma | Left Pulp D5 (ST) | Yes | NM | 2 Months |
| Houston & Telepak^A301^ | 2000 | 56/M | Esophageal Cancer | Squamous cell carcinoma | Left 4DP | Yes | Amputation/ Excision | NM |
| Silfen et al.^A302^ | 2001 | 75/M | Esophageal Cancer | Squamous cell carcinoma | Right Pulp D4 (ST) | No | Chemotherapy/ Immunotherapy | 5 Months |
| Bujanda^A303^ | 2003 | 56/M | Esophageal Cancer | Squamous cell carcinoma | Left 1PP | Yes | Radiation | NM |
| Dimri et al.^A304^ | 2003 | 60/M | Esophageal Cancer | Squamous cell carcinoma | Right 5DP | Yes | Conservative/ Palliative Treatment | 1 Month |
| Chou et al.^A305^ | 2004 | 63/M | Esophageal Cancer | Squamous cell carcinoma | Left Pulp D4 (ST),  Right Pulp D1 (ST), Pulp D4 (ST) | Yes | Conservative/ Palliative Treatment | 0.5 Months |
| Hsieh et al.^A306^ | 2008 | 56/M | Esophageal Cancer | Squamous cell carcinoma | Left 1PP | Yes | NM | NM |
| Dar et al.^A307^ | 2011 | 75/M | Esophageal Cancer | Squamous cell carcinoma | Left 1DP, 2DP, 3DP, 4DP, 5DP,  Right Pulp 1DP (ST), Pulp 2DP (ST), Pulp 3DP (ST), Pulp 4DP (ST), Pulp 5DP (ST) | Yes | Amputation/ Excision | Alive At Time of Publication |
| Kumar et al.^A30^ | 2011 | 52/M | Esophageal Cancer | Squamous cell carcinoma | Left 5DP | No | Radiation | NM |
| Purkayastha^A308^ | 2015 | 65/F | Esophageal Cancer | Squamous cell carcinoma | Right 4DP | Yes | Amputation/ Excision | Alive At Time of Publication |
| Purkayastha^A308^ | 2015 | 70/F | Esophageal Cancer | Squamous cell carcinoma | Left 2DP | Yes | Amputation/ Excision | Alive At Time of Publication |
| Arbeláez Echeverri et al.^A309^ | 2019 | 57/M | Esophageal Cancer | Squamous cell carcinoma | Left 1DP | Yes | NM | NM |
| Chaudhary et al.^A310^ | 2020 | 68/M | Esophageal Cancer | Squamous cell carcinoma | Left 2DP | Yes | Amputation/ Excision | NM |
| Mangini^A240^ | 1967 | 58/M | Gastric Cancer | Adenocarcinoma | Left 5MC | No | Amputation/ Excision | NM |
| Craigen & Chesney^A311^ | 1988 | 37/M | Gastric Cancer | Adenocarcinoma | Right Hamate | Yes | NM | NM |
| DiSpaltro et al.^A312^ | 1992 | 41/M | Gastric Cancer | Adenocarcinoma | Right Pulp D5 (ST) | Yes | Amputation/ Excision | NM |
| Okada et al.^A313^ | 1999 | 84/F | Gastric Cancer | Adenocarcinoma | Left Pulp D4 (ST) | Yes | Conservative/ Palliative Treatment | 12 Months |
| Chang et al.^A314^ | 2001 | 66/M | Gastric Cancer | Adenocarcinoma | Right 4MC | No | Radiation | 3 Months |
| Bahk et al.^A315^ | 2006 | 67/F | Gastric Cancer | Adenocarcinoma | Left 1DP, 2DP, 3DP, 4DP, 5DP | Yes | Amputation/ Excision | 4 Months |
| Park et al.^A316^ | 2006 | 39/F | Gastric Cancer | Adenocarcinoma | Left Capitate,  Right Trapezium | Yes | Radiation | Alive At Time of Publication |
| Bigot et al.^A317^ | 2007 | 64/M | Gastric Cancer | Adenocarcinoma | Right 3MC | Yes | Radiation | 5 Months |
| Miyamoto et al.^A318^ | 2008 | 72/F | Gastric Cancer | Adenocarcinoma | Left 5MC | Yes | Amputation/ Excision | 12 Months |
| Spiteri et al.^A319^ | 2008 | 82/M | Gastric Cancer | Adenocarcinoma | Right 4DP | No | Amputation/ Excision | Alive At Time of Publication |
| Sur et al.^A18^ | 2011 | 67/F | Gastric Cancer | Adenocarcinoma | Left 1DP, 2DP, 3DP, 4DP, 5DP, Pulp D1 (ST), Pulp D2 (ST), Pulp D3 (ST), Pulp D4 (ST), Pulp D5 (ST) | Yes | Amputation/ Excision | 4 Months |
| Naito et al.^A320^ | 2015 | 55/M | Gastric Cancer | Adenocarcinoma | Right Scaphoid | Yes | Amputation/ Excision | 3 Months |
| Okamoto et al.^A321^ | 2017 | 62/M | Gastric Cancer | Adenocarcinoma | Right Trapezium | No | Amputation/ Excision | 36 Months |
| Gomi et al.^A322^ | 2018 | 62/M | Gastric Cancer | Adenocarcinoma | Right Trapezium | No | Radiation | Alive At Time of Publication |
| Kumar^A323^ | 2019 | 58/M | Gastric Cancer | Adenocarcinoma | Left Trapezium, Trapezoid, 2MC | Yes | Radiation | Alive At Time of Publication |
| Harris et al.^A324^ | 2020 | 75/F | Gastric Cancer | Adenocarcinoma | Right 1DP | Yes | Radiation | Poor Prognosis |
| Kerin^A168^ | 1958 | 61/M | Colon Cancer | Adenocarcinoma | Left 3DP | Yes | Amputation/ Excision | 7 Months |
| Drury^A325^ | 1959 | 75/M | Colon Cancer | Adenocarcinoma | Right Subungal D4 (ST) | Yes | Amputation/ Excision | Alive At Time of Publication |
| Hummel & Scott^A326^ | 1962 | 52/F | Colon Cancer | Adenocarcinoma | Left 3PP | Yes | Radiation | 5 Months |
| Guttmann & Stein^A327^ | 1968 | 84/F | Colon Cancer | Adenocarcinoma | Right 1PP | Yes | Amputation/ Excision | NM |
| Gottlieb & Schermer^A328^ | 1970 | 72/F | Colon Cancer | Adenocarcinoma | Left Thenar Eminence (ST), Pulp D1 (ST), Pulp D2 (ST), Pulp D3 (ST),  Right Pulp D1 (ST) | Yes | NM | 6.5 Months |
| Bryan et al.^A329^ | 1974 | 61/M | Colon Cancer | Adenocarcinoma | Left Lunate | NM | Radiation | Alive At Time of Publication |
| Buckley & Peebles Brown^A330^ | 1987 | 61/F | Colon Cancer | Adenocarcinoma | Left 4PP | Yes | Amputation/ Excision + Radiation | 2 Months |
| Buckley & Peebles Brown^A330^ | 1987 | 78/F | Colon Cancer | Adenocarcinoma | Left Trapezium, Trapezoid | Yes | Chemotherapy/ Immunotherapy | Alive At Time of Publication |
| Hindley & Metcalfe^A331^ | 1987 | 44/M | Colon Cancer | Adenocarcinoma | Right 5MP | Yes | Amputation/ Excision | NM |
| Bourne et al.^A332^ | 1988 | 74/F | Colon Cancer | Adenocarcinoma | Right 2MC, 3MC, 4MC | Yes | Radiation | 2 Months |
| Müller et al.^A333^ | 1988 | 77/M | Colon Cancer | Adenocarcinoma | Left Trapezium, Trapezoid, Capitate, Hamate, Scaphoid, Lunate, Triquetrum, Pisiform, Dorsum of Hand (ST) | Yes | Radiation + Chemotherapy/ Immunotherapy | NM |
| Mendez Lopez et al.^A334^ | 1997 | 54/M | Colon Cancer | Adenocarcinoma | Right 1MC | Yes | Conservative/ Palliative Treatment | 1 Month |
| Rümenapf et al.^A335^ | 1997 | 69/M | Colon Cancer | Adenocarcinoma | Left 2DP | Yes | Amputation/ Excision | 24 Months |
| Baron et al.^A336^ | 1998 | 59/F | Colon Cancer | Adenocarcinoma | Left 2DP | Yes | Radiation | NM |
| Augustine et al.^A337^ | 2000 | 50/M | Colon Cancer | Adenocarcinoma | Left 3PP, 3MP, 3DP | No | Amputation/ Excision | Lost to Follow-up |
| Filloux & Fontaine^A21^ | 2000 | 62/M | Colon Cancer | Adenocarcinoma | Left Pulp D2 (ST) | Yes | Amputation/ Excision | 9 Months |
| Henderson & Jehangir^A338^ | 2001 | 52/M | Colon Cancer | Adenocarcinoma | Right 4DP | No | Amputation/ Excision | 11 Months |
| Oron et al.^A339^ | 2003 | 77/F | Colon Cancer | Adenocarcinoma | Left 2PP | Yes | Amputation/ Excision + Radiation | NM |
| Mátrai et al.^A340^ | 2005 | 63/F | Colon Cancer | Adenocarcinoma | Right 4MC | Yes | Amputation/ Excision | NM |
| Ozcanli et al.^A341^ | 2005 | 42/F | Colon Cancer | Adenocarcinoma | Right 3MC | Yes | Amputation/ Excision | NM |
| Gallagher et al.^A342^ | 2006 | 72/M | Colon Cancer | Adenocarcinoma | Left Subungal D1 (ST) | Yes | Amputation/ Excision | 6 Months |
| Gamblin et al.^A343^ | 2006 | 72/M | Colon Cancer | Adenocarcinoma | Right 2DP | Yes | Amputation/ Excision | Alive At Time of Publication |
| Ishikawa et al.^A344^ | 2007 | 76/M | Colon Cancer | Adenocarcinoma | Left Palm (ST) | Yes | Amputation/ Excision | 4 Months |
| Nikolić et al.^A345^ | 2007 | 67/M | Colon Cancer | Adenocarcinoma | Right 1PP | Yes | Amputation/ Excision | 39 Months |
| Anoop et al.^A346^ | 2010 | 76/M | Colon Cancer | Adenocarcinoma | Right 5DP | No | Radiation | 1 Month |
| Borobio et al.^A347^ | 2010 | 81/F | Colon Cancer | Adenocarcinoma | Left Trapezium, Trapezoid, Hamate, 4MC | Yes | Radiation | 7 Months |
| Vasić^A348^ | 2010 | 52/NM | Colon Cancer | Adenocarcinoma | Right 1PP, 1DP | Yes | Radiation | NM |
| Çetın et al.^A349^ | 2011 | 53/M | Colon Cancer | Adenocarcinoma | Right Thenar Eminence (ST) | Yes | NM | NM |
| Roohi et al.^A350^ | 2011 | 57/F | Colon Cancer | Adenocarcinoma | Right Trapezoid, Capitate, Hamate, Lunate, Triquetrum, Pisiform, 1MC, 3MC, 4MC, 5MC, 2PP, 2MP, 2DP, 3PP, 3MP, 3DP, 4PP, 4MP, 4DP, 5PP, 5MP, 5DP | Yes | Amputation/ Excision | Alive At Time of Publication |
| Fadli et al.^A351^ | 2012 | 67/F | Colon Cancer | Adenocarcinoma | Left 1MC | Yes | Conservative/ Palliative Treatment | Poor Prognosis |
| Gharwan et al.^A352^ | 2012 | 54/M | Colon Cancer | Adenocarcinoma | Right 3PP, 3DP, 3MC | No | Amputation/ Excision | 23 Months |
| Ko et al.^A353^ | 2014 | 80/M | Colon Cancer | Adenocarcinoma | Right Pulp D1 (ST) | Yes | Radiation | 2 Months |
| Wang et al.^A354^ | 2016 | 57/F | Colon Cancer | Adenocarcinoma | Left 3PP | Yes | Radiation | Alive At Time of Publication |
| Ito et al.^A355^ | 2017 | 54/F | Colon Cancer | Adenocarcinoma | Right 1MC | Yes | Chemotherapy/ Immunotherapy | Alive At Time of Publication |
| Rau^A356^ | 2019 | 79/M | Colon Cancer | Adenocarcinoma | Left Thenar eminence (ST) | Yes | Referred to Oncology | Alive At Time of Publication |
| Voskuil et al.^A357^ | 2019 | 81/M | Colon Cancer | Adenocarcinoma | Right Scaphoid | Yes | Amputation/ Excision | 12 Months |
| Gordinou de Gouberville et al.^A358^ | 2021 | 71/M | Colon Cancer | Adenocarcinoma | NS 5DP | Yes | Amputation/ Excision | NM |
| Verardino et al.^A359^ | 2011 | 59/F | Colon Cancer | Basaloid Carcinoma | Left 4DP,  Right 4DP | Yes | Radiation | NM |
| Brette et al.^A360^ | 1961 | 49/F | Colon Cancer | Epithelioma | Left 3MC | Yes | Conservative/ Palliative Treatment | 2 Months |
| Lichtenstein^A361^ | 1952 | NM/NM | Colon Cancer | NM | Left "Hand",  Right "Hand" | No | NM | NM |
| Wu & Guise^A187^ | 1978 | 83/M | Colon Cancer | NM | Right Dorsum of Hand (ST) | Yes | Conservative/ Palliative Treatment | 6 Months |
| Bricout^A128^ | 1981 | 76/M | Colon Cancer | NM | Right 1PP | Yes | Radiation | NM |
| Sur et al.^A18^ | 2011 | 74/F | Colon Cancer | NM | Left "PP" | NM | Amputation/ Excision | 3 Months |
| Molina-Cerrillo^A362^ | 2019 | 81/F | Colon Cancer | NM | Right 4MP | Yes | Radiation | NM |
| Cattelan & Dumontier^A273^ | 2021 | 65/F | Colon Cancer | NM | Left 1DP | Yes | Radiation | 12 Months |
| Henkert & Berge^A363^ | 1991 | 53/M | Colon Cancer | Papillary gland carcinoma | Left 5DP | Yes | NM | 12 Months |
| Balta et al.^A364^ | 2011 | 38/M | Colon Cancer | Undifferentiated carcinoma | Left Palm (ST) | Yes | Amputation/ Excision | NM |
| **Cancers of the Urinary System** | | | | | | | | |
| Fabre & Dambrin^A365^ | 1935 | 66/F | Kidney Cancer | Clear Cell Carcinoma | Right 2MC | No | Conservative/ Palliative Treatment | 5 Months |
| Kinsella^A366^ | 1957 | 52/F | Kidney Cancer | Clear Cell Carcinoma | Left 4DP | Yes | Amputation/ Excision | 1 Month |
| Schmitt-Köppler & Richter^A367^ | 1969 | 56/M | Kidney Cancer | Clear Cell Carcinoma | Left 2DP | Yes | Amputation/ Excision | Alive At Time of Publication |
| De Oliveira et al.^A368^ | 1978 | 48/F | Kidney Cancer | Clear Cell Carcinoma | Left 4DP | Yes | Amputation/ Excision | 2 Months |
| Kobus et al.^A369^ | 1992 | 65/F | Kidney Cancer | Clear Cell Carcinoma | Left 3MC | Yes | Amputation/ Excision + Radiation | Alive At Time of Publication |
| Vine & Cohen^A370^ | 1996 | 57/F | Kidney Cancer | Clear Cell Carcinoma | Left Subungal D1 (ST) | Yes | Radiation | 3 Months |
| Adegboyega et al.^A371^ | 1999 | 60/M | Kidney Cancer | Clear Cell Carcinoma | Right 3MP | No | Amputation/ Excision | 11 Months |
| Ghert et al.^A372^ | 2001 | 56/F | Kidney Cancer | Clear Cell Carcinoma | Left 2MP | Yes | Radiation | Alive At Time of Publication |
| Tolo et al.^A373^ | 2002 | 63/M | Kidney Cancer | Clear Cell Carcinoma | Left Trapezium | Yes | Amputation/ Excision | 13 Months |
| Mitrovic et al.^A374^ | 2009 | 67/M | Kidney Cancer | Clear Cell Carcinoma | Left 4PP | No | Amputation/ Excision | NM |
| Anglada-Curado et al.^A375^ | 2010 | 75/F | Kidney Cancer | Clear Cell Carcinoma | Left Hypothenar Eminence (ST) | Yes | Amputation/ Excision | 9 Months |
| Beach & Somer^A376^ | 2012 | 79/M | Kidney Cancer | Clear Cell Carcinoma | Right 3MP | Yes | NM | NM |
| Borgohain et al.^A377^ | 2012 | 70/M | Kidney Cancer | Clear Cell Carcinoma | Right Trapezium, Trapezoid, 2MC | No | Declined Treatment | NM |
| Hernández-Cortés et al.^A378^ | 2016 | 53/M | Kidney Cancer | Clear Cell Carcinoma | Right 5DP | Yes | Amputation/ Excision | 3 Months |
| Humphries et al.^A379^ | 2016 | 69/M | Kidney Cancer | Clear Cell Carcinoma | Right Wrist (ST) | Yes | Radiation | Alive At Time of Publication |
| Kumar^A380^ | 2016 | 46/M | Kidney Cancer | Clear Cell Carcinoma | Left 5DP | Yes | Radiation | NM |
| Selvi et al.^A381^ | 2016 | 51/M | Kidney Cancer | Clear Cell Carcinoma | Left 5DP | Yes | Amputation/ Excision | 6 Months |
| Zyluk & Janowski^A382^ | 2016 | 72/M | Kidney Cancer | Clear Cell Carcinoma | Left Triquetrum, Trapezoid, Capitate, Hamate, 3MC, 4MC, 5MC | Yes | Amputation/ Excision | Alive At Time of Publication |
| Lechmiannandan et al.^A383^ | 2018 | 52/M | Kidney Cancer | Clear Cell Carcinoma | Right 1DP | Yes | Amputation/ Excision | 6 Months |
| Oshina & Azuma^A384^ | 2018 | 83/F | Kidney Cancer | Clear Cell Carcinoma | Left 3DP | Yes | Amputation/ Excision | NM |
| Ho et al.^A385^ | 2020 | 55/F | Kidney Cancer | Clear Cell Carcinoma | Right 4DP | No | Amputation/ Excision | NM |
| Milionis et al.^A386^ | 2020 | 76/M | Kidney Cancer | Clear Cell Carcinoma | Right "D2" | Yes | Conservative/ Palliative Treatment | NM |
| Rao^A387^ | 2020 | 55/M | Kidney Cancer | Clear Cell Carcinoma | Left 1DP | No | Radiation | Lost to Follow-up |
| Dow et al. | 2021 | 78/M | Kidney Cancer | Clear Cell Carcinoma | Left 4PP | Yes | Amputation/ Excision | 24 Months |
| Greither & Tritsch^A388^ | 1957 | NM/NM | Kidney Cancer | NM | Right 4DP | NM | NM | NM |
| Mangini^A240^ | 1967 | 57/F | Kidney Cancer | NM | Right 5MP | No | Amputation/ Excision | NM |
| Barnett & Morris^A389^ | 1969 | 56/M | Kidney Cancer | NM | Right 2DP | Yes | Amputation/ Excision | NM |
| Bunnell^A390^ | 1970 | 77/F | Kidney Cancer | NM | NS 3PP | NM | NM | NM |
| Warda et al.^A391^ | 1974 | 59/F | Kidney Cancer | NM | NS "D3" | NM | NM | 6 Months |
| Brüchle et al.^A392^ | 1977 | NM/F | Kidney Cancer | NM | Left 4MC, 5MC | Yes | Amputation/ Excision | 20 Months |
| Brüchle et al.^A393^ | 1977 | NM/NM | Kidney Cancer | NM | NS Not mentioned | NM | NM | NM |
| Wu & Guise^A187^ | 1978 | 66/F | Kidney Cancer | NM | Left Dorsum of Hand (ST) | No | Conservative/ Palliative Treatment | 4 Months |
| Morris & House^A131^ | 1985 | 45/M | Kidney Cancer | NM | Left 2PP | NM | NM | 6 Months |
| Troncoso et al.^A393^ | 1991 | 53/M | Kidney Cancer | NM | Left 2DP | Yes | Amputation/ Excision | 7 Months |
| Bibi et al.^A394^ | 1993 | 60/M | Kidney Cancer | NM | Right 5DP | Yes | Amputation/ Excision | Alive At Time of Publication |
| Kierney et al.^A395^ | 1994 | NM/NM | Kidney Cancer | NM | Left "D2" | NM | NM | NM |
| Sidhu et al.^A396^ | 1994 | 71/M | Kidney Cancer | NM | Left 5DP | Yes | Amputation/ Excision | 2 Months |
| Abrahams^A60^ | 1995 | 63/M | Kidney Cancer | NM | Right 4MC | No | NM | NM |
| Castello et al.^A5^ | 1996 | 65/M | Kidney Cancer | NM | Left 4PP, 3MC | No | NM | Alive At Time of Publication |
| Giberti et al.^A397^ | 1999 | 52/M | Kidney Cancer | NM | Right 1MC | Yes | Amputation/ Excision + Radiation | NM |
| Filloux & Fontaine^A21^ | 2000 | 76/M | Kidney Cancer | NM | Right "Metacarpals", "Carpals", Pulp D2 (ST), Pulp D3 (ST) | No | Amputation/ Excision | 17 Months |
| Fusetti et al.^A398^ | 2003 | 69/M | Kidney Cancer | NM | Left 3MP | Yes | Conservative/ Palliative Treatment | 15 Months |
| Riter & Ghobrial^A399^ | 2004 | 53/F | Kidney Cancer | NM | Left 2DP,  Right 2DP | Yes | Amputation/ Excision | NM |
| Salesi et al.^A400^ | 2007 | 74/M | Kidney Cancer | NM | Left 2PP,  Right 1MC | Yes | Amputation/ Excision | Alive At Time of Publication |
| Pugliese & Pagliuca^A401^ | 2011 | 89/M | Kidney Cancer | NM | Right 4MP | Yes | Amputation/ Excision | Alive At Time of Publication |
| Sonoda et al.^A402^ | 2011 | 70/F | Kidney Cancer | NM | Right 2MP | Yes | NM | NM |
| Rommer et al.^A284^ | 2014 | 66/F | Kidney Cancer | NM | Left 3DP,  Right 2DP | Yes | Amputation/ Excision | 4 Months |
| Muñoz-Mahamud et al.^A230^ | 2017 | 54/M | Kidney Cancer | NM | NS 1DP | Yes | Conservative/ Palliative Treatment | 2 Months |
| Nakagawa et al.^A403^ | 2017 | 52/M | Kidney Cancer | NM | Left Trapezium | Yes | Amputation/ Excision | Alive At Time of Publication |
| Abro et al.^A404^ | 2019 | 54/M | Kidney Cancer | NM | Left Pulp D5 (ST) | Yes | NM | Alive At Time of Publication |
| Panaiyadiyan et al.^A405^ | 2020 | 47/M | Kidney Cancer | NM | Left 2MC | No | Radiation | Alive At Time of Publication |
| Tan & Lateef^A406^ | 2020 | NM/NM | Kidney Cancer | NM | Left 4DP,  Right 3DP | Yes | Amputation/ Excision | Alive At Time of Publication |
| De Massary & Weil^A407^ | 1907 | 45/M | Kidney Cancer | Renal cell adenocarcinoma | Left Pulp D3 (ST), Pulp D4 (ST), Subungal D5 (ST),  Right Subungal D1 (ST), Subungal D3 (ST), Subungal D4 (ST) | No | Conservative/ Palliative Treatment | 1.5 Months |
| Kerin^A168^ | 1958 | 47/M | Kidney Cancer | Renal cell adenocarcinoma | Left 3DP, 5DP | No | Amputation/ Excision | 14 Months |
| Kovařík^A408^ | 1971 | 45/M | Kidney Cancer | Renal cell adenocarcinoma | Right 3DP | Yes | Amputation/ Excision | 4 Months |
| Drewes et al.^A190^ | 1981 | 63/M | Kidney Cancer | Renal cell adenocarcinoma | Right 1DP | Yes | Amputation/ Excision | 1 Month |
| Bunkis & Carter^A409^ | 1982 | 69/M | Kidney Cancer | Renal cell adenocarcinoma | Right 2PP | Yes | Radiation | 9 Months |
| Radó et al.^A410^ | 1982 | 71/M | Kidney Cancer | Renal cell adenocarcinoma | Left 1PP | No | Amputation/ Excision | Alive At Time of Publication |
| Battistelli et al.^A411^ | 1985 | 52/M | Kidney Cancer | Renal cell adenocarcinoma | Right 1PP | No | Conservative/ Palliative Treatment | 9 Months |
| Jebson et al.^A412^ | 1992 | 77/M | Kidney Cancer | Renal cell adenocarcinoma | Left Pulp D3 (ST) | Yes | Amputation/ Excision | 4 Months |
| Witthaut et al.^A413^ | 1994 | 61/F | Kidney Cancer | Renal cell adenocarcinoma | Left Thenar eminence (ST) | Yes | Radiation | Alive At Time of Publication |
| Blanes et al.^A414^ | 2003 | 67/F | Kidney Cancer | Renal cell adenocarcinoma | Left 2DP | Yes | NM | NM |
| Tan et al.^A415^ | 2012 | 71/M | Kidney Cancer | Renal cell adenocarcinoma | Right 4MC | No | Radiation | Alive At Time of Publication |
| Hayes et al.^A416^ | 1992 | 76/M | Kidney Cancer | Transitional cell | Right 1MC | No | Amputation/ Excision | 1 Month |
| Carando et al.^A417^ | 1951 | 39/M | Bladder Cancer | Malignant fibroepithelioma | Right 1DP | NM | NM | NM |
| Martín-Jiménez et al.^A418^ | 2019 | 72/M | Bladder Cancer | NM | Left 1DP | No | NM | NM |
| Assem et al.^A419^ | 2020 | 59/M | Bladder Cancer | Papillary Carcinoma | Right 4PP | No | Amputation/ Excision | 3 Months |
| Heymans et al.^A420^ | 1990 | 65/M | Bladder Cancer | Transitional cell | Right Dorsum of Hand (ST) | No | Amputation/ Excision | 4 Months |
| Marya et al.^A421^ | 1993 | 62/M | Bladder Cancer | Transitional cell | Left 1DP | Yes | Conservative/ Palliative Treatment | 1 Month |
| Walsh et al.^A422^ | 1994 | 46/M | Bladder Cancer | Transitional cell | Right 2DP | Yes | Amputation/ Excision | NM |
| Ozcanli et al.^A341^ | 2005 | 58/M | Bladder Cancer | Transitional cell | Left 1DP | Yes | Amputation/ Excision | NM |
| Taleb et al.^A423^ | 2011 | 46/F | Bladder Cancer | Transitional cell | Left 4MC | Yes | Amputation/ Excision | NM |
| Yoneda et al.^A424^ | 2013 | 64/M | Bladder Cancer | Transitional cell | Left 4DP | Yes | Amputation/ Excision | 3 Months |
| Bauer et al.^A425^ | 1997 | 66/F | Bladder Cancer | Undifferentiated carcinoma | Right Pisiform, Wrist (ST) | Yes | Amputation/ Excision | NM |
| **Cancers of the Reproductive Organs** | | | | | | | | |
| Martin & Dove^A193^ | 1983 | 59/F | Ovarian Cancer | Adenocarcinoma | Right 1DP | Yes | Amputation/ Excision | 3 Months |
| Turan et al.^A426^ | 1990 | 43/F | Ovarian Cancer | Endometrioid Carcinoma | Right 5MP | No | Amputation/ Excision | 0.25 Months |
| Falk et al.^A427^ | 2017 | 51/F | Ovarian Cancer | Endometrioid Carcinoma | Left 1DP | Yes | Amputation/ Excision | Poor Prognosis |
| Riba^A428^ | 1950 | 55/M | Testicular Cancer | Chorionepithelioma | Right 3DP | Yes | Radiation | 4 Months |
| Bell & Mason^A150^ | 1953 | 56/M | Testicular Cancer | Chorionepithelioma | Right Subungal D3 (ST) | Yes | NM | 3 Months |
| Gartmann^A429^ | 1958 | 63/M | Testicular Cancer | Seminoma | Left Pulp D4 (ST), Subungal D2 (ST) | Yes | Conservative/ Palliative Treatment | Alive At Time of Publication |
| Ornetti et al.^A430^ | 2012 | 68/F | Uterus Cancer | Adenocarcinoma | Right Pulp D2 (ST) | Yes | Declined Treatment | 6 Months |
| Hetzel et al.^A431^ | 1996 | 31/F | Uterus Cancer | Choriocarcinoma | Left 5DP | No | Chemotherapy/ Immunotherapy | Alive At Time of Publication |
| Afshar et al.^A432^ | 2007 | 33/F | Uterus Cancer | Choriocarcinoma | Right Pulp D5 (ST) | Yes | Amputation/ Excision | 12 Months |
| Sur et al.^A18^ | 2011 | 52/F | Uterus Cancer | Leiomyosarcoma | Right Pulp D2 (ST) | Yes | Amputation/ Excision | 1 Month |
| Marek & Vortel^A433^ | 1949 | 59/F | Uterus Cancer | NM | Left 1DP | NM | NM | NM |
| Nobuhara^A434^ | 1967 | 32/F | Uterus Cancer | NM | Left 5DP | Yes | Amputation/ Excision | 4 Months |
| Elamurugan et al.^A435^ | 2011 | 74/F | Uterus Cancer | Squamous cell carcinoma | Left Palm (ST) | Yes | Conservative/ Palliative Treatment | NM |
| Gold & Reefe^A19^ | 1963 | 76/M | Prostate Cancer | Adenocarcinoma | Left "Diffuse Bones",  Right "Diffuse Bones" | Yes | Chemotherapy/ Immunotherapy | 5 Months |
| Tully & Shirley^A436^ | 1972 | 59/M | Prostate Cancer | Adenocarcinoma | Left 2MC | No | Amputation/ Excision | 16 Months |
| García-Galaviz et al.^A437^ | 2018 | 66/M | Prostate Cancer | Adenocarcinoma | Right Subungal D4 (ST) | Yes | NM | 6 Months |
| Lander & O’Donnell^A438^ | 2021 | 59/M | Prostate Cancer | Adenocarcinoma | Right 3DP | Yes | Amputation/ Excision | 2 Months |
| Ruggiero & Borri^A439^ | 1955 | 54/M | Prostate Cancer | Clear Cell Carcinoma | Right 3DP | No | Amputation/ Excision | 2 Months |
| Schinz et al.^A440^ | 1952 | 60/M | Prostate Cancer | NM | Left 2DP,  Right 1PP | Yes | NM | NM |
| Brailsford^A119^ | 1953 | 50/M | Prostate Cancer | NM | Left "Fingers",  Right "Fingers" | Yes | NM | NM |
| Massraf &Wand^A414^ | 1998 | 62/M | Prostate Cancer | NM | Left 1DP | Yes | Amputation/ Excision | NM |
| Korsten^A442^ | 2019 | 71/M | Prostate Cancer | NM | Right 1MC, 2MC, 3MC, 4MC, 5MC, 1PP, 1DP, 2PP, 2MP, 2DP, 3PP, 3MP, 3DP, 4PP, 4MP, 4DP, 5PP, 5MP, 5DP | No | NM | NM |
| Nagano et al.^A443^ | 2016 | 63/M | Prostate Cancer | Unclear, anaplastic | Right Dorsum of Hand (ST) | Yes | Amputation/ Excision | 48 Months |
| Kumar^A294^ | 1975 | 45/F | Cervical Cancer | Adenocarcinoma | Left 2DP | Yes | Radiation | 19 Months |
| Gallardo-Alvarado et al.^A444^ | 2020 | 58/F | Cervical Cancer | Adenocarcinoma | Right 1PP | Yes | NM | 4 Months |
| Graham et al.^A180^ | 1973 | 80/F | Cervical Cancer | Squamous cell carcinoma | Right 3DP | No | Amputation/ Excision | NM |
| Dalicho et al.^A445^ | 1988 | 64/F | Cervical cancer | Squamous cell carcinoma | Right 5DP | Yes | Amputation/ Excision | 1 Month |
| Pertzborn et al.^A446^ | 2000 | 54/F | Cervical Cancer | Squamous cell carcinoma | Right Volar 3MP (ST) | Yes | Chemotherapy/ Immunotherapy | 0.5 Months |
| Muñoz-Mahamud et al.^A230^ | 2017 | 36/F | Cervical Cancer | Squamous cell carcinoma | Right 4DP | Yes | Amputation/ Excision | 4 Months |
| Folasire et al.^A447^ | 2019 | 85/F | Cervical Cancer | Squamous cell carcinoma | Left Palm (ST),  Right Pulp D1 (ST), Palm (ST) | Yes | Declined Treatment | 4 Months |
| Khosla et al.^A448^ | 2012 | 65/F | Vaginal Cancer | Squamous cell carcinoma | Left 4MC | Yes | Radiation | Alive At Time of Publication |
| Miyakawa et al.^A449^ | 1972 | 63/M | Penile Cancer | NM | Left Pulp D1 (ST) | Yes | NM | NM |
| Cegla et al.^A450^ | 2021 | 71/M | Penile Cancer | Squamous cell carcinoma | Left Wrist (ST) | Yes | Radiation | 18 Months |
| **Cancers of the Skin** | | | | | | | | |
| Gelberman et al.^A451^ | 1978 | 24/M | Melanoma | Melanocytes | Right 1MC | No | Amputation/ Excision | Alive At Time of Publication |
| Tochigi et al.^A452^ | 2000 | 56/F | Melanoma | Melanocytes | Left Dorsum of Hand (ST) | Yes | Amputation/ Excision | Alive At Time of Publication |
| Xavier et al.^A453^ | 2005 | 25/F | Melanoma | Melanocytes | Right Capitate | Yes | NM | "Expired" |
| Stahl et al.^A454^ | 2012 | 46/F | Melanoma | Melanocytes | Left Scaphoid | Yes | Amputation/ Excision | 13 Months |
| Muñoz-Mahamud et al.^A230^ | 2017 | 25/F | Melanoma | Melanocytes | Right Capitate | Yes | Conservative/ Palliative Treatment | "Expired" |
| Cattelan & Dumontier^A273^ | 2021 | 65/F | Melanoma | Melanocytes | NS Dorsum of Hand (ST) | Yes | Chemotherapy/ Immunotherapy | Alive At Time of Publication |
| Kumar et al.^A30^ | 2011 | 60/M | Skin (Inguinal) | Squamous cell carcinoma | Right 1DP | No | Radiation | NM |
| Kerin^A168^ | 1958 | 70/F | Skin (Second Toe) | Squamous cell carcinoma | Left 3PP | Yes | Amputation/ Excision | "Expired" |
| **Cancers of the Skeletal System** | | | | | | | | |
| Carroll et al.^A268^ | 1975 | NM/NM | Bone | Osteogenic Sarcoma | NS Dorsum of Hand (ST) | NM | NM | NM |
| Lambert et al.^A455^ | 1992 | 36/M | Bone (Femur) | Chondrosarcoma | Left Subungal D4 (ST),  Right Subungal D4 (ST) | Yes | Systemic Steroids | 0.25 Months |
| Ozcanli et al.^A341^ | 2005 | 40/M | Bone (Femur) | Chondrosarcoma | Right 4DP, Subgungal D4 (ST) | Yes | NM | NM |
| Cary et al.^A129^ | 1981 | 9/F | Bone (Femur) | Osteogenic Sarcoma | Left Scaphoid | Yes | Amputation/ Excision | NM |
| Froimson et al.^A456^ | 1967 | 30/M | Bone (Fibula) | Chondrosarcoma | Right Pulp D1 (ST), Pulp D2 (ST), Pulp D4 (ST) | Yes | Conservative/ Palliative Treatment | 3 Months |
| Ramseier et al.^A457^ | 2007 | 76/M | Bone (Foot) | Chondrosarcoma | Left Subungal D4 (ST),  Right Subungal D1 (ST) | Yes | Amputation/ Excision | Alive At Time of Publication |
| Wu & Guise^A187^ | 1978 | 43/M | Bone (Humerus) | Chondrosarcoma | Right Scaphoid, 2MC, 5MC | Yes | Conservative/ Palliative Treatment | 1.5 Months |
| Ozcanli et al.^A458^ | 2006 | 47/M | Bone (Humerus) | Chondrosarcoma | Left Thenar Eminence (ST), Pulp D1 (ST),  Right Thenar Eminence (ST), Pulp D1 (ST), Pulp D3 (ST) | Yes | Amputation/ Excision | 18 Months |
| Emori et al.^A459^ | 2014 | 71/F | Bone (Sacrum) | Metastatic Chordoma | Right Thenar Emenience (ST) | Yes | Amputation/ Excision | Alive At Time of Publication |
| Smith et al.^A460^ | 2018 | 59/M | Bone (Sacrum) | Metastatic Chordoma | Left 1DP, 4DP, 5DP | Yes | Radiation | NM |
| King et al.^A461^ | 1978 | 31/F | Bone (Scapula) | Chondrosarcoma | Right Pulp D1 (ST) | Yes | Radiation | 2 Months |
| Sanjay et al.^A462^ | 1988 | 10/M | Bone (Tibia) | Chondrosarcoma | Left 3MC | Yes | Declined Treatment | Poor Prognosis |
| **Cancers of the Blood** | | | | | | | | |
| Marcove & Charosky^A463^ | 1972 | 40/F | Hodgkin's Lymphoma (humerus) | B-cell | Right Pulp D3 (ST) | Yes | Radiation | 2 Months |
| Chin et al.^A464^ | 1998 | 66/F | Leukemia | B-cell Chronic Lymphocytic | Right Volar 3MP (ST) | Yes | Conservative/ Palliative Treatment | Alive At Time of Publication |
| Afshar & Ilkhanizadeh^A465^ | 2010 | 72/M | Leukemia | B-cell Chronic Lymphocytic | Right Pulp D1 (ST) | Yes | Amputation/ Excision | Alive At Time of Publication |
| Cruz et al.^A466^ | 2020 | 62/M | Leukemia | B-cell Chronic Lymphocytic | Right 2MC, 3MC, 4MC | Yes | Systemic Steroids | Alive At Time of Publication |
| Fino et al.^A467^ | 2012 | 47/M | Leukemia | Hairy Cell | Left Dorsum of Hand (ST) | Yes | Amputation/ Excision | NM |
| Morris & House^A131^ | 1985 | 70/F | Leukemia | Leukemia | Left 3MC | NM | NM | 6 Months |
| Chang et al.^A468^ | 1975 | 70/F | Leukemia | Monomyelocytic | Right 1DP | Yes | Amputation/ Excision | 0.36 Months |
| Roushdi et al.^A469^ | 2012 | 66/F | Lymphoma | B-cell | Left Hamate | No | Amputation/ Excision | Alive At Time of Publication |
| Meyerding^A470^ | 1925 | 53/F | Multiple Myeloma | Plasma Cell | Right "All Bones" | Yes | Conservative/ Palliative Treatment | 5 Months |
| Pobanz et al.^A471^ | 1955 | 57/F | Multiple Myeloma | Plasma Cell | Left 2MP, 3DP,  Right 3MP, 5MP | No | Amputation/ Excision | Alive At Time of Publication |
| **Other Cancers** | | | | | | | | |
| Cho et al.^A472^ | 2007 | 71/M | Ampulla of Vader | Adenocarcinoma | Left Pulp D1 (ST),  Right Pulp D3 (ST) | Yes | Declined Treatment | 8 Months |
| Kerin^A168^ | 1958 | 1.5/M | Brain Cancer | Neuroblastoma | Left "Diffuse Bones",  Right "Diffuse Bones" | Yes | Conservative/ Palliative Treatment | 2 Months |
| Reboul et al.^A120^ | 1960 | NM/NM | Brain Cancer | NM | Right "Carpals", 4MC, 5MC | NM | NM | NM |
| Reichert et al.^A473^ | 2001 | 29/M | Foot | Clear Cell Sarcoma | Left Capitate | Yes | Amputation/ Excision | 8 Months |
| Bubau et al.^A474^ | 2021 | 72/F | Foot | Pleomorphic Leimyosarcoma | Left Pulp D2 (ST), Pulp D4 (ST),  Right Pulp D2 (ST) | Yes | Amputation/ Excision | Alive At Time of Publication |
| Cuadros et al.^A475^ | 2021 | 56/M | Foot | Pleomorphic sarcoma | Right 5DP | Yes | Chemotherapy/ Immunotherapy | 10 Months |
| Diflo et al.^A476^ | 1992 | 66/M | Left Atrium | Myoxma | Right Wrist (ST) | Yes | Amputation/ Excision | Alive At Time of Publication |
| Frank & Pratt^A477^ | 1951 | 42/F | Mandible | Lymphosarcoma | Right 1MC | Yes | Radiation | 11 Months |
| Chen et al.^A478^ | 2008 | 41/F | Mediastinum | Angiosarcoma | Left Pulp D3 (ST) | Yes | Conservative/ Palliative Treatment | 2 Months |
| Baltazard et al.^A227^ | 2015 | 71/M | Pancreatic Cancer | Adenocarcinoma | Right 5DP | Yes | NM | 1 Month |
| Brownlow et al.^A479^ | 1999 | 61/M | Posterior Chest Wall | Malignant Fibrous Histoicytoma | Right Pulp D3 (ST) | Yes | Amputation/ Excision + Radiation | 10 Months |
| Kumar & Kovi^A244^ | 1978 | 21/M | Spine | Rhabdomyosarcoma | Right 3MC | No | Radiation | "Expired" |
| **Cancers with Unknown Primary** | | | | | | | | |
| Graham et al.^A180^ | 1973 | 63/M | Unknown primary | Adenocarcinoma | Left 3MP | No | Amputation/ Excision | Alive At Time of Publication |
| Chirodian et al.^A480^ | 1998 | 72/M | Unknown primary | Adenocarcinoma | Left 4DP | No | Amputation/ Excision | 1 Month |
| Giordano et al.^A481^ | 2019 | 77/M | Unknown primary | Adenocarcinoma | Left 4DP | No | Declined Treatment | 6 Months |
| Pantoja et al.^A127^ | 1976 | 20/M | Unknown primary | Chondrosarcoma | Left 4DP | NM | Amputation/ Excision + Radiation | NM |
| Cavit et al.^A136^ | 2018 | 47/M | Unknown primary | Chondrosarcoma | NS 1MC | NM | NM | NM |
| Vittali^A482^ | 1961 | 56/M | Unknown primary | Mixed carcinoma | Right 4PP | No | Amputation/ Excision | NM |
| Henkert & Berge^A363^ | 1991 | 52/M | Unknown primary | Poorly differentiated large-cell carcinoma | Right 5PP | No | Amputation/ Excision | 4 Months |
| Grilli^A266^ | 1958 | 58/M | Unknown primary | Unknown | Right 1MC | NM | NM | NM |

* M = Male, F = Female, NM = Not Mentioned, NS = No Side Mentioned, MC = Metacarpal, PP = Proximal phalanx, MP = Middle phalanx, DP = Distal phalanx, (ST) = indicates a soft tissue lesion

** ^A“reference number”^ = Citation for the reviewed article can be found in Appendix B

**Supplementary Table 1:** Summary of all cases of acrometastases of the hand and wrist sorted by primary cancer
